# Germaborenes: Borylene Transfer Agents for the Synthesis of Iminoboranes

**DOI:** 10.1002/chem.202004579

**Published:** 2020-12-23

**Authors:** Dominik Raiser, Hartmut Schubert, Holger F. Bettinger, Lars Wesemann

**Affiliations:** ^1^ Institut für Anorganische Chemie Eberhard Karls Universität Tübingen Auf der Morgenstelle 18 72076 Tübingen Germany; ^2^ Institut für Organische Chemie Eberhard Karls Universität Tübingen Auf der Morgenstelle 18 72076 Tübingen Germany

**Keywords:** borylene transfer agent, germaborenes, iminoboranes, main group chemistry

## Abstract

Halide and phenyl substituted germaborenes were shown to react with azides at room temperature and transfer a borylene moiety to give iminoboranes. This iminoborane synthesis based on a borylene transfer route was investigated computationally in the case of the phenyl substituted germaborene.

## Graphical abstract

**Germaborenes** act as borylene transfer agents to azides in the synthesis of iminoboranes.

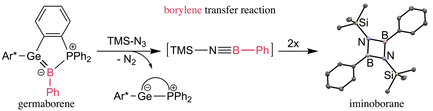

In borylene transfer chemistry sources of borylenes are almost exclusively transition metal borylene complexes.^[1]^ Following Braunschweig's ground breaking transfer of borylene ligands between transition metal centers, it was demonstrated that they can be transferred to alkynes resulting in borirenes.^[2]^ Also the synthesis of iminoboranes from reactions of transition metal borylene complexes with diorganodicarbodiimides was presented.[^2^, ^3^] More recently Braunschweig et al. reported that a transition metal free system, a phosphine stabilized diborane and a non‐stabilized diborane, can act as a borylene source in reaction with 2,2’‐bipyridine.^[4]^ We are studying the chemistry of germaborenes and report here on borylene transfer from germaborenes to organoazides to give iminoboranes.^[5]^

Halide substituted germaborenes **1**, **2** described previously were reacted at rt with organoazides RN_3_ (R=SiMe_3_; adamantyl, Ad) (Scheme 1).^[5a]^ A color change from deep red to yellow was observed and after extraction with *n*‐hexane and crystallization at −30 °C germyl substituted iminoboranes **3** and **4** were isolated and characterized by NMR spectroscopy and single crystal X‐ray diffraction. Short B−N bond lengths of 1.245(4) Å (**3**, Figure 1) and 1.231(5) Å (**4**, see Supporting Information) which can be compared with other B–N triple bonds [1.190(4)–1.265(7) Å] were found in both molecular structures.[^3c^, ^6^] The high bond order between boron and nitrogen is further indicated by the linear alignment of the Ge‐B‐N‐R fragments with two angles close to 180° [Ge‐B‐N: 168.0(3)° (**3**), 165.1(3)° (**4**); B‐N‐R: 174.6(2)° (**3**), 177.0(3)° (**4**)]. The signal in the ^31^P NMR spectrum of **3** was found at −9.6 ppm [**4**: −10.5 ppm] and in the ^11^B NMR spectrum of **3** at 2.8 ppm [**4**: 1.6 ppm]. The ^31^P and ^11^B NMR spectra of compounds **3’** and **4’** are shown in the Supporting Information.

**Scheme 1:**
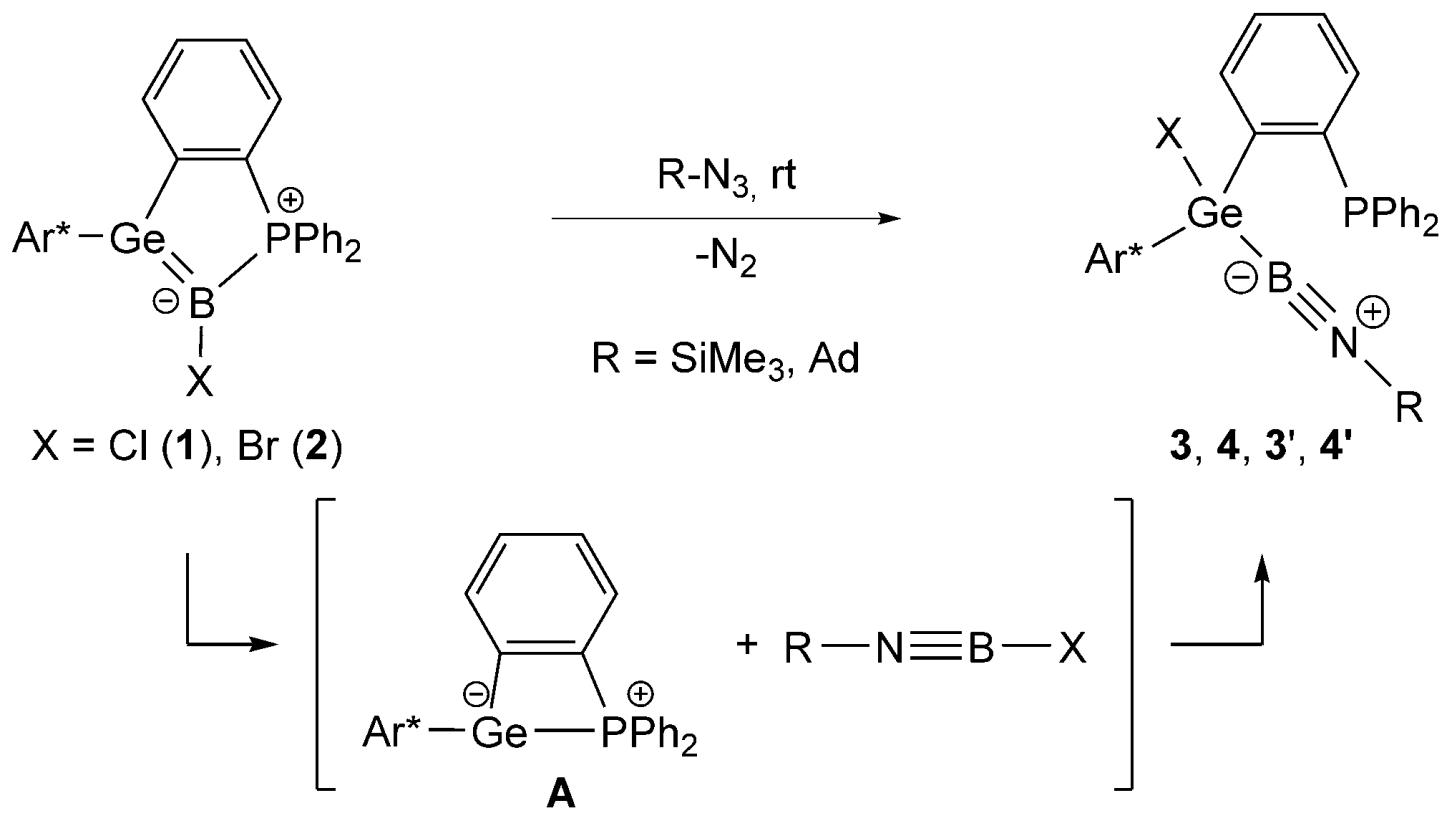
Reaction of halide substituted germaborenes **1** and **2** with organoazides. **3**: X=Cl, R=SiMe_3_ (X‐ray), **4**: X=Br, R=Ad (X‐ray), **3’**: X=Cl, R=Ad (NMR), **4’**: X=Br, R=SiMe_3_ (NMR), (Ar*=TripTp=2,6‐Trip_2_C_6_H_3_, Trip=2,4,6‐triisopropylphenyl).

**Figure 1:**
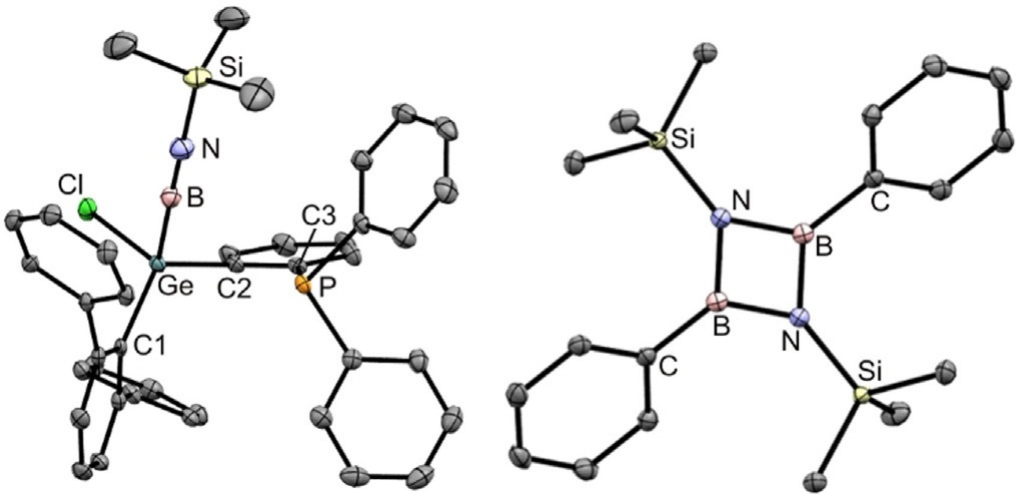
ORTEP^[7]^ of the molecular structure of **3** (left) and **8** [(PhBNSiMe_3_)_2_] (right). Ellipsoids set at 50 % probability. Hydrogen atoms and *i*Pr‐groups are omitted for clarity. Interatomic distances [Å] and angles [°] of **3**: B−N 1.245(4), N−Si 1.708(2), Ge−B 2.011(3), Ge−C1 1.953(2), Ge−C2 1.954(2), Ge−Cl 2.2119(6), C2−C3 1.407(3), P−C3 1.836(2); Si‐N‐B 174.6(2), N‐B‐Ge 168.0(3), C1‐Ge‐B 116.82(11), C2‐Ge‐B 108.16(11), Ge‐C2‐C3 120.05(18), C2‐C3‐P 117.87(18). Interatomic distances [Å] and angles [°] of **8**: B−N 1.4619(15), 1.4645(15), N−Si 1.7325(10), B−C 1.5622(16); N‐B‐N 98.56(9), B‐N‐B 81.44(9), Si‐N‐B 139.65(8), 137.34(8), C‐B‐N 130. 67(10), 130.76(10).

During the formation of germyl substituted iminoborane **3** the formation of germylene Lewis pair **A** was observed by ^31^P NMR spectroscopy (Scheme 1). Hence, it seems plausible that **3** was formed by transferring the borylene moiety ClB: from germaborene **1** to the azide forming intermediate iminoborane Me_3_Si‐N≡B‐Cl. This then reacts afterwards via an oxidative addition with germylene **A** to give germyl substituted iminoborane **3** (Scheme 1).

We reasoned that an oxidative addition with the intramolecular Lewis pair **A** could be prevented by using a germaborene with a less reactive carbon substituent. Therefore, the phenyl substituted germaborene **5** was synthesized following the analogous procedure used for the syntheses of **1** and **2**: oxidative addition of PhBCl_2_ at the germylene **A** (see Supporting Information, compound **6**) followed by reduction with Mg/anthracene. Following this straightforward procedure phenyl substituted germaborene **5** was isolated in high yield (309 mg, 86 % overall yield). An ORTEP of the molecular structure of **5** is placed in the Supporting Information. As in the case of halidegermaborenes **1** and **2** phenylgermaborene **5** exhibits an intramolecular photochemically activated reversible [2+2] cycloaddition with a phenyl moiety of the terphenyl substituent (see Supporting Information, compound **7**).^[5a]^ With phenylgermaborene **5** in hands the transfer of phenylborylene was studied (Scheme 2).

**Scheme 2:**
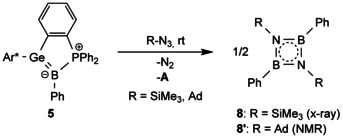
Reaction of phenyl germaborene **5** with organozides.

After addition of Me_3_SiN_3_ at rt the deep red solution had turned orange and a mixture of iminoborane dimer and trimer was characterized by ^11^B NMR spectroscopy with signals at 32.3 and 46.4 ppm (Scheme 2).^[8]^ The dimer of iminoborane **8** was obtained as colorless crystals from an *n*‐hexane solution at −40 °C and was characterized by X‐ray crystallography (Figure 1). The observed B−N bond lengths [1.4619(15) Å and 1.4645(15) Å] are in agreement with known iminoborane dimers.[^8e^, ^9^] The analogous phenylborylene transfer was also performed using AdN_3_. ^11^B NMR analysis suggest that the dimer **8’** was formed (Supporting Information). Phenylborylene which was transferred in the described examples was characterized in 2006 as a highly reactive species in inert gas matrices.^[10]^

Due to a Staudinger‐type side reaction between germylene phosphine Lewis pair **A** and organoazide two equivalents of azide were used in the reactions of phenylgermaborene.^[11]^ The characterization of the products **9** and **9’** of these side reactions were placed in the Supporting Information.

The transfer of PhB: to (CH_3_)_3_SiN_3_ was investigated computationally for derivative **5** at the B3LYP‐D3BJ/6‐31G*^[12]^ level of theory for exploration of the potential energy surface (PES). Subsequent energy refinement by single point evaluations used the linearly scaling domain based local pair natural orbital (DLPNO) method for an approximation to the canonical coupled‐cluster singles, doubles, and perturbative triples CCSD(T)/cc‐pVTZ.^[13]^ DLPNO‐CCSD(T) allows investigation of very large systems with an appropriate auxiliary basis set.^[14]^ The rigid‐rotor, harmonic oscillator model was used for determination of the Gibbs free energies at the B3LYP‐D3BJ/6‐31G* level which were added to the DLPNO‐CCSD(T) electronic energies (Figure 2). The reaction starts with an exergonic (Δ*G*=−28.7 kcal mol^−1^) concerted 1,3‐dipolar cycloaddition of (CH_3_)_3_SiN_3_ to the Ge=B double bond. This reaction has a low Gibbs free energy of activation Δ*G* ^≠^ of 12 kcal mol^−1^. The resulting five‐membered triazole corresponds to a very shallow minimum on the PES as the classical barrier for fragmentation to the iminoborane (PhBNSi(CH_3_)_3_), N_2_, and **A** is only 1.1 kcal mol^−1^, even disappears on the Δ*G* scale.

**Figure 2:**
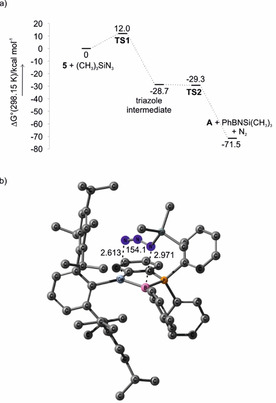
a) Gibbs free energy (in kcal mol^−1^) diagram for iminoborane formation from **5** and (CH_3_)_3_SiN_3_ as computed at the DLPNO‐CCSD(T)/cc‐pVTZ//B3LYP‐D3BJ/6‐31G* level of theory. b) Transition structure **TS1** as computed at the B3LYP‐D3BJ/6‐31G* level of theory. Hydrogen atoms were omitted for clarity, distances Ge−N and B−N are given in Å, the N‐N‐N angle is given in degrees.

Overall, the borylene transfer reaction is strongly exergonic, Δ*G*=−71.5 kcal mol^−1^. The computations thus support the experimental observation of a fast reaction of **5** with (CH_3_)_3_SiN_3_ and suggest a very facile fragmentation of the reactive intermediate.

To conclude, halide and phenyl substituted germaborenes react as main group element based borylene sources in reactions with organoazides. Thus, a straightforward synthetic room temperature route to iminoboranes was presented.

## Conflict of interest

The authors declare no conflict of interest.

## Supporting information

As a service to our authors and readers, this journal provides supporting information supplied by the authors. Such materials are peer reviewed and may be re‐organized for online delivery, but are not copy‐edited or typeset. Technical support issues arising from supporting information (other than missing files) should be addressed to the authors.

Supplementary
