# Supplementary material for: Germaborenes: Borylene Transfer Agents for the Synthesis of Iminoboranes
Source: Chemistry. 2020 Dec 23;27(6):1981–3. doi: 10.1002/chem.202004579 (PMC7898399; doi:10.1002/chem.202004579)
Supplement: Supplementary file 1 — Supplementary [file CHEM-27-1981-s001.pdf]

Chemistry–A European Journal

Supporting Information

Germaborenes: Borylene Transfer Agents for the Synthesis of Iminoboranes

Dominik Raiser,[a] Hartmut Schubert,[a] Holger F. Bettinger,[b] and Lars Wesemann*[a]

Content

Experimental Details 2
General Information 2
Reaction schemes 10
Reactions of germaborenes with azides 10
Synthesis of germaborene **5** 11
Reversible [2+2] cycloaddition of phenylgermaborene **5** 11
NMR Spektren 12
NMR spectra of compound **3** 12
NMR spectra of the reaction mixture of **3’** 17
NMR spectra of compound **4** 19
NMR spectra of the reaction mixture of **4’** 23
NMR spectra of compound **5** 24
NMR spectra of compound **6** 28
NMR spectra of compound **7** 32
NMR spectra of compound **8** 36
NMR spectra of the reaction mixture of **8’** 41
NMR spectra of compound **9** 43
NMR spectra of the reaction mixture of **9’** 47
^{1}H NMR spectra and UV-VIS spectra of reversible [2+2] cycloaddition 48
Crystallography 50
Computational Methods 57
Cartesian Coordinates 57
References 71

Experimental Details

General Information

All manipulations were carried out under argon atmosphere using standard Schlenk techniques or an MBraun Glovebox. Benzene, toluene and tetrahydrofuran were distilled from sodium. Hexane and pentane were obtained from a MBRAUN solvent purification system and degassed by three times freeze pump thaw. Benzene-d_6, toluene-d_8 and tetrahydryfuran-d_8 were distilled from sodium and stored over molecular sieves. Terphenyl-Ge(II) chloride (Ar*GeCl), Mg anthracene, compound **1** and **2** were prepared according to literature procedures.[1] Furthermore, chemicals were purchased commercially and used as received. Elemental analysis was performed by the Institut für Anorganische Chemie, Universität Tübingen using a Vario MICRO EL analyser.

NMR spectra were recorded with either a Bruker Avance III HD 300 NanoBay spectrometer equipped with a 5 mm BBFO probe head and operating at 300.13 (^{1}H), 75.47 (^{13}C), 121.49 (^{31}P) and 96.29 (^{11}B) MHz, a Bruker AvanceII+400 NMR spectrometer equipped with a 5 mm QNP (quad nucleus probe) head and operating at 400.13 (^{1}H), 100.62 (^{13}C), 161.97 (^{31}P) MHz, a Bruker AVII+ 500 NMR spectrometer with a variable temperature set up and a 5 mm TBO probe head and operating at 500.13 (^{1}H), 125.76 (^{13}C), 202.47 (^{31}P) and 160.46 (^{11}B) MHz, a Bruker Avance III HDX 600 NMR spectrometer with a 5 mm Prodigy BBO cryo probe head operating at 600.13 (^{1}H), 150.90 (^{13}C), 242.94 (^{31}P) and 192.55 (^{11}B) MHz or a Bruker Avance III HDX 700 NMR spectrometer with a 5 mm Prodigy TCI cryo probe head operating at 700.29 (^{1}H), 176.10 (^{13}C) MHz. Chemical shifts are reported in δ values in ppm relative to external standard TMS (^{1}H, ^{13}C), 85% aqueous H_3PO_4 (^{31}P) and referenced in most cases on the residual proton signal of the solvent C_6D_6 (^{1}H 7.15 ppm; ^{13}C 128.0 ppm). ^{31}P-, ^{11}B- as well as ^{1}H and ^{13}C-spectra in toluene-d_8, benzene-d_6, tetrahydrofuran-d_8 were referenced using the chemical shift of the solvent ^{2}H resonance frequency and Ξ = 25.145020% for ^{13}C, Ξ = 40.480742% for ^{31}P, Ξ = 32.083974% ^{11}B.[2] The multiplicity of the signals is abbreviated as s = singlet, d = doublet, t = triplet, quint = quintet, sept = septet and m = multiplet or unresolved. The proton and carbon signals were assigned by detailed analysis of ^{1}H, ^{13}C{^{1}H} or ^{13}C{^{1}H} UDEFT, ^{1}H-^{1}H COSY, ^{1}H-^{13}C HSQC, ^{1}H-^{13}C HMBC and ^{13}C{^{1}H} DEPT 135 spectra. Selected 1D-NMR spectra of the compounds and mixtures can be found in the Supporting Information.

UV/vis measurements were performed on a PerkinElmer Lambda 35 instrument. IR spectroscopy was performed using a Bruker Vertex 70 spectrometer and previously vacuum dried KBr.

Synthesis of compound (o-PPh_2)C_6H_4(Ar*)GeClBNSiMe_3 **3**

(o-PPh_2)C_6H_4(Ar*)GeBCl **1** (12.0 mg, 13.9 µmol, 1.00 eq) was dissolved in C_6D_6 (0.4 mL) and then Me_3SiN_3 (1.85 µL, 13.9 µmol, 1.00 eq) was added. The deep red solution became yellow and formation of the product **3** (about 89 %) was observed by ^{31}P NMR spectroscopy. Colourless crystals of **3** suitable for X-ray crystallography were obtained from a concentrated n-hexane solution at −40 °C (4.7 mg, 4.9 µmol, 36%). **^{1}H NMR** (600.13 MHz, 233.0 K, C_7D_8): δ (ppm) 7.46-7.39 (m, 3 H, C_6H_5), 7.30 (s, 1 H, m-C_6H_2), 7.28 (s, 1 H, m-C_6H_2), 7.25 (s, 1 H, m-C_6H_2), 7.22-7.15 (m, 4 H, aryl-H), 7.12-7.10 (m, 1 H, aryl-H), 7.05-7.00 (m, 2 H, aryl-H, overlapping solvent signal), 6.97-6.90 (m, 7 H, aryl-H, overlapping solvent signal), 6.58 (s, 1 H, m-C_6H_2), 3.70 (sept, 1 H, CH(CH_3)$_2$, $^3J_{HH}$ = 6.3 Hz), 3.51 (sept, 1 H, CH(CH_3)$_2$, $^3J_{HH}$ = 6.5 Hz), 2.99 (sept, 1 H, CH(CH_3)$_2$, $^3J_{HH}$ = 6.6 Hz), 2.93 (sept, 1 H, CH(CH_3)$_2$, $^3J_{HH}$ = 6.3 Hz), 2.80 (sept, 1 H, CH(CH_3)$_2$, $^3J_{HH}$ = 6.7 Hz), 2.08 (sept, 1 H, CH(CH_3)$_2$, overlapping solvent

signal), 1.77 (d, 3 H, CH(C*H*$_3$)$_2$, $^3J_{HH}$ = 7.0 Hz), 1.74 (d, 3 H, CH(C*H*$_3$)$_2$, $^3J_{HH}$ = 6.1 Hz), 1.49 (d, 3 H, CH(C*H*$_3$)$_2$, $^3J_{HH}$ = 6.1 Hz), 1.40 (d, 3 H, CH(C*H*$_3$)$_2$, $^3J_{HH}$ = 5.7 Hz), 1.36 (d, 3 H, CH(C*H*$_3$)$_2$, $^3J_{HH}$ = 6.8 Hz), 1.33 (d, 3 H, CH(C*H*$_3$)$_2$, $^3J_{HH}$ = 6.6 Hz), 1.26 (d, 3 H, CH(C*H*$_3$)$_2$, $^3J_{HH}$ = 7.6 Hz), 1.25 (d, 3 H, CH(C*H*$_3$)$_2$, $^3J_{HH}$ = 7.6 Hz), 1.06 (br s, 6 H, CH(C*H*$_3$)$_2$), 0.88 (d, 3 H, CH(C*H*$_3$)$_2$, $^3J_{HH}$ = 6.4 Hz), −0.26 (s, Si(C*H*$_3$)$_3$). −0.89 (d, 3 H, CH(C*H*$_3$)$_2$, $^3J_{HH}$ = 5.9 Hz). **^{13}C{^{1}H} NMR** (150.90 MHz, 233.0 K, C$_7$D$_8$): δ (ppm) 147.8 (d, *C*-aryl, J_{PC} = 56.3 Hz), 147.8 (s, *C*-aryl), 147.7 (s, *C*-aryl), 146.8 (s, *C*-aryl), 146.1 (s, *C*-aryl), 145.9 (s, *C*-aryl), 145.6 (s, *C*-aryl), 144.9 (s, *C*-aryl), 141.4 (s, *C*-aryl), 137.5 (s, *C*-aryl), 137.0 (s, *C*-aryl), 136.6-135.9 (m, *C*-aryl, overlapping solvent signal), 135.6 (s, *C*-aryl), 134.8 (d, *C*-aryl, J_{PC} = 19.1 Hz), 134.4 (d, *C*-aryl, J_{PC} = 21.4 Hz), 133.0 (s, *C*-aryl), 132.9 (d, *C*-aryl, J_{PC} = 19.7 Hz), 132.2 (s, *C*-aryl), 131.7 (d, *C*-aryl, J_{PC} = 5.7 Hz), 131.6 (d, *C*-aryl, J_{PC} = 8.9 Hz), 129.2 (s, *C*-aryl), 129.1 (s, *C*-aryl), 128.1-126.2 (m, *C*-aryl, overlapping solvent signal), 124.7 (s, *C*-aryl), 123.5 (s, *C*-aryl), 120.7 (s, *m*-*C*$_6$H$_2$), 120.2 (s, *m-C*$_6$H$_2$), 119.6 (s, *m-C*$_6$H$_2$), 119.0 (s, *m-C*$_6$H$_2$), 33.8 (s, *C*H(CH$_3$)$_2$), 31.5 (5, *C*H(CH$_3$)$_2$, J = 5.5 Hz), 30.3 (s, *C*H(CH$_3$)$_2$), 30.0 (s, *C*H(CH$_3$)$_2$), 29.4 (s, *C*H(CH$_3$)$_2$), 25.5 (s, CH(*C*H$_3$)$_2$), 25.2 (s, CH(*C*H$_3$)$_2$), 25.0 (s, CH(*C*H$_3$)$_2$), 24.5 (d, CH(*C*H$_3$)$_2$, J = 8.8 Hz), 24.1 (s, CH(*C*H$_3$)$_2$), 24.0 (s, CH(*C*H$_3$)$_2$), 23.8 (s, CH(*C*H$_3$)$_2$), 23.5 (s, CH(*C*H$_3$)$_2$), 22.2 (s, CH(*C*H$_3$)$_2$), 21.8 (s, CH(*C*H$_3$)$_2$), 21.4 (s, CH(*C*H$_3$)$_2$), 18.6 (s, CH(*C*H$_3$)$_2$), 13.4 (s, CH(*C*H$_3$)$_2$), 0.4 (s, Si(*C*H$_3$)$_3$). **^{31}P{^{1}H} NMR** (242.93 MHz, 233.0 K, C$_7$D$_8$): δ (ppm) −9.6 (s). **^{11}B{^{1}H} NMR** (192.54 MHz, 233.0 K, C$_7$D$_8$): δ (ppm) 3.0 (br s). **^{29}Si NMR** (119.22MHz, 233.0 K, C$_7$D$_8$): δ (ppm) −10.2 (s). **IR** (solid): ν = 1904 cm^{-1}. **Anal.** Calced. (%) for C$_{57}$H$_{72}$GeBClNPSi: C 72.13, H 7.65, N 1.48; found: C 72.26, H 8.00, N 1.29.

Synthesis of compound (*o*-PPh$_2$)C$_6$H$_4$(Ar*)GeClBNAd **3’** (Ad = C$_{10}$H$_{15}$) was achieved in the same way. ^{31}P and ^{11}B NMR spectrum of the reaction are shown below. Only NMR scale reactions were performed without further purification of the products. Product signals in **^{31}P{^{1}H} NMR** (121.49 MHz, 298.2 K, C$_6$D$_6$): δ (ppm) −10.5 (s). **^{11}B{^{1}H} NMR** (96.29 MHz, 298.2 K, C$_6$D$_6$): δ (ppm) 2.7 (s).

Synthesis of compound (*o*-PPh$_2$)C$_6$H$_4$(Ar*)GeBrBNAd **4**

(*o*-PPh$_2$)C$_6$H$_4$(Ar*)GeBBr **2** (20.0 mg, 22.1 µmol, 1.00 eq) was dissolved in C$_6$D$_6$ (0.4 mL) and then AdN$_3$ (3.91 mg, 22.1 µmol, 1.00 eq) was added. The deep red solution became yellow and quantitative formation the product **4** was observed by ^{31}P NMR spectroscopy. The solvent was removed in vacuo and the residue washed with cold *n*-hexane (0.1 mL, −40 °C). More product **4** was obtained as a colourless powder in several fractions from the wash solution at −40 °C (15.2 mg, 14.4 µmol, 65%). Colourless crystals of **4** suitable for X-ray crystallography were obtained from a concentrated *n*-hexane solution with two drops of THF at −40 °C. **^{1}H NMR** (700.29 MHz, 233.0 K, C$_7$D$_8$): δ (ppm) 7.46-7.40 (m, 3 H, C$_6$*H*$_5$), 7.34 (s, 1 H, *m*-C$_6$*H*$_2$),

7.33 (s, 1 H, *m*-C_6H_2), 7.23 (s, 1 H, *m*-C_6H_2), 7.20-7.16 (m, 4 H, aryl-*H*), 7.11-7.09 (m, 1 H, aryl-*H*), 7.06 (s, 1 H, aryl-*H*, overlapping solvent signal), 6.97-6.93 (m, 8 H, aryl-*H*, overlapping solvent signal), 6.60 (s, 1 H, *m*-C_6H_2), 3.72 (sept, 1 H, C*H*$(CH_3)_2$, $^3J_{HH}$ = 6.6 Hz), 3.61 (sept, 1 H, C*H*$(CH_3)_2$, $^3J_{HH}$ = 6.5 Hz), 3.08 (sept, 1 H, C*H*$(CH_3)_2$, $^3J_{HH}$ = 6.8 Hz), 3.01 (sept, 1 H, C*H*$(CH_3)_2$, $^3J_{HH}$ = 6.6 Hz), 2.82 (sept, 1 H, C*H*$(CH_3)_2$, $^3J_{HH}$ = 6.7 Hz), 2.08 (sept, 1 H, C*H*$(CH_3)_2$, overlapping solvent signal), 1.83 (d, 3 H, $CH(CH_3)_2$, $^3J_{HH}$ = 6.5 Hz), 1.81 (d, 3 H, $CH(CH_3)_2$, $^3J_{HH}$ = 6.7 Hz), 1.77 (s, 3 H, $C_{10}H_{15}$), 1.54 (s, 1 H, $C_{10}H_{15}$), 1.49 (d, 3 H, $CH(CH_3)_2$, $^3J_{HH}$ = 7.3 Hz), 1.49 (d, 3 H, $CH(CH_3)_2$, $^3J_{HH}$ = 7.3 Hz), 1.54 (s, 3 H, $C_{10}H_{15}$), 1.42-1.37 (m, 6 H, $CH(CH_3)_2$), 1.31-1.24 (m, 10 H, aryl-*H*, $C_{10}H_{15}$, $CH(CH_3)_2$), 1.09 (d, 6 H, $CH(CH_3)_2$, $^3J_{HH}$ = 7.1 Hz), 1.49 (d, 4 H, $C_{10}H_{15}$, J_{HH} = 11.6 Hz), 0.90 (d, 3 H, $CH(CH_3)_2$, $^3J_{HH}$ = 6.7 Hz), −0.85 (d, 3 H, $CH(CH_3)_2$, $^3J_{HH}$ = 6.5 Hz). **^{13}C{^{1}H} NMR** (176.08 MHz, 233.0 K, C_7D_8): δ (ppm) 143.2 (s, *C*-aryl), 147.8 (s, *C*-aryl), 147.8 (s, *C*-aryl), 147.0 (s, *C*-aryl), 146.8 (s, *C*-aryl), 146.1 (s, *C*-aryl), 145.9 (s, *C*-aryl), 145.8 (s, *C*-aryl), 145.5 (s, *C*-aryl), 145.1 (s, *C*-aryl), 141.2 (s, *C*-aryl), 138.2 (s, *C*-aryl), 137.5 (s, *C*-aryl), 137.1 (d, *C*-aryl, J_{PC} = 12.0 Hz), 136.8 (s, *C*-aryl), 136.6-136.1 (m, *C*-aryl, overlapping solvent signal), 134.5 (d, C_6H_5, J_{PC} = 22.4 Hz), 132.5-131.9 (m, *C*-aryl), 129.1-128.9 (m, *C*-aryl), 128.1 (s, *C*-aryl), 127.2 (s, *C*-aryl), 127.1 (s, *C*-aryl), 127.1-126.8 (m, *C*-aryl, overlapping solvent signal), 126.2 (s, *C*-aryl), 124.6 (s, *C*-aryl), 124.3 (s, *C*-aryl), 123.7 (s, *C*-aryl), 121.2 (s, *m*-C_6H_2), 119.8 (s, *m*-C_6H_2), 119.6 (s, *m*-C_6H_2), 119.2 (s, *m*-C_6H_2), 46.7 (s, $C_{10}H_{15}$), 44.1 (s, $C_{10}H_{15}$), 40.2 (s, $C_{10}H_{15}$), 33.9 (s, $C_{10}H_{15}$), 32.8 (s, $CH(CH_3)_2$), 31.5 (s, $CH(CH_3)_2$), 30.5 (s, $CH(CH_3)_2$), 30.1 (s, $CH(CH_3)_2$), 29.7 (s, $CH(CH_3)_2$), 28.4 (s, $C_{10}H_{15}$), 25.7 (s, $CH(CH_3)_2$), 25.3 (s, $CH(CH_3)_2$), 25.2 (s, $CH(CH_3)_2$), 24.7 (s, $CH(CH_3)_2$), 24.3 (s, $CH(CH_3)_2$), 24.0 (s, $CH(CH_3)_2$), 23.6 (s, $CH(CH_3)_2$), 23.3 (s, $CH(CH_3)_2$), 22.9 (s, $CH(CH_3)_2$), 22.2 (s, $CH(CH_3)_2$), 22.1 (s, $CH(CH_3)_2$), 15.0 (s, $CH(CH_3)_2$). **^{31}P{^{1}H} NMR** (121.49 MHz, 298.2 K, C_7D_8): δ (ppm) −10.5 (s). **^{11}B{^{1}H} NMR** (160.46 MHz, 298.2 K, C_7D_8): δ (ppm) 1.4 (br s). **IR** (solid): ν = 1966 cm^{-1}. **Anal.** Calced. (%) for $C_{64}H_{78}GeBBrNP \cdot C_4H_8O$: C 72.42, H 7.69, N 1.24; found: C 72.57, H 8.18, N 1.66. **4** crystallizes with three equivalents of thf (see crystal data of **4**). Under vacuum only two equivalents thf could be removed and one equivalent thf is visible in the ^{1}H NMR spectra of **4**.

Synthesis of compound (*o*-PPh_2)C_6H_4(Ar*)GeBrBNSiMe$_3$ **4'** was achieved in the same way. ^{31}P spectrum of the reaction is shown below. Only NMR scale reactions were performed without further purifications. Product signal in **^{31}P{^{1}H} NMR** (121.49 MHz, 298.2 K, C_6D_6): δ (ppm) −9.4 (br s).

Synthesis of compound (*o*-PPh_2)C_6H_4(Ar*)GeBPh **5** (see Scheme S2)

First Magnesium (89.3 mg, 3.67 mmol, 10.0 eq) and five drops of dibromoethane were stirred in tetrahydrofuran (2 mL). After a few minutes anthracene (6.55 mg, 36.7 µmol, 0.10 eq) was

added and the reaction mixture was stirred again for a couple of minutes. Then (*o*-PPh_2)C_6H_4(Ar*)Ge(Cl)BClPh **6** (358 mg, 367 µmol, 1.00 eq) was dissolved in tetrahydrofuran (5 mL) and also added to the reaction mixture. During the reduction the color of the solution turned from colorless to deep red. After four hours the solvent was removed *in vacuo*. After extraction with benzene (5 mL) and washing with *n*-hexane (5 mL) the product **5** could be obtained as a red powder (309 mg, 342 µmol, 93%). Red single crystals of **5** suitable for X-ray crystallography were obtained from a concentrated *n*-hexane solution at −40 °C. **^{1}H NMR** (400.11 MHz, 298.2 K, C_6D_6): δ (ppm) 7.48-7.42 (m, 6 H, PC_6H_5, *m*-GeC_6H_3), 7.10 (dd, 1 H, *p*-GeC_6H_3, $^3J_{HH}$ = 7.9 Hz, $^3J_{HH}$ = 7.1 Hz), 7.29-7.26 (m, 1 H, *6*-GeC_6H_4), 7.15-7.11 (m, 6 H, *m*-C_6H_2, BC_6H_5, overlapping solvent signal), 7.07-7.03 (m, 2 H, BC_6H_5), 7.00-6.95 (m, 4 H, BC_6H_5, *5*-GeC_6H_4, PC_6H_5), 6.95-6.90 (m, 4 H, PC_6H_5), 6.85-6.81 (m, 1 H, *3*-GeC_6H_4), 6.62-6.58 (m, 1 H, *4*-GeC_6H_4), 3.24 (sept., 4 H, *o*-C*H*(CH_3)$_2$, $^3J_{HH}$ = 6.6 Hz), 2.83 (sept., 2 H, *p*-C*H*(CH_3)$_2$, $^3J_{HH}$ = 6.9 Hz), 1.24 (d, 12 H, *p*-CH(C*H*$_3$)$_2$, $^3J_{HH}$ = 6.8 Hz), 1.19 (d, 12 H, *o*-CH(C*H*$_3$)$_2$, $^3J_{HH}$ = 6.8 Hz), 1.09 (d, 12 H, *o*-CH(C*H*$_3$)$_2$, $^3J_{HH}$ = 6.9 Hz). **^{13}C{^{1}H} NMR** (100.06 MHz, 298.2 K, C_6D_6): δ (ppm) 155.3 (d, *1*-$GeC_6H_4$$^2J_{PC}$ = 27.3 Hz), 147.9 (s, *p*-C_6H_2), 147.5 (s, *o*-GeC_6H_3), 147.1 (s, *o*-C_6H_2), 145.4 (d, *i*-GeC_6H_3, $^3J_{PC}$ = 25.1 Hz), 139.0 (s, *i*-C_6H_2), 132.5 (d, PC_6H_5, J_{PC} = 10.4 Hz), 135.4 (s, BC_6H_5), 135.3 (s, BC_6H_5), 134.6 (d, *2*-GeC_6H_4, $^2J_{PC}$ = 82.6 Hz), 132.5 (d, PC_6H_5, $^2J_{PC}$ = 10.3 Hz), 131.6-131.3 (m, GeC_6H_4, PC_6H_5), 130.6.0 (s, PC_6H_5), 129.6-128.9 (m, GeC_6H_4, PC_6H_5), 128.3 (d, PC_6H_5, J_{PC} = 10.5 Hz), 128.2 (s, BC_6H_5), 128.1-127.3 (m, GeC_6H_3, overlapping solvent signal), 126.6 (s, BC_6H_5), 125.4-124.6 (m, GeC_6H_4, BC_6H_5, PC_6H_5), 120.5 (s, *m*-C_6H_2), 34.5 (s, *p*-CH(CH_3)$_2$), 30.8 (s, *o*-CH(CH_3)$_2$), 26.3 (s, *o*-CH(CH_3)$_2$), 24.0 (s, *p*-CH(CH_3)$_2$), 22.8 (s, *o*-CH(CH_3)$_2$). **^{31}P{^{1}H} NMR** (161.96 MHz, 298.2 K, C_6D_6): δ (ppm) 12.6 (br s). **^{11}B{^{1}H} NMR** (96.29 MHz, 298.2 K, C_6D_6): δ (ppm) 16.2 (s). **Anal.** Calced. (%) for $C_{60}H_{68}BGeP$: C 79.75, H 7.59; found: C 79.14, H 7.32.

Synthesis of compound (*o*-PPh_2)C_6H_4(Ar*)Ge(Cl)BPhCl **6** (see Scheme S2)

(o-PPh_2)C_6H_4GeAr* **A** (300 mg, 368 µmol, 1.00 eq) was dissolved in hexane (8 mL) and benzene (2 mL) before $PhBCl_2$ (47.9 µL, 368 µmol, 1.00 eq) was added to the solution. After stirring over night the clear orange reaction mixture turned to a light yellow suspension. The product **6** was isolated by filtration and washed with cold hexane (1 mL, −40 °C). More product **6** was obtained by crystallization from the concentrated *n*-hexane solution at −40 °C (331 mg, 340 µmol, 92%). Colorless single crystals suitable for X-ray crystallography were obtained from a concentrated *n*-hexane solution at −40 °C. **^{1}H NMR** (400.11 MHz, 298.2 K, C_6D_6): δ (ppm) 7.75-7.65 (m, 2 H, C_6H_5), 7.56 (d, 2 H, C_6H_5, *J* = 6.6 Hz), 7.27-7.21 (m, 1 H, GeC_6H_4), 7.16-7.12 (m, 3 H, *m*-C_6H_3, *p*-C_6H_3, overlapping solvent signal), 7.11-6.39 (m, 12 H, *m*-C_6H_2, GeC_6H_4, C_6H_5), 6.89-6.81 (m, 1 H, GeC_6H_4), 6.70-6.63 (m, 1 H, BC_6H_5), 6.61-6.53 (m, 2 H, BC_6H_5), 6.50-6.41 (m, 2 H, BC_6H_5), 3.55 (sept, 2 H, *o*-C*H*(CH_3)$_2$, $^3J_{HH}$ = 6.8 Hz), 3.17 (sept,

2 H, *o*-C*H*(CH$_3$)$_2$, $^3J_{HH}$ = 6.6 Hz), 2.86 (sept, 2 H, *p*-C*H*(CH$_3$)$_2$, $^3J_{HH}$ = 6.9 Hz), 1.33-1.27 (m, 18 H, *p*-CH(C*H*$_3$)$_2$, *o*-CH(C*H*$_3$)$_2$), 1.18 (d, 6 H, *o*-CH(C*H*$_3$)$_2$, $^3J_{HH}$ = 6.6 Hz), 1.19-1.13 (br s, 6 H, *o*-CH(C*H*$_3$)$_2$), 1.09 (d, 6 H, *o*-CH(C*H*$_3$)$_2$, $^3J_{HH}$ = 6.6 Hz). **^{13}C{^{1}H} NMR** (100.06 MHz, 298.2 K, C$_6$D$_6$): δ (ppm) 156.0 (d, *1*-Ge*C*$_6$H$_4$ $^2J_{PC}$ = 24.3 Hz), 148.1 (s, *p*-*C*$_6$H$_2$), 147.6 (s, *i*-*C*$_6$H$_2$), 147.5 (s, *i*-*C*$_6$H$_2$), 147.6 (s, *o*-*C*$_6$H$_2$), 139.9 (s, *o*-Ge*C*$_6$H$_3$), 139.0 (d, *i*-Ge*C*$_6$H$_3$, $^3J_{PC}$ = 16.0 Hz), 136.3 (d, Ge*C*$_6$H$_4$, J_{PC} = 13.3 Hz), 135.2 (d, P*C*$_6$H$_5$, J_{PC} = 6.0 Hz), 134.8 (d, P*C*$_6$H$_5$, J_{PC} = 7.7 Hz), 133.5 (d, B*C*$_6$H$_5$, J_{PC} = 7.7 Hz), 132.9-132.6 (m, P*C*$_6$H$_5$, Ge*C*$_6$H$_4$), 134.4 (d, P*C*$_6$H$_5$, J_{PC} = 3.3 Hz), 131.2 (d, P*C*$_6$H$_5$, J_{PC} = 2.8 Hz), 130.7 (d, B*C*$_6$H$_5$, J_{PC} = 2.8 Hz), 130.2 (d, *2*-Ge*C*$_6$H$_4$, $^1J_{PC}$ = 69.1 Hz), 128.2-127.3 (m, B*C*$_6$H$_5$, *m*-Ge*C*$_6$H$_3$, *p*-Ge*C*$_6$H$_3$, Ge*C*$_6$H$_4$, overlapping solvent signal), 127.6-127.0 (m, Ge*C*$_6$H$_4$), 126.5 (d, P*C*$_6$H$_5$, J_{PC} = 3.2 Hz), 125.5 (d, *i*-P*C*$_6$H$_5$, $^1J_{PC}$ = 58.6 Hz), 124.6 (d, *i*-P*C*$_6$H$_5$, $^1J_{PC}$ = 55.3 Hz), 121.4 (s, *m*-*C*$_6$H$_2$), 120.6 (s, *m*-*C*$_6$H$_2$), 34.1 (s, *p*-*C*H(CH$_3$)$_2$), 31.1 (s, *o*-*C*H(CH$_3$)$_2$), 30.7 (s, *o*-*C*H(CH$_3$)$_2$), 26.3 (s, *o*-CH(*C*H$_3$)$_2$), 25.5 (s, *o*-CH(*C*H$_3$)$_2$), 24.2 (s, *p*-CH(*C*H$_3$)$_2$), 23.9 (s, *p*-CH(*C*H$_3$)$_2$), 23.3 (s, *o*-CH(*C*H$_3$)$_2$), 22.8 (s, *o*-CH(*C*H$_3$)$_2$). **^{31}P{^{1}H} NMR** (161.96 MHz, 298.2 K, C$_6$D$_6$): δ (ppm) 9.3 (s). **^{11}B{^{1}H} NMR** (96.29 MHz, 298.2 K, C$_6$D$_6$): δ (ppm) −5.5 (s). **Anal.** Calced. (%) for C$_{60}$H$_{68}$BCl$_2$GeP: C 73.95, H 7.03; found: C 73.58, H 6.46.

Synthesis of compound (*o*-PPh$_2$)C$_6$H$_4${(Trip)C$_6$H$_3$(C$_6$H$_2$^{i}Pr$_3$GeBPh)} **7** (see Scheme S3)

(*o*-PPh$_2$)C$_6$H$_4$(Ar*)GeBPh **5** (21.0 mg, 23.2 µmol, 1.00 eq) was dissolved in benzene-d$_6$ (0.4 mL) and illuminated with a green LED (530 nm). After 10 hours the colour of the reaction mixture had changed from deep red to light yellow. ^{31}P and ^{11}B NMR spectra show a complete conversion of **5** to **7** and all steps were carried out under exclusion of light. The solvent was removed *in vacuo* and product **7** was obtained as a yellow powder (19.6 mg, 21.7 µmol, 98%). Yellow single crystals of **7** suitable for X-ray crystallography were obtained from a concentrated toluene solution at −40 °C. **^{1}H NMR** (700.29 MHz, 298.2 K, C$_6$D$_6$): δ (ppm) 7.76-7.73 (m, 1 H, *p*-GeC$_6$*H*$_3$), 7.65-7.60 (m, 2 H, C$_6$*H*$_5$), 7.37-7.32 (m, 3 H, *m*-GeC$_6$*H*$_3$, *m*-C$_6$*H*$_2$(Trip)), 7.31-7.27 (m, 2 H, C$_6$*H*$_5$), 7.23-7.18 (m, 1 H, C$_6$*H*$_4$), 7.11-7.01 (m, 8 H, C$_6$*H*$_4$, C$_6$*H*$_5$), 6.94-6.89 (m, 2 H, BC$_6$*H*$_5$), 6.88 (d, 1 H, *m*-C$_6$*H*$_2$(Trip), $^4J_{HH}$ = 1.7 Hz), 6.88-6.84 (m, 1 H, GeC$_6$*H*$_4$), 6.81-6.77 (m, 1 H, BC$_6$*H*$_5$), 6.75-6.71 (m, 1 H, BC$_6$*H*$_5$), 5.79 (s, 1 H, GeC$_6$*H*$_2$), 3.80 (sept, 1 H, *o*-C*H*(CH$_3$)$_2$(Trip), $^3J_{HH}$ = 6.8 Hz), 3.44 (sept, 1 H, *3*-C*H*(CH$_3$)$_2$(GeC$_6$H$_2$), $^3J_{HH}$ = 6.8 Hz), 3.10 (sept, 1 H, *o*-C*H*(CH$_3$)$_2$(Trip), $^3J_{HH}$ = 6.8 Hz), 2.80 (d, 1 H, *6*-GeC$_6$*H*$_2$, $^3J_{PH}$ = 24.2 Hz), 2.79 (sept, 1 H, *o*-C*H*(CH$_3$)$_2$(Trip), $^3J_{HH}$ = 6.9 Hz), 2.01 (sept, 1 H, *1*-C*H*(CH$_3$)$_2$(GeC$_6$H$_2$), 1.64 (sept, 1 H, *5*-C*H*(CH$_3$)$_2$(GeC$_6$H$_2$), 1.45 (d, 3 H, *o*-CH(C*H*$_3$)$_2$(Trip), $^3J_{HH}$ = 6.8 Hz), 1.37 (d, 3 H, *3*-CH(C*H*$_3$)$_2$(GeC$_6$H$_2$), $^3J_{HH}$ = 6.6 Hz), 1.36 (d, 3 H, *o*-CH(C*H*$_3$)$_2$(Trip), $^3J_{HH}$ = 6.8 Hz), 1.23 (d, 3 H, *p*-CH(C*H*$_3$)$_2$(Trip), $^3J_{HH}$ = 6.8 Hz), 1.20 (d, 3 H, *p*-CH(C*H*$_3$)$_2$(Trip), $^3J_{HH}$ = 6.8 Hz), 1.19 (d, 3 H, *5*-CH(C*H*$_3$)$_2$(GeC$_6$H$_2$), $^3J_{HH}$ = 5.5 Hz), 1.18 (d, 3 H, *o*-CH(C*H*$_3$)$_2$(Trip), $^3J_{HH}$ = 6.6 Hz), 0.92 (d, 3 H, *5*-CH(C*H*$_3$)$_2$(GeC$_6$H$_2$), $^3J_{HH}$ = 6.8 Hz), 0.88-0.84 (m, 9 H,

1-CH(C*H*$_3$)$_2$(GeC$_6$H$_2$), *o*-CH(C*H*$_3$)$_2$(Trip)), 0.30 (d, 3 H, *3*-CH(C*H*$_3$)$_2$(GeC$_6$H$_2$), $^3J_{HH}$ = 6.6 Hz). **^{13}C{^{1}H} NMR** (176.09 MHz, 298.2 K, C$_6$D$_6$): δ (ppm) 152.9 (d, *1*-Ge*C*$_6$H$_4$, $^2J_{PC}$ = 26.8 Hz), 151.3 (s, *o*-Ge*C*$_6$H$_3$), 151.1 (d, *5*-Ge*C*$_6$H$_2$, $^3J_{PC}$ = 3.9 Hz), 147.8 (s, *p*-*C*$_6$H$_2$(Trip)), 147.1 (s, *o*-*C*$_6$H$_2$(Trip)), 146.5 (d, *i*-Ge*C*$_6$H$_3$, $^3J_{PC}$ = 6.6 Hz), 146.4 (s, *o*-*C*$_6$H$_2$(Trip)), 130.4 (br s, P*C*$_6$H$_5$), 143.5 (s, *o*-Ge*C*$_6$H$_3$), 141.1 (s, *3*-Ge*C*$_6$H$_2$), 140.5 (s, *i*-*C*$_6$H$_2$(Trip)), 136.8 (d, P*C*$_6$H$_5$, J_{PC} = 8.3 Hz), 134.7 (d, Ge*C*$_6$H$_4$, J_{PC} = 12.4 Hz), 134.2 (d, Ge*C*$_6$H$_4$, J_{PC} = 73.5 Hz), 133.8 (d, B*C*$_6$H$_5$, J_{PC} = 19.9 Hz), 133.7 (d, B*C*$_6$H$_5$, J_{PC} = 8.1 Hz), 133.4 (d, Ge*C*$_6$H$_4$, J_{PC} = 5.3 Hz), 133.3 (s, *2*-Ge*C*$_6$H$_2$), 132.9 (d, P*C*$_6$H$_5$, J_{PC} = 8.8 Hz), 130.7-130.2 (m, Ge*C*$_6$H$_4$, P*C*$_6$H$_5$, B*C*$_6$H$_5$), 128.6 (d, P*C*$_6$H$_5$, J_{PC} = 9.7 Hz), 128.0 (s, B*C*$_6$H$_5$), 127.9-127.4 (m, *p*-Ge*C*$_6$H$_3$, Ge*C*$_6$H$_4$, B*C*$_6$H$_5$, overlapping solvent signal), 126.8 (s, P*C*$_6$H$_5$), 126.7 (s, *m*-Ge*C*$_6$H$_3$), 126.2 (d, P*C*$_6$H$_5$, J_{PC} = 47.8 Hz), 125.2-124.9 (m, *m*-Ge*C*$_6$H$_3$, P*C*$_6$H$_5$), 120.6 (s, *m*-*C*$_6$H$_2$(Trip)), 120.5 (s, *m*-*C*$_6$H$_2$(Trip)), 114.3 (s, *4*-Ge*C*$_6$H$_2$), 59.3 (d, *1*-Ge*C*$_6$H$_2$, $^3J_{PC}$ = 14.1 Hz), 36.3 (br s, *6*-GeC$_6$H$_2$(*C*H(CH$_3$)$_2$)), 34.5 (s, *p*-*C*H(CH$_3$)$_2$(Trip)), 34.2 (d, *1*-GeC$_6$H$_2$(*C*H(CH$_3$)$_2$), $^4J_{PC}$ = 2.5 Hz), 33.3 (s, *5*-GeC$_6$H$_2$(*C*H(CH$_3$)$_2$)), 30.6 (s, *o*-*C*H(CH$_3$)$_2$(Trip)), 30.1 (s, *o*-*C*H(CH$_3$)$_2$(Trip)), 28.7 (s, *3*-GeC$_6$H$_2$(*C*H(CH$_3$)$_2$)), 26.1 (s, *o*-CH(*C*H$_3$)$_2$(Trip)), 25.9 (s, *o*-CH(*C*H$_3$)$_2$(Trip)), 24.3 (s, *p*-CH(*C*H$_3$)$_2$(Trip)), 24.1 (s, *p*-CH(*C*H$_3$)$_2$(Trip)), 23.7 (s, *3*-GeC$_6$H$_2$(CH(*C*H$_3$)$_2$)), 23.7 (s, *o*-CH(*C*H$_3$)$_2$(Trip)), 23.5 (s, *o*-CH(*C*H$_3$)$_2$(Trip)), 23.4 (s, *5*-GeC$_6$H$_2$(CH(*C*H$_3$)$_2$)), 21.3 (s, *1*-GeC$_6$H$_2$(CH(*C*H$_3$)$_2$)), 21.0 (s, *1*-GeC$_6$H$_2$(CH(*C*H$_3$)$_2$)), 20.0 (s, *5*-GeC$_6$H$_2$(CH(*C*H$_3$)$_2$)), 19.5 (s, *3*-GeC$_6$H$_2$(CH(*C*H$_3$)$_2$)). **^{31}P{^{1}H} NMR** (121.49 MHz, 298.2 K, C$_6$D$_6$): δ (ppm) 22.4 (s). **^{11}B{^{1}H} NMR** (96.29 MHz, 298.2 K, C$_6$D$_6$): δ (ppm) −10.8 (s). **Anal.** Calced. (%) for C$_{60}$H$_{68}$BGeP · 2 C$_7$H$_8$: C 81.70, H 7.80; found: C 81.87, H 7.66. Two equivalents of toluene were also found in the crystal structure determination of **7**.

Synthesis of compound [C$_6$H$_5$BNSiMe$_3$]$_2$ **8**

(*o*-PPh$_2$)C$_6$H$_4$(Ar*)GeBPh **5** (50.0 mg, 55.3 µmol, 1.00 eq) was dissolved in toluene (0.2 mL) and then Me$_3$SiN$_3$ (7.34 µL, 55.3 µmol, 1.00 eq) was added. The deep red solution became orange and formation of **A** was observed by ^{31}P NMR spectroscopy. Dimer **8** was crystallized directly from the reaction mixture at −40 °C (4.1 mg, 23.4 µmol, 42%). Colourless crystals of **8** suitable for X-ray crystallography were obtained from a concentrated *n*-hexane solution at −40 °C. **^{1}H NMR** (400.11 MHz, 298.2 K, C$_7$D$_8$): δ(ppm) 7.75-7.70 (m, 2 H, C$_6$*H*$_5$), 7.27-7.20 (m, 3 H, C$_6$*H*$_5$), 0.07 (s, 9 H, Si(C*H*$_3$)$_3$). **^{13}C{^{1}H} NMR** (100.60 MHz, 298.2 K, C$_7$D$_8$): δ (ppm) 133.0 (s, *C*$_6$H$_5$), 130.4 (s, *C*$_6$H$_5$), 129.1 (s, *C*$_6$H$_5$, overlapping solvent signal), 127.9 (s, *C*$_6$H$_5$, overlapping solvent signal), 2.2 (s, Si(*C*H$_3$)$_3$) **^{11}B NMR** (160.46MHz, 298.2 K, C$_7$D$_8$): δ (ppm) 46.3 (br s, dimer), 32.3 (s, trimer),. **^{29}Si NMR** (119.22 MHz, 298.2 K, C$_7$D$_8$): δ (ppm) 3.0 (s, trimer), −1.5 (s, dimer). **Anal.** Calced. (%) for C$_9$H$_{14}$BNSi · C$_7$H$_8$: C 76.87, H 8.41, N 3.90; found: C 76.01, H 8.44, N 3.41.

Synthesis of compound C_6H_5BNAd **8'** was achieved in the same way but 2 equivalents of $C_{10}H_{15}N_3$ were needed as **A** seems to react faster with $C_{10}H_{15}N_3$ than **5**. ^{31}P and ^{11}B NMR spectrum of the reaction are shown below. Only NMR scale reactions were performed without further purification of the product. Product signals in **$^{11}B\{^1H\}$ NMR** (96.29 MHz, 298.2 K, C_6D_6): δ (ppm) 43.8 (br s), 24.8 (s).

Synthesis of compound (o-PPh_2)C_6H_4(Ar*)$GeNSiMe_3$ **9** (see Scheme S1)

(o-PPh_2)C_6H_4GeAr* **A** (30.0 mg, 36.8 µmol, 1.00 eq) was dissolved in C_6D_6 (0.4 mL) and then Me_3SiN_3 (4.88 µL, 36.8 µmol, 1.00 eq) was added. After 30 min the orange solution was more intense and conversion to product **9** was observed by ^{31}P NMR spectroscopy. After one week yellow single crystals suitable for X-ray crystallography were obtained from the concentrated reaction mixture at room temperature (18.6 mg, 20.6 µmol, 56%). **^1H NMR** (400.11 MHz, 298.2 K, C_7D_8): δ (ppm) 7.72-7.63 (m, 2 H, C_6H_5), 7.61-7.52 (m, 2 H, C_6H_5), 7.39-7.33 (m, 2 H, C_6H_4), 7.27 (d, 2 H, m-C_6H_2, $^4J_{HH}$ = 1.5 Hz), 7.15-7.13 (m, 3 H, C_6H_3), 7.11-7.04 (m, 2 H, C_6H_5, overlapping solvent signal), 7.03-6.98 (m, 2 H, C_6H_5, overlapping solvent signal), 6.96 (br s, 3 H, C_6H_5, C_6H_4, overlapping solvent signal), 6.94-6.87 (m, 3 H, C_6H_5, m-C_6H_2, overlapping solvent signal), 6.40 (d, 1 H, C_6H_4, J_{HH} = 7.0 Hz), 3.41 (sept, 2 H, o-$CH(CH_3)_2$, $^3J_{HH}$ = 6.8 Hz), 2.88 (sept, 2 H, p-$CH(CH_3)_2$, $^3J_{HH}$ = 7.0 Hz), 2.68 (sept, 2 H, o-$CH(CH_3)_2$, $^3J_{HH}$ = 6.7 Hz), 1.62 (d, 6 H, o-$CH(CH_3)_2$, $^3J_{HH}$ = 7.0 Hz), 1.33 (d, 12 H, p-$CH(CH_3)_2$, $^3J_{HH}$ = 6.8 Hz), 1.25 (d, 6 H, o-$CH(CH_3)_2$, $^3J_{HH}$ = 6.8 Hz), 1.03 (d, 6 H, o-$CH(CH_3)_2$, $^3J_{HH}$ = 6.7 Hz), 0.74 (br s, 6 H, o-$CH(CH_3)_2$), 0.74 (br s, 6 H, o-$CH(CH_3)_2$), −0.74 (s, 9 H, $Si(CH_3)_3$). **$^{13}C\{^1H\}$ NMR** (100.60 MHz, 298.2 K, C_7D_8): δ (ppm) 148.8 (d, C_6H_4, J_{PC} = 23.5 Hz), 148.3 (s, p-C_6H_2), 148.1(s, o-C_6H_2), 147.6(s, o-C_6H_2), 146.8 (s, o-GeC_6H_3), 139.9 (d, C_6H_4, J_{PC} = 79.1 Hz), 139.8 (d, i-GeC_6H_3, $^3J_{PC}$ = 10.4 Hz), 139.4 (s, i-C_6H_2), 135.6 (d, C_6H_5, J_{PC} = 102.0 Hz), 135.2 (d, C_6H_5, J_{PC} = 99.6 Hz), 133.2 (d, C_6H_4, J_{PC} = 16.5 Hz), 132.5 (d, C_6H_5, J_{PC} = 9.7 Hz), 132.3 (d, C_6H_5, J_{PC} = 9.8 Hz), 131.3-130.8 (m, C_6H_3, C_6H_5), 130.0 (d, C_6H_4, J_{PC} = 2.8 Hz), 129.1 (s, C_6H_5, overlapping solvent signal), 128.3-127.9 (m, C_6H_4, C_6H_3, C_6H_5), 127.3 (d, C_6H_4, J_{PC} = 8.5 Hz), 121.0 (s, m-C_6H_2), 120.6 (s, m-C_6H_2), 34.9 (s, p-$CH(CH_3)_2$), 31.8 (s, o-$CH(CH_3)_2$), 31.2 (s, o-$CH(CH_3)_2$), 26.2 (s, o-$CH(CH_3)_2$), 26.1 (s, o-$CH(CH_3)_2$), 24.6 (s, p-$CH(CH_3)_2$), 24.5 (s, p-$CH(CH_3)_2$), 24.1 (s, o-$CH(CH_3)_2$), 22.4 (s, o-$CH(CH_3)_2$), −0.7 (s, $Si(CH_3)_3$). **$^{31}P\{^1H\}$ NMR** (242.93MHz, 298.2 K, C_7D_8): δ (ppm) 30.2 (s). **^{29}Si NMR** (59.62 MHz, 298.2 K, C_7D_8): δ (ppm) 27.7 (s). **Anal.** Calced. (%) for for $C_{57}H_{72}GeNPSi$: C 75.83, H 8.04; found: C 75.51, H 7.64.

Synthesis of compound (o-PPh_2)C_6H_4(Ar*)GeNAd **9'** was achieved by adding AdN_3 to a solution of **A**. Only NMR reactions were performed without further purification of the product.

^{31}P NMR spectrum of the reaction is shown below. Product signal in **$^{31}P\{^1H\}$ NMR** (121.49 MHz, 298.2 K, C_6D_6): δ (ppm) 24.9 (s).

Reaction schemes

Reactions of germaborenes with azides

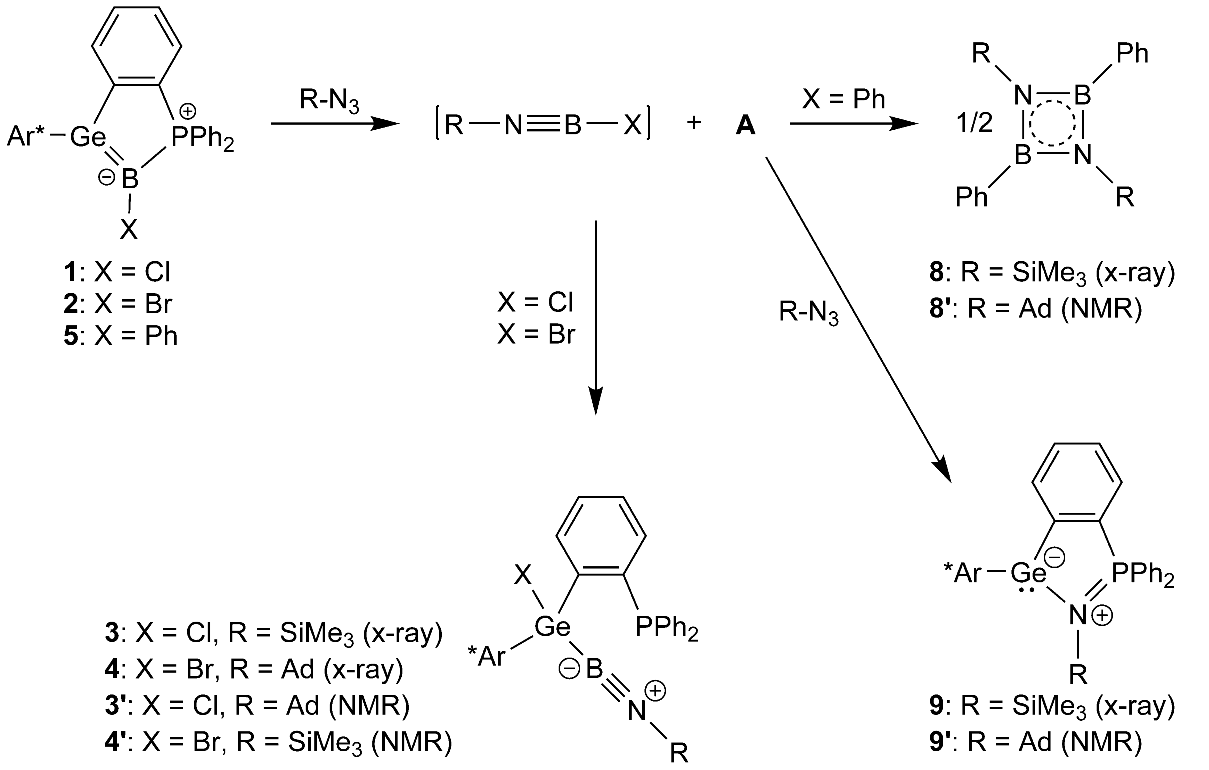

Scheme S1. Reactions of germaborenes **1**, **2** and **5** with azides and side products **9** and **9’** of these reactions.

Synthesis of germaborene **5**

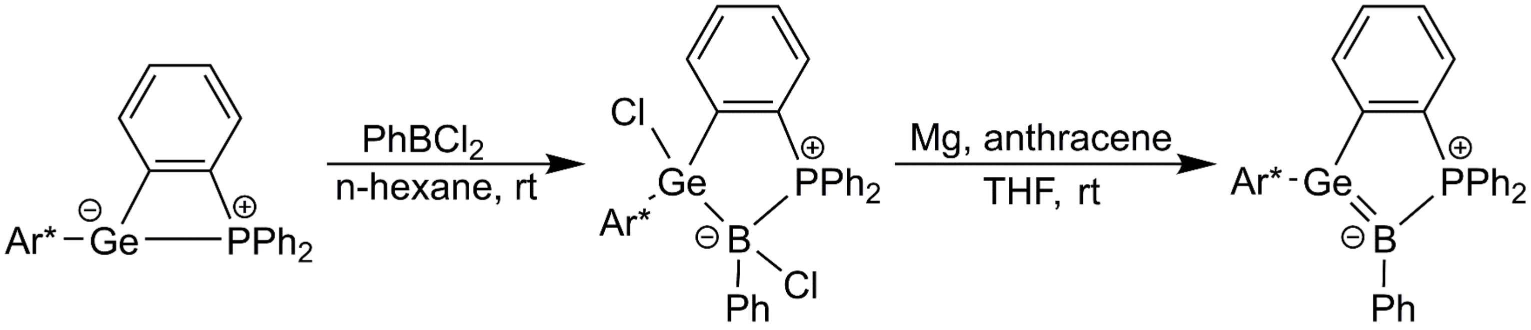

A **6** **5**

Scheme S2. Synthesis of germaborene **6**.

Reversible [2+2] cycloaddition of phenylgermaborene **5**

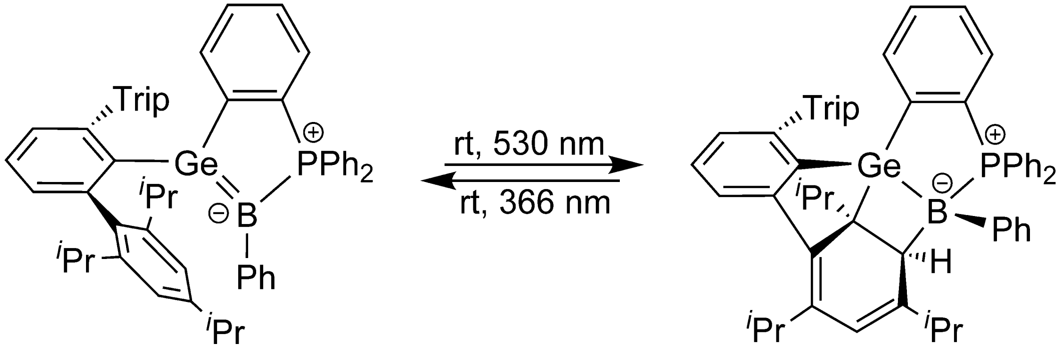

5 **7**

Scheme S3. Reversible [2+2] cycloaddition reaction of phenylgermaborene **5**.

NMR Spektren

NMR spectra of compound **3**

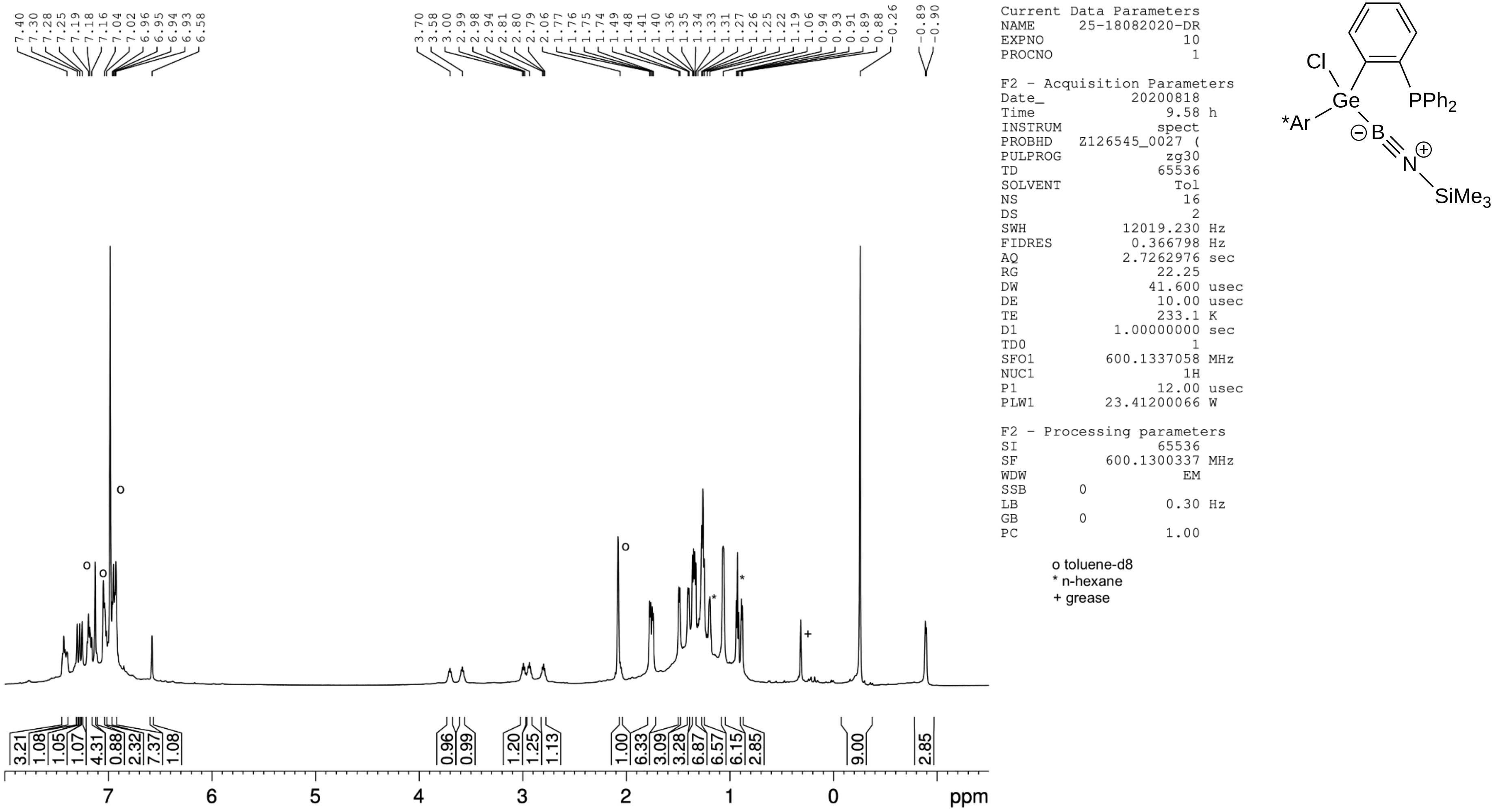

Figure S1. ^{1}H NMR (Tol-d_8) of compound **3**.

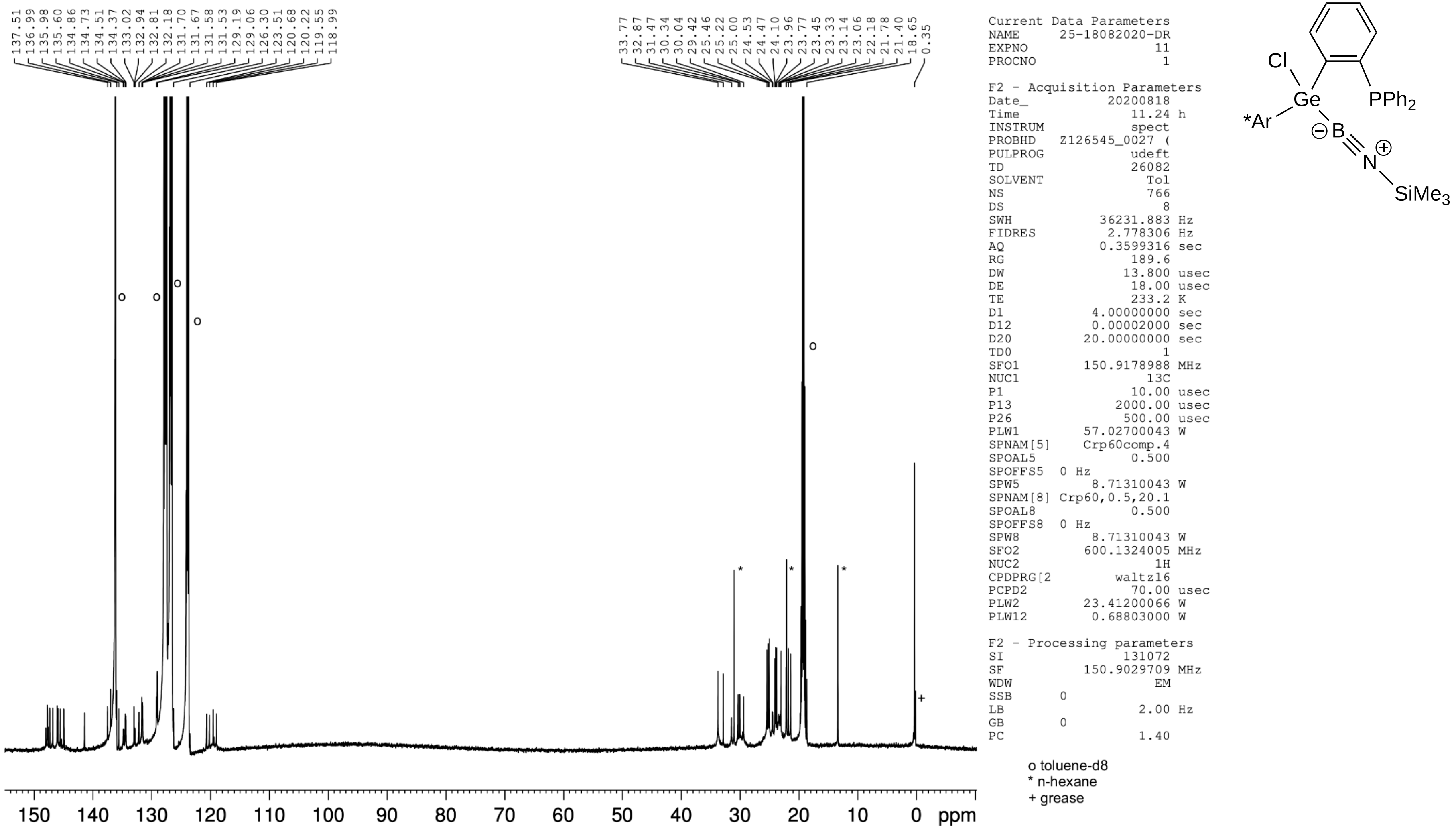

Figure S2. ^{13}C NMR (Tol-d_8) of compound **3**.

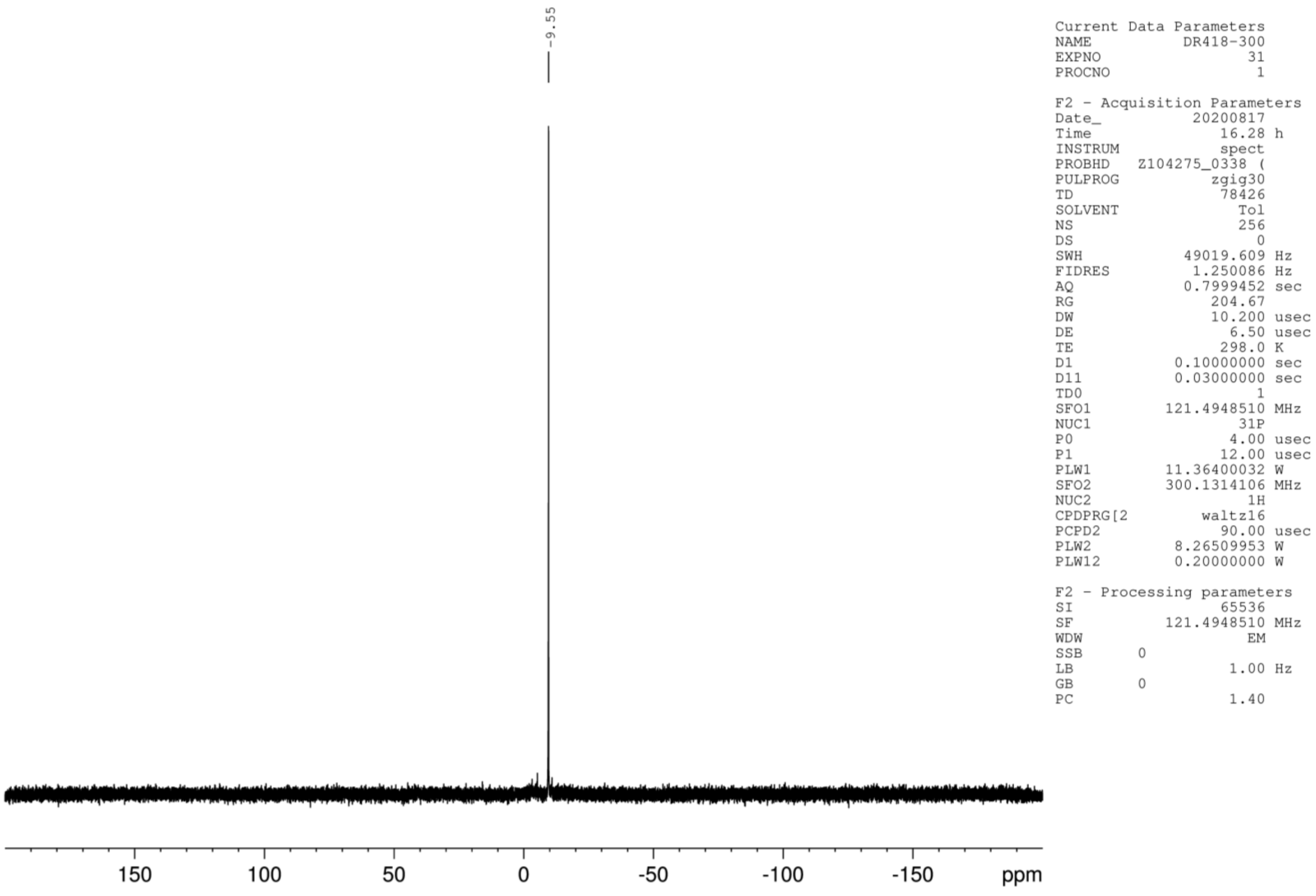

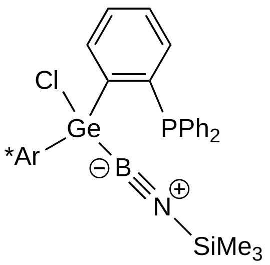

Figure S3. ^{31}P NMR (Tol-d_8) of compound **3**.

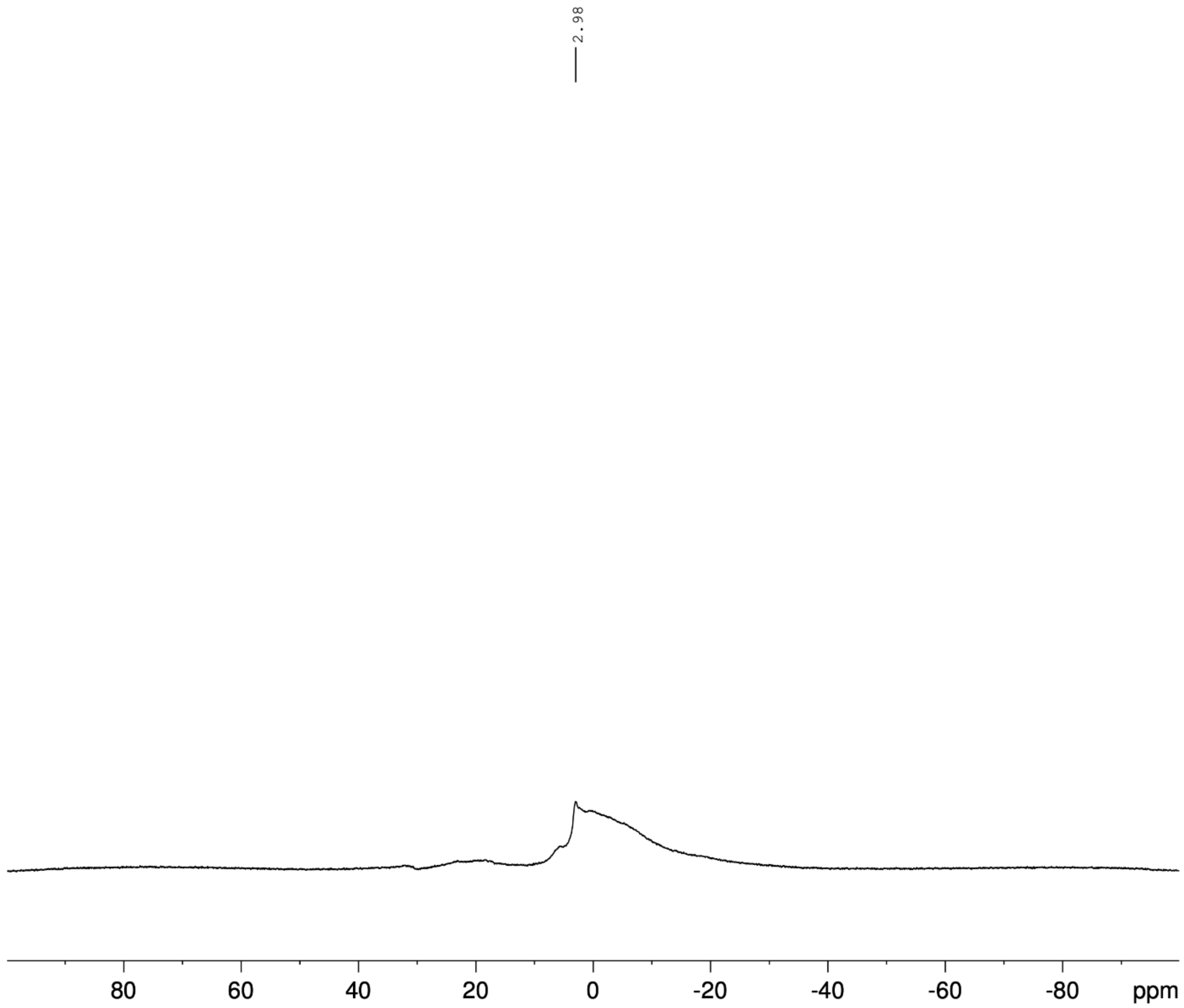


```
Current Data Parameters
NAME     14-13082020-DR
EXPNO                12
PROCNO                1

F2 - Acquisition Parameters
Date_          20200813
Time              12.34 h
INSTRUM           spect
PROBHD   Z126545_0027 (
PULPROG            zgbs
TD                16384
SOLVENT             Tol
NS                 3072
DS                    4
SWH           38461.539 Hz
FIDRES         4.695012 Hz
AQ            0.2129920 sec
RG                189.6
DW               13.000 usec
DE                18.00 usec
TE                298.0 K
D1           0.10000000 sec
TD0                   1
SFO1        192.5455532 MHz
NUC1                11B
P1                19.80 usec
P2                39.60 usec
PLW1        50.00000000 W

F2 - Processing parameters
SI                32768
SF          192.5455532 MHz
WDW                  EM
SSB      0
LB                10.00 Hz
GB       0
PC                 1.40
```

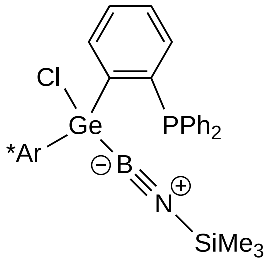

Figure S4. ^{11}B NMR (Tol-d_8) of compound **3**.

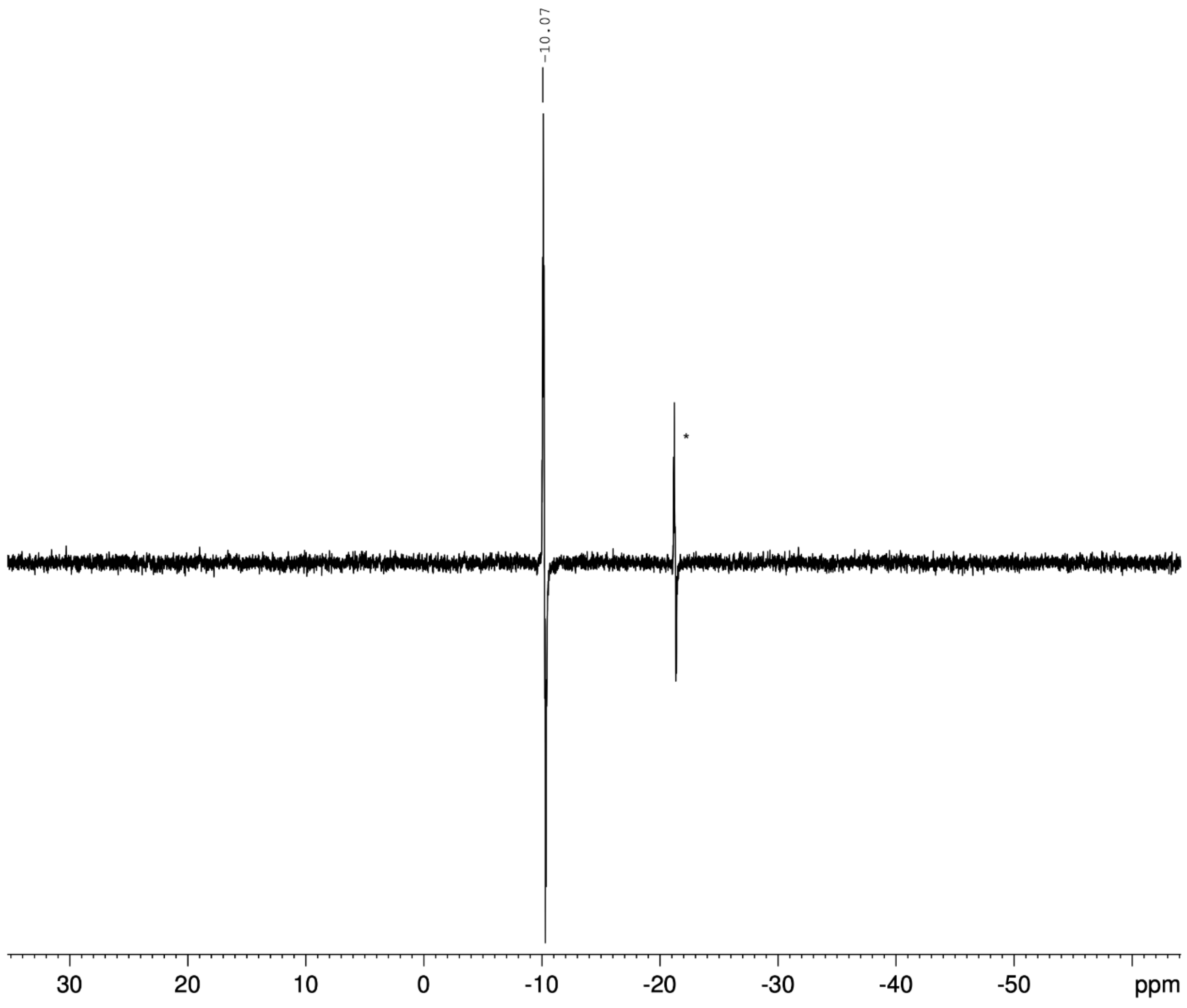


```
Current Data Parameters
NAME     25-18082020-DR
EXPNO                15
PROCNO                1

F2 - Acquisition Parameters
Date_          20200818
Time              15.36 h
INSTRUM           spect
PROBHD   Z126545_0027 (
PULPROG         ineptnd
TD                16384
SOLVENT             Tol
NS                  429
DS                   16
SWH           19132.652 Hz
FIDRES         2.335529 Hz
AQ            0.4281685 sec
RG                189.6
DW               26.133 usec
DE                18.00 usec
TE                233.1 K
CNST2         6.5999999
D1           5.00000000 sec
D4           0.03787879 sec
TD0                   1
SFO1        119.2289493 MHz
NUC1               29Si
P1                14.50 usec
P2                29.00 usec
PLW1        50.00000000 W
SFO2        600.1324005 MHz
NUC2                 1H
P3                12.00 usec
P4                24.00 usec
PLW2        23.41200066 W

F2 - Processing parameters
SI                32768
SF          119.2289493 MHz
WDW                  EM
SSB      0
LB                 1.00 Hz
GB       0
PC                 1.40
```

* grease

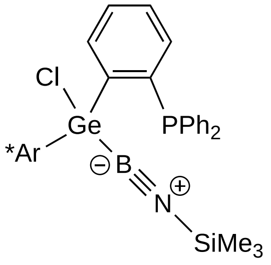

Figure S5. ^{29}Si INEPT NMR (Tol-d_8) of compound **3**.

NMR spectra of the reaction mixture of **3’**

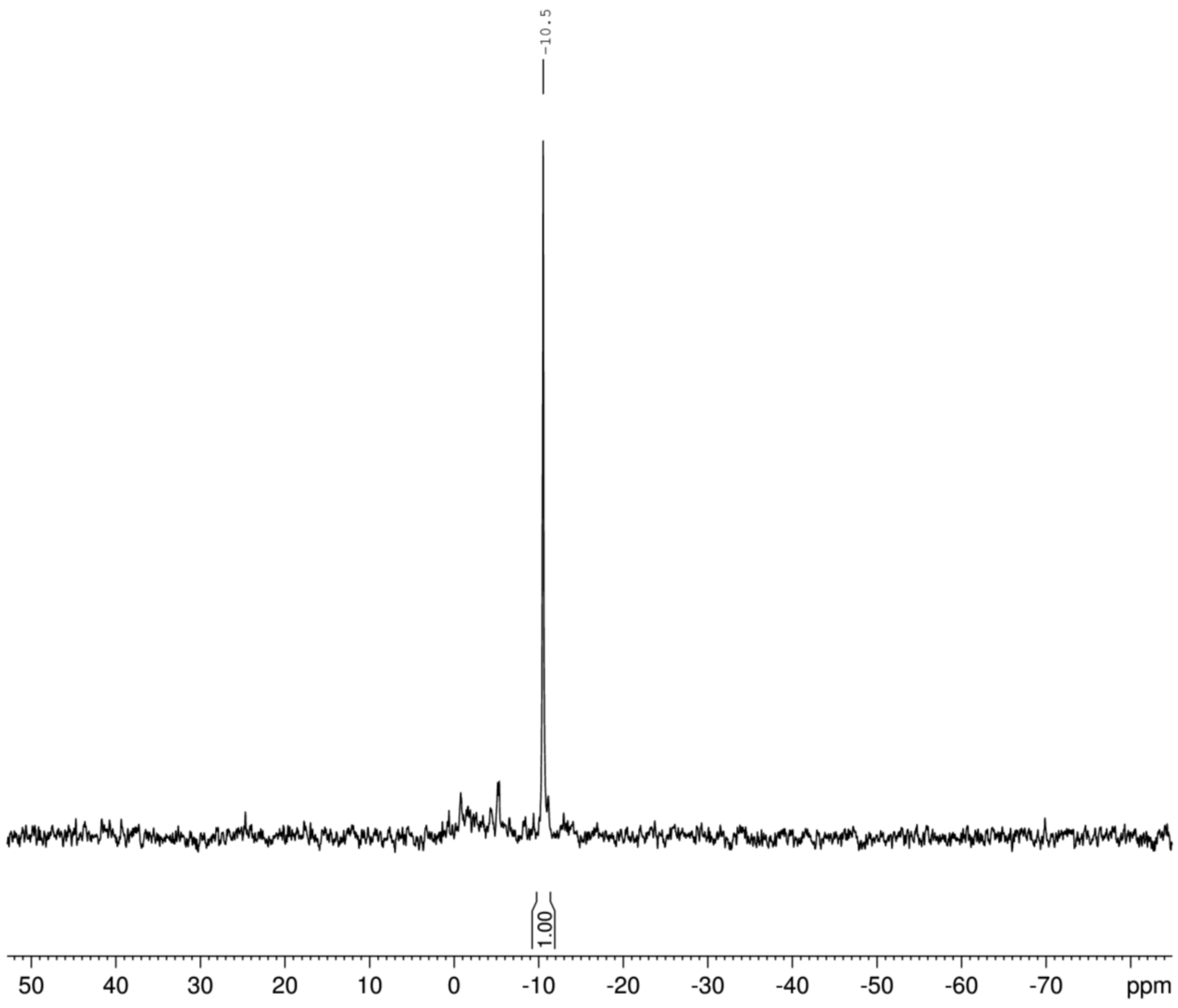


```
Current Data Parameters
NAME            DR420-300
EXPNO                  11
PROCNO                  1

F2 - Acquisition Parameters
Date_            20200814
Time                18.44 h
INSTRUM             spect
PROBHD    Z104275_0338 (
PULPROG           zgig30
TD                 78426
SOLVENT             C6D6
NS                   256
DS                     0
SWH            73529.414 Hz
FIDRES          1.875128 Hz
AQ             0.5332968 sec
RG                204.67
DW                 6.800 usec
DE                  6.50 usec
TE                 298.0 K
D1            0.10000000 sec
D11           0.03000000 sec
TD0                    1
SFO1         121.4948510 MHz
NUC1                 31P
P0                  4.00 usec
P1                 12.00 usec
PLW1         11.36400032 W
SFO2         300.1314106 MHz
NUC2                  1H
CPDPRG[2         waltz16
PCPD2              90.00 usec
PLW2          8.26509953 W
PLW12         0.20000000 W

F2 - Processing parameters
SI                 65536
SF           121.4948510 MHz
WDW                   EM
SSB      0
LB                 10.00 Hz
GB       0
PC                  1.40
```

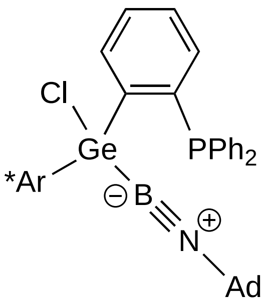

Figure S6. ^{31}P NMR (C_6D_6) of compound **3’**.

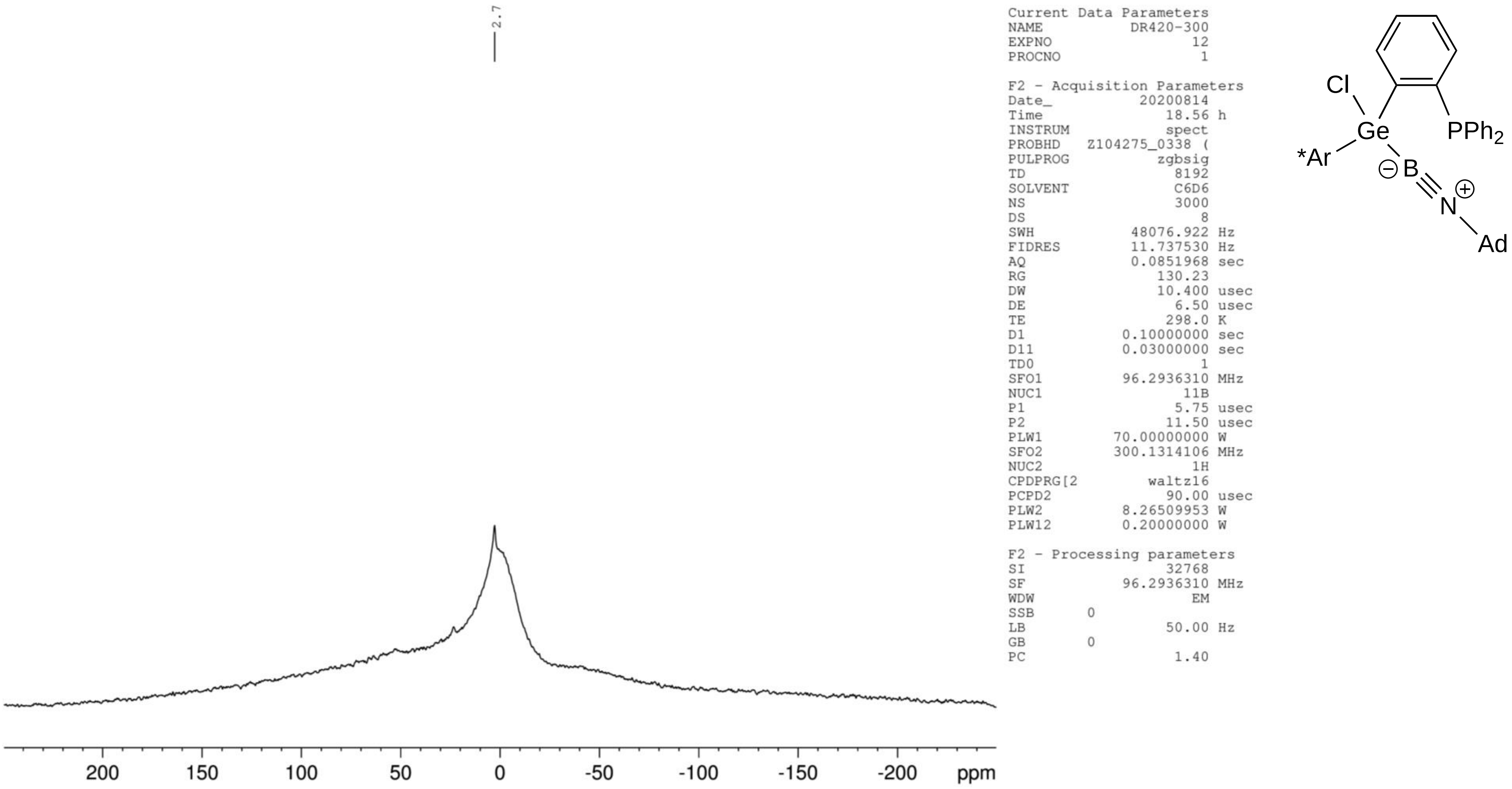

Figure S7. ^{11}B NMR (C_6D_6) of compound **3’**.

NMR spectra of compound **4**

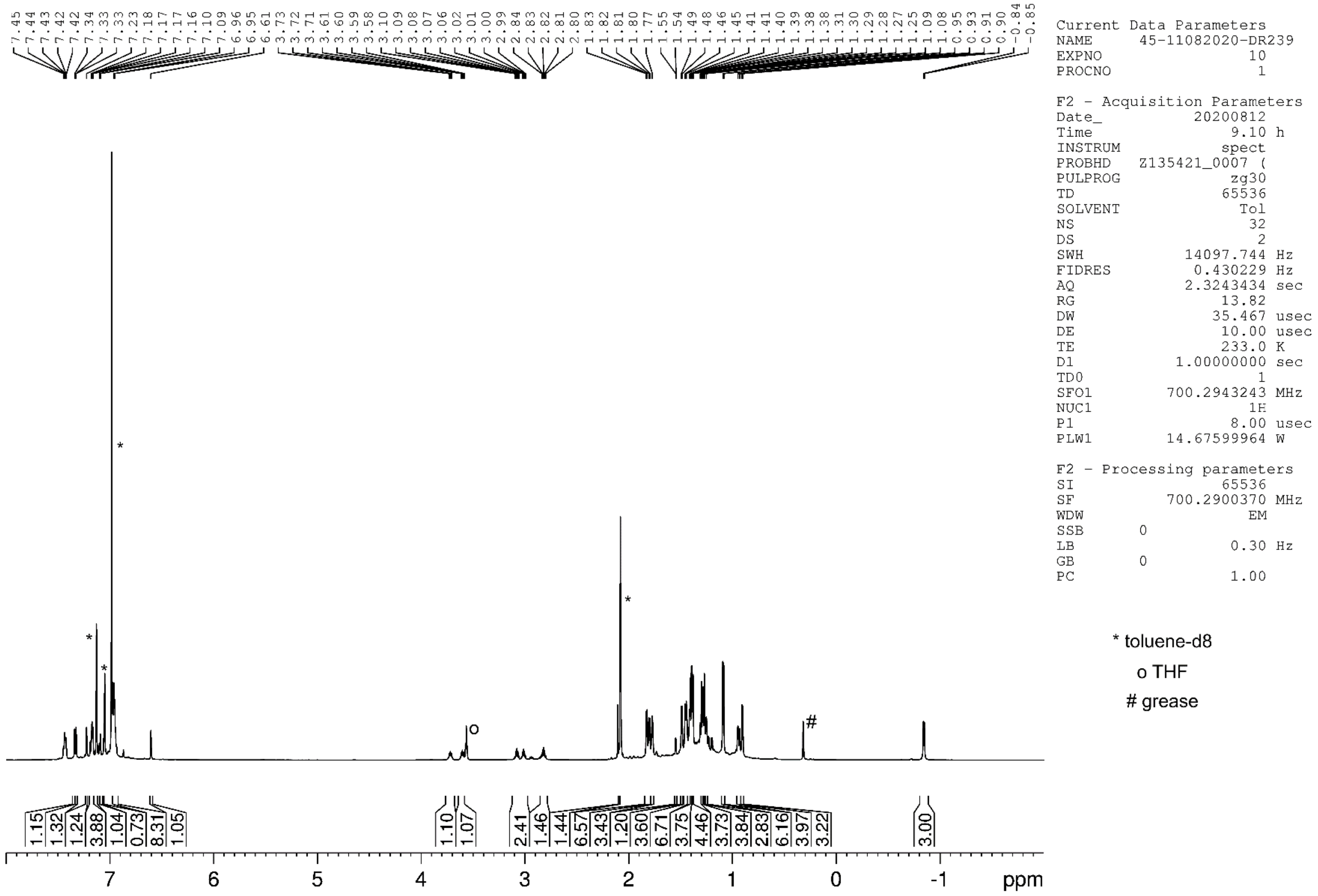

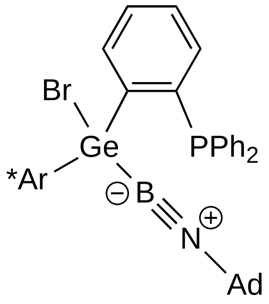

Figure S8. ^{1}H NMR (Tol-d_8) of compound **4**.

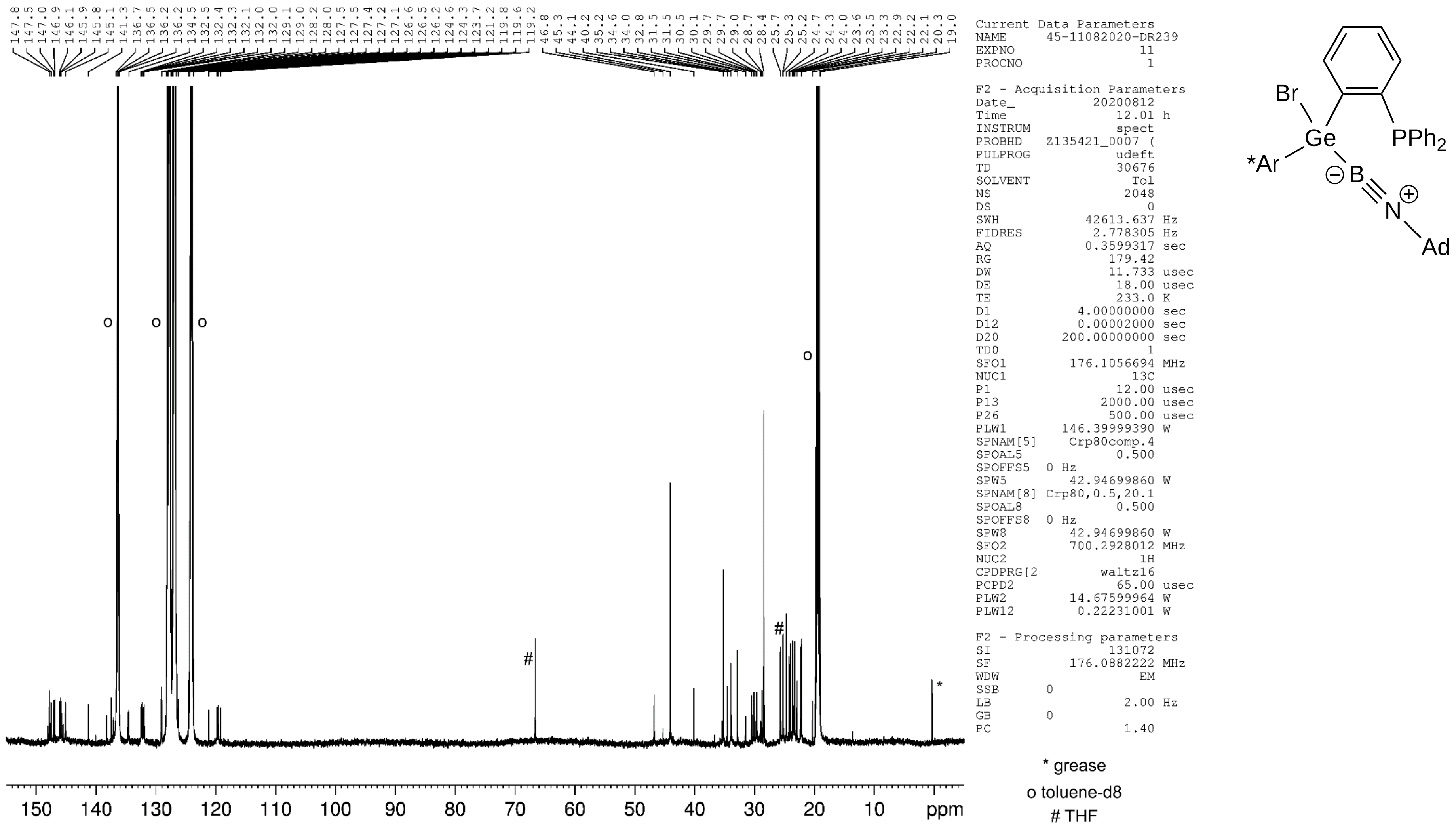

Figure S9. ^{13}C NMR (Tol-d_8) of compound **4**.

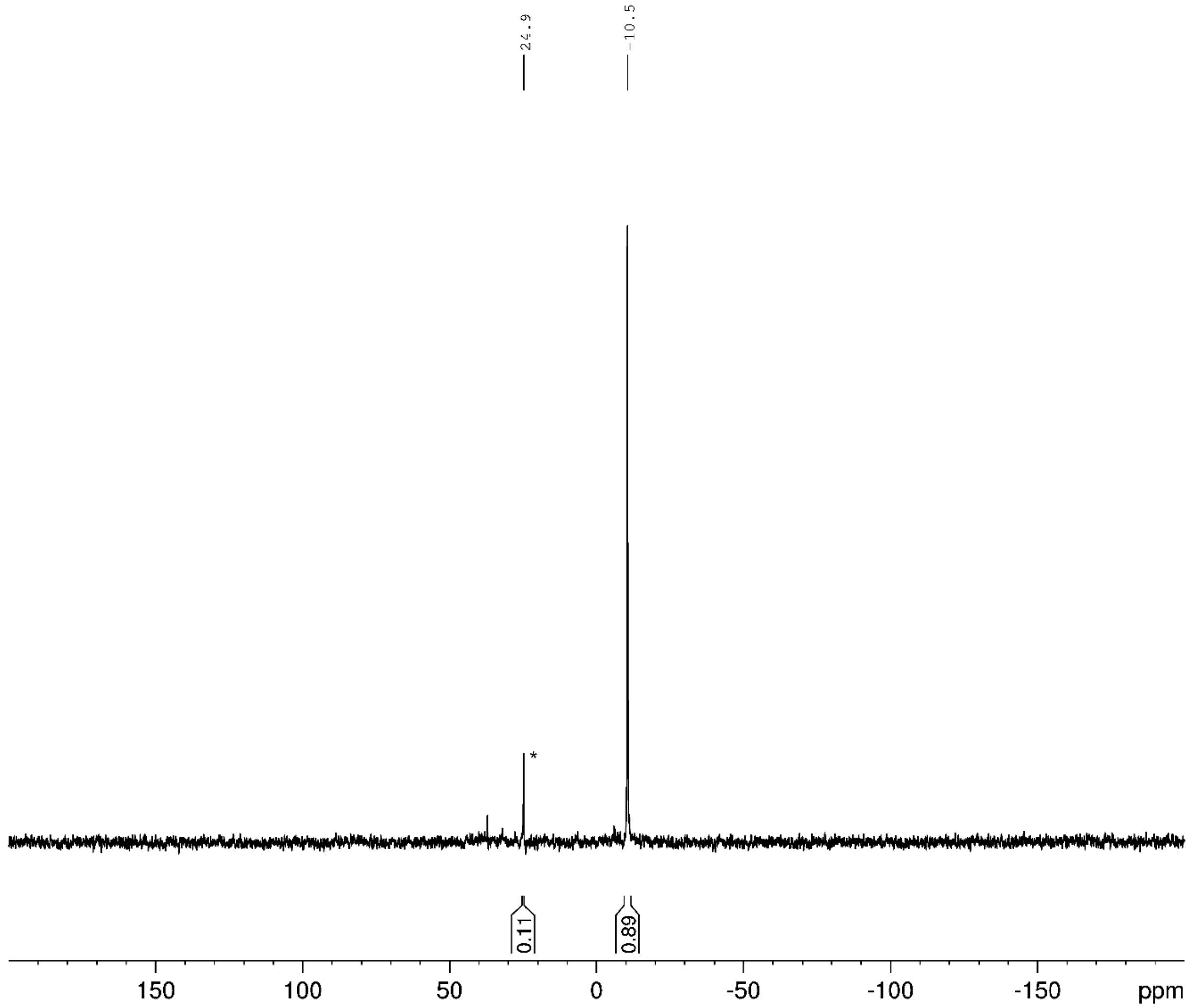


```
Current Data Parameters
NAME            DH239-300
EXPNO                  21
PROCNO                  1

F2 - Acquisition Parameters
Date_            20190729
Time                17.22 h
INSTRUM             spect
PROBHD   Z104275_0338 (
PULPROG           zgig30
TD                 78426
SOLVENT             C6D6
NS                   256
DS                     0
SWH            73529.414 Hz
FIDRES          1.875128 Hz
AQ             0.5332968 sec
RG                204.67
DW                 6.800 usec
DE                  6.50 usec
TE                 298.0 K
D1            0.10000000 sec
D11           0.03000000 sec
TD0                    1
SFO1         121.4948510 MHz
NUC1                 31P
P0                  4.00 usec
P1                 12.00 usec
PLW1         11.36400032 W
SFO2         300.1314106 MHz
NUC2                  1H
CPDPRG[2         waltz16
PCPD2              90.00 usec
PLW2          8.26509953 W
PLW12         0.20000000 W

F2 - Processing parameters
SI                 65536
SF           121.4948510 MHz
WDW                   EM
SSB      0
LB                  1.00 Hz
GB       0
PC                  1.40
```

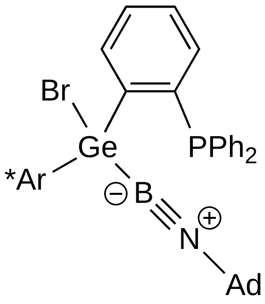

* side product 9’

Figure S10. ^{31}P NMR (C_6D_6) of compound **4**.

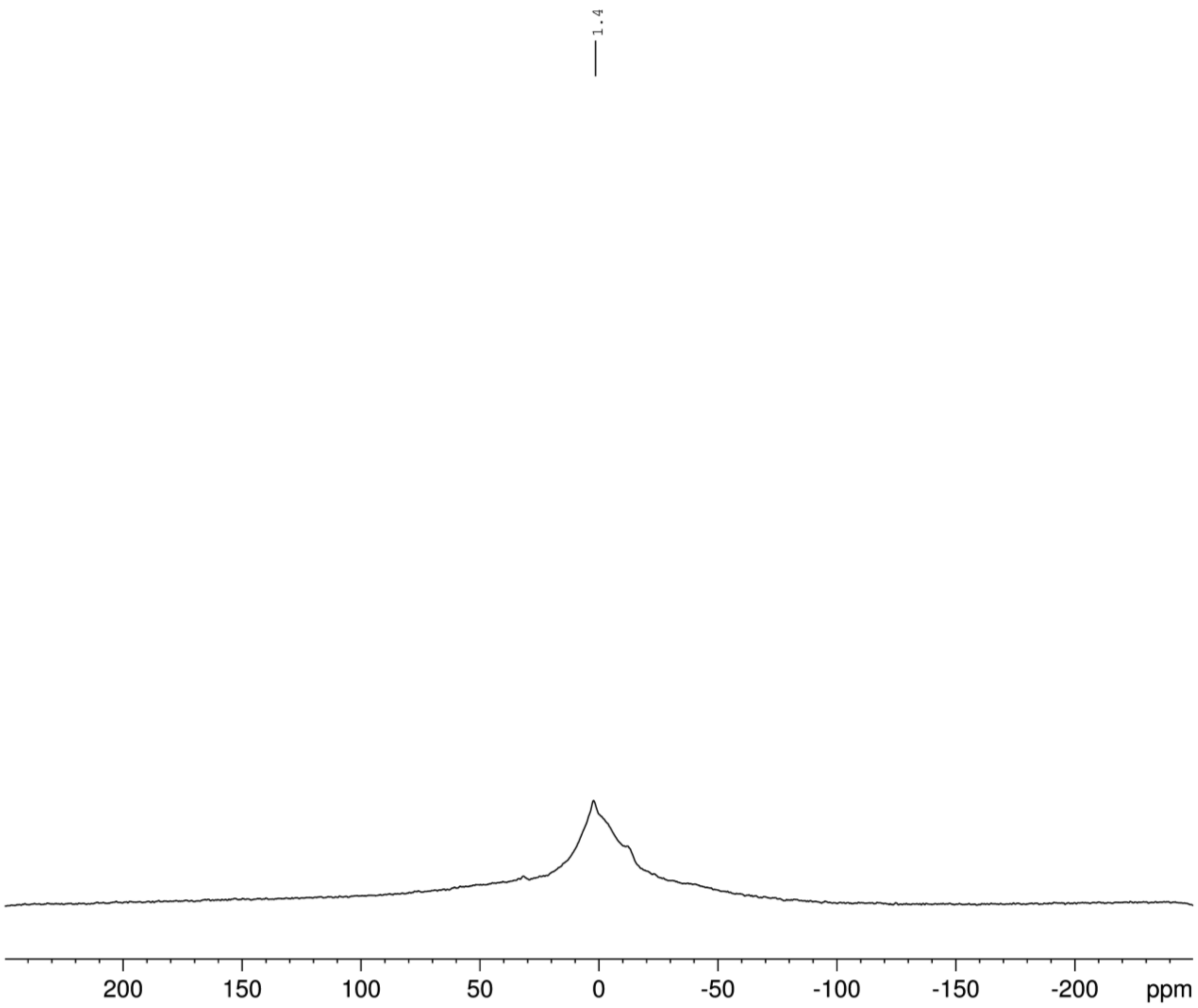


```
Current Data Parameters
NAME              DH239-300
EXPNO                    22
PROCNO                    1

F2 - Acquisition Parameters
Date_              20190729
Time                  17.37 h
INSTRUM               spect
PROBHD     Z104275_0338 (
PULPROG              zgbsig
TD                     8192
SOLVENT                C6D6
NS                     4000
DS                        8
SWH               48076.922 Hz
FIDRES            11.737530 Hz
AQ                0.0851968 sec
RG                   146.61
DW                   10.400 usec
DE                     6.50 usec
TE                    298.0 K
D1               0.10000000 sec
D11              0.03000000 sec
TD0                       1
SFO1             96.2936310 MHz
NUC1                    11B
P1                     5.75 usec
P2                    11.50 usec
PLW1            70.00000000 W
SFO2            300.1314106 MHz
NUC2                     1H
CPDPRG[2            waltz16
PCPD2                 90.00 usec
PLW2             8.26509953 W
PLW12            0.20000000 W

F2 - Processing parameters
SI                    32768
SF               96.2936310 MHz
WDW                      EM
SSB      0
LB                    50.00 Hz
GB       0
PC                     1.40
```

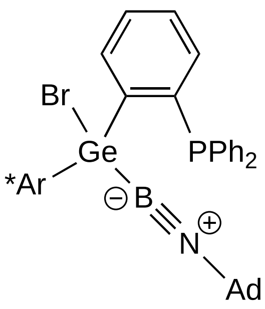

Figure S11. ^{11}B NMR (C_6D_6) of compound **4**.

NMR spectra of the reaction mixture of **4'**

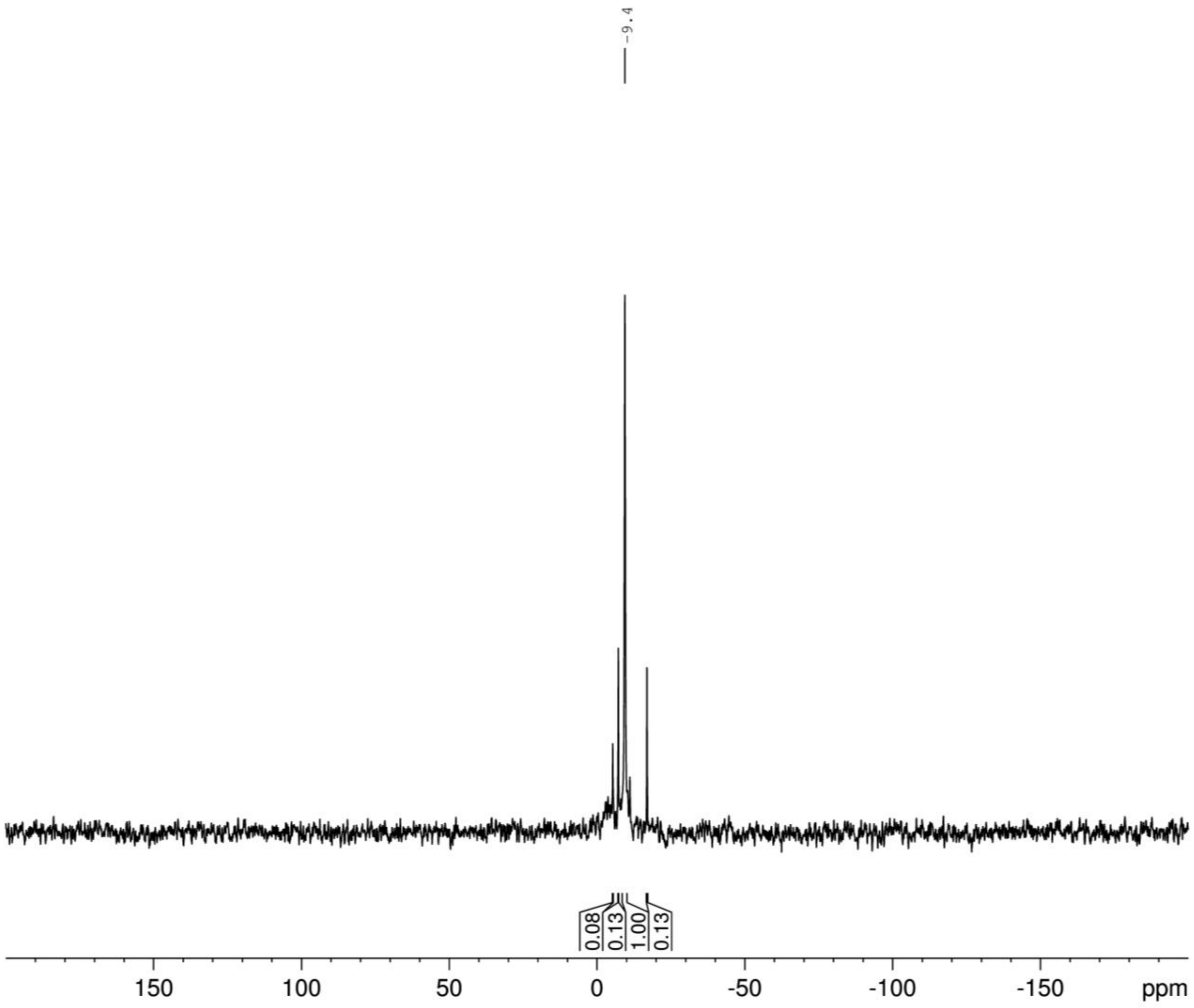


```
Current Data Parameters
NAME          DR416-300
EXPNO                21
PROCNO                1

F2 - Acquisition Parameters
Date_          20200814
Time              18.21 h
INSTRUM           spect
PROBHD   Z104275_0338 (
PULPROG          zgig30
TD                78426
SOLVENT            C6D6
NS                  256
DS                    0
SWH           73529.414 Hz
FIDRES         1.875128 Hz
AQ            0.5332968 sec
RG               204.67
DW                6.800 usec
DE                 6.50 usec
TE                298.0 K
D1           0.10000000 sec
D11          0.03000000 sec
TD0                   1
SFO1        121.4948510 MHz
NUC1                31P
P0                 4.00 usec
P1                12.00 usec
PLW1        11.36400032 W
SFO2        300.1314106 MHz
NUC2                 1H
CPDPRG[2        waltz16
PCPD2             90.00 usec
PLW2         8.26509953 W
PLW12        0.20000000 W

F2 - Processing parameters
SI                65536
SF          121.4948510 MHz
WDW                  EM
SSB      0
LB                20.00 Hz
GB       0
PC                 1.40
```

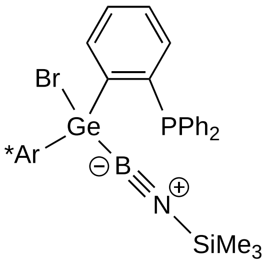

Figure S12. ^{31}P NMR (C_6D_6) of the reaction mixture of **4'**.

NMR spectra of compound **5**

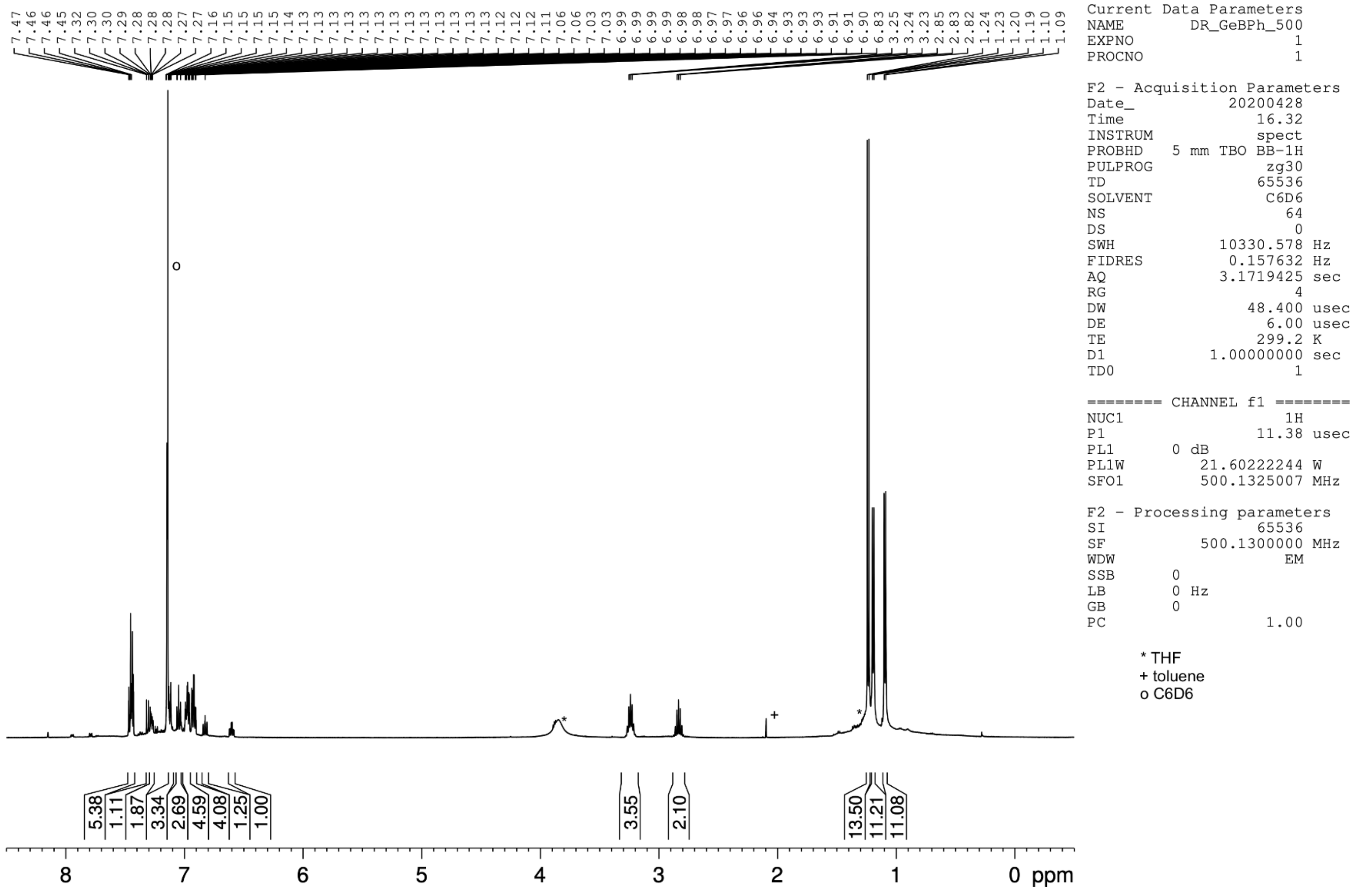

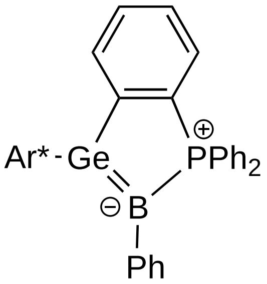

Figure S13. ^{1}H NMR (C_6D_6) of compound **5**.

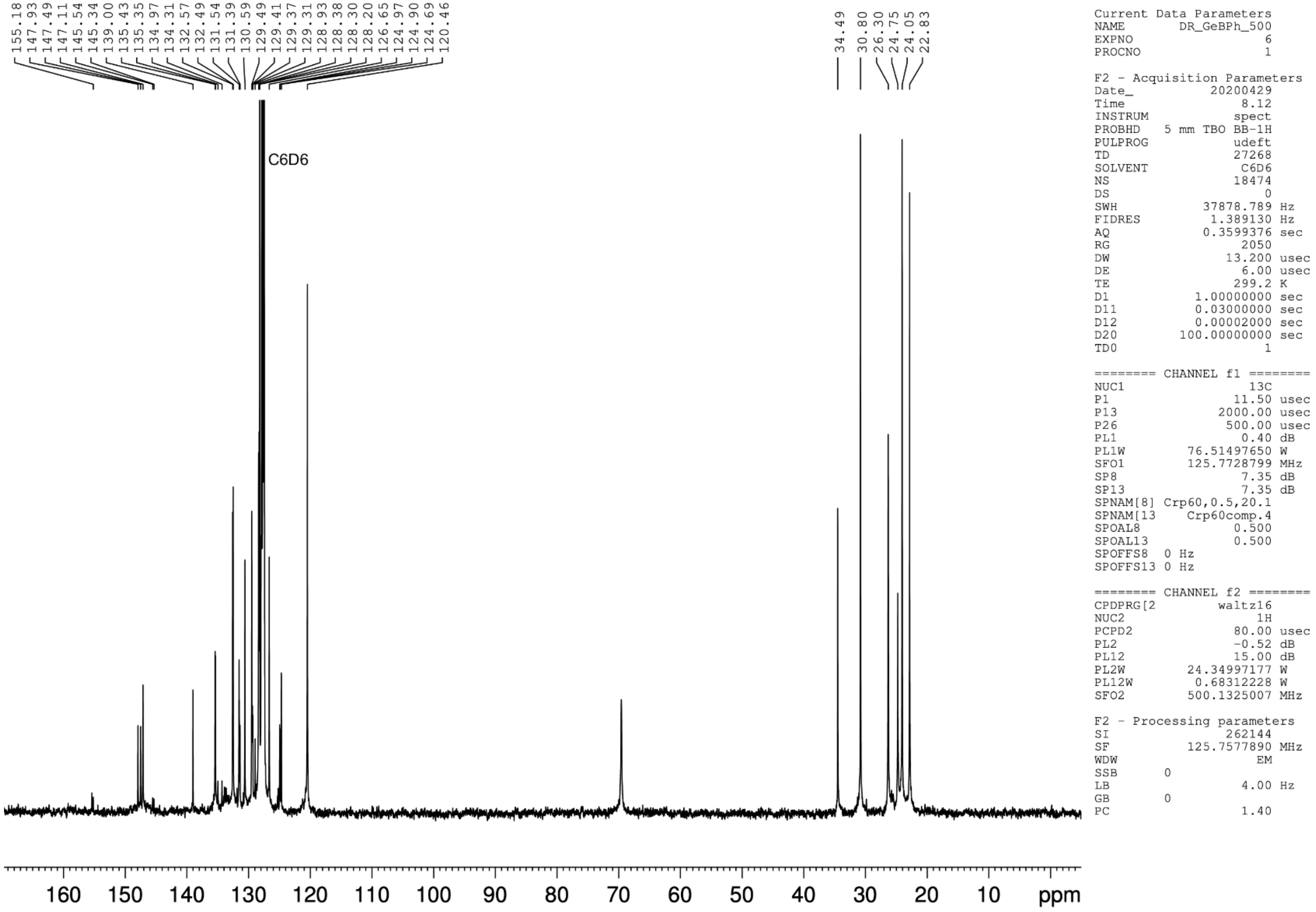

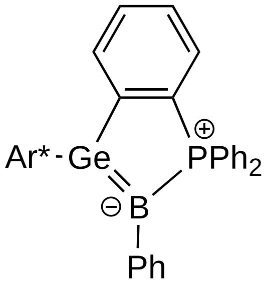

Figure S14. ^{13}C NMR (C_6D_6) of compound **5**.

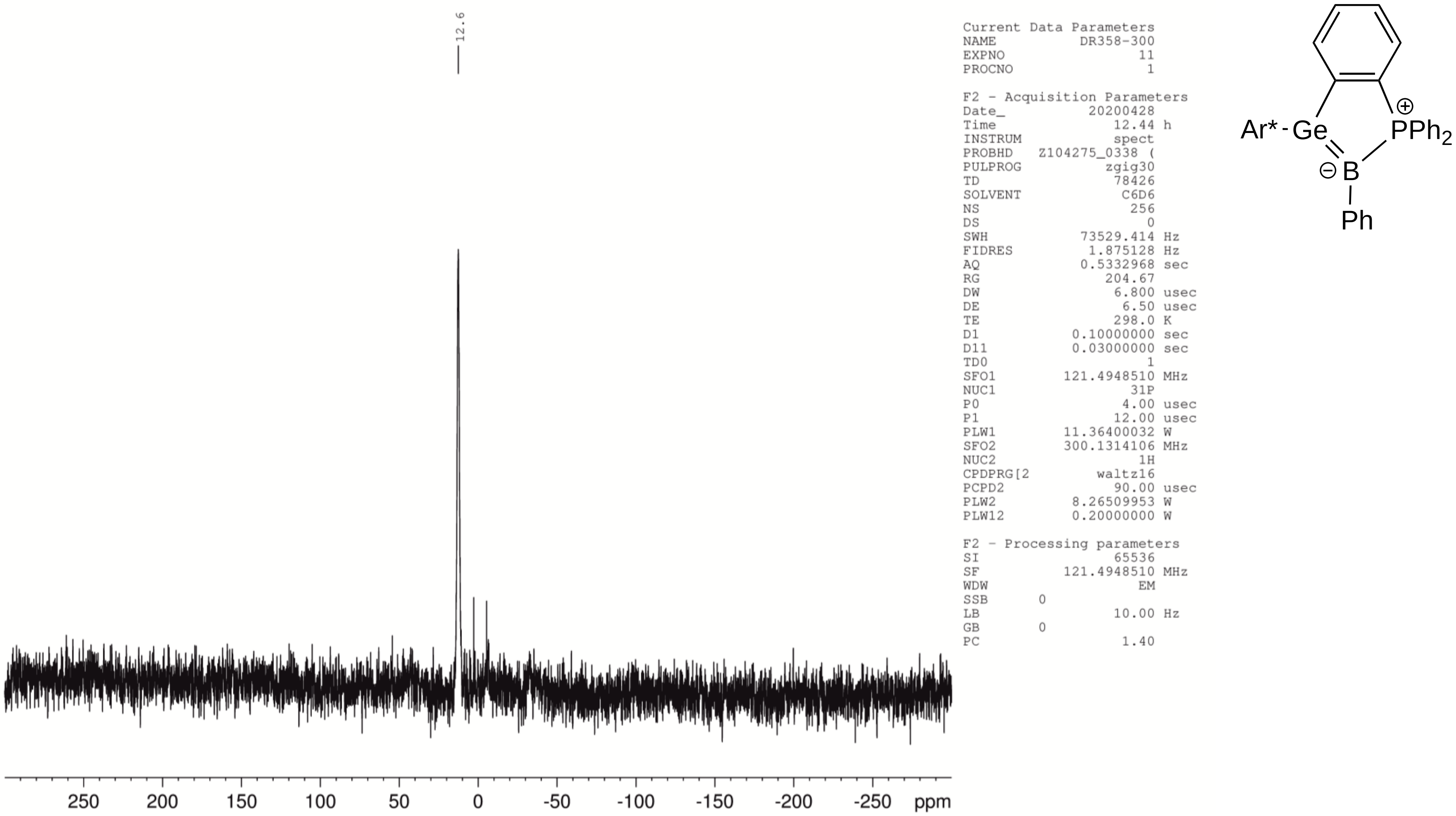

Figure S15. ^{31}P NMR (C_6D_6) of compound **5**.

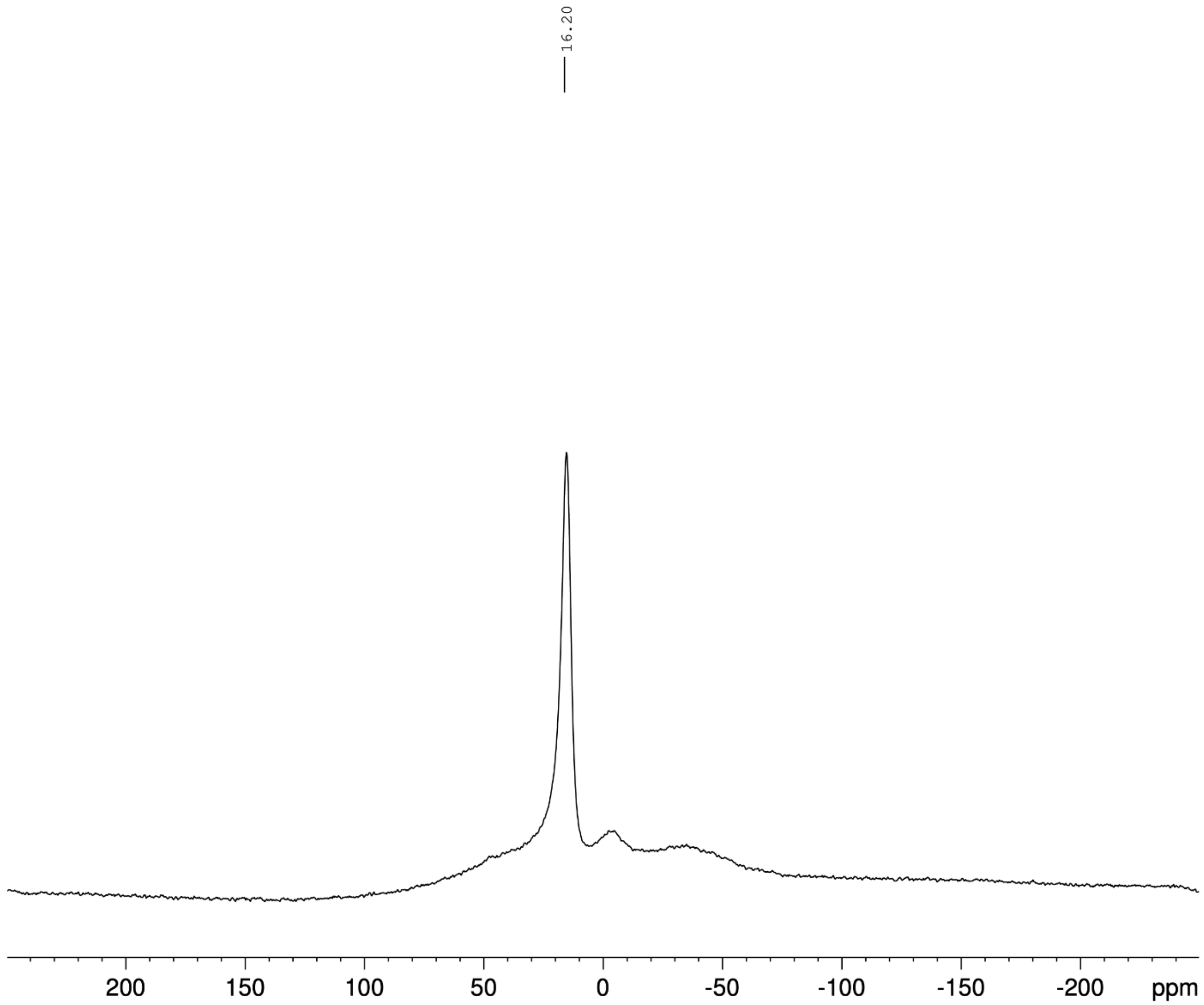

Current Data Parameters
NAME DR376-300
EXPNO 22
PROCNO 1

F2 - Acquisition Parameters
Date_ 20200513
Time 8.39 h
INSTRUM spect
PROBHD Z104275_0338 (
PULPROG zgbsig
TD 8192
SOLVENT C6D6
NS 3000
DS 8
SWH 48076.922 Hz
FIDRES 11.737530 Hz
AQ 0.0851968 sec
RG 181.04
DW 10.400 usec
DE 6.50 usec
TE 298.0 K
D1 0.10000000 sec
D11 0.03000000 sec
TD0 1
SFO1 96.2936310 MHz
NUC1 11B
P1 5.75 usec
P2 11.50 usec
PLW1 70.00000000 W
SFO2 300.1314106 MHz
NUC2 1H
CPDPRG[2 waltz16
PCPD2 90.00 usec
PLW2 8.26509953 W
PLW12 0.20000000 W

F2 - Processing parameters
SI 32768
SF 96.2936310 MHz
WDW EM
SSB 0
LB 50.00 Hz
GB 0
PC 1.40

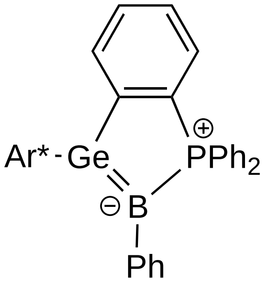

Figure S16. ^{11}B NMR (C_6D_6) of compound **5**.

NMR spectra of compound **6**

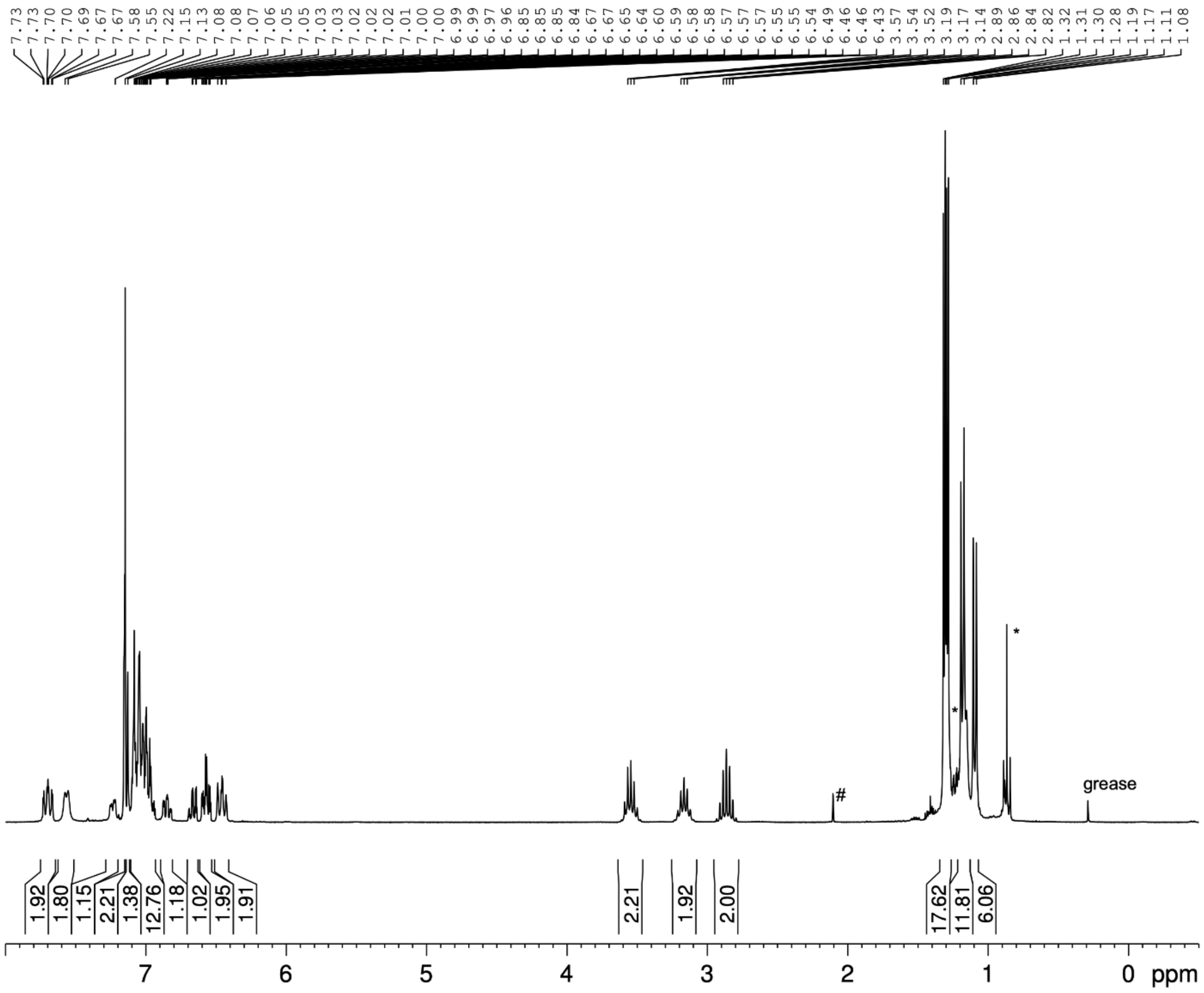


```
Current Data Parameters
NAME         DR342-300-NM
EXPNO                  10
PROCNO                  1

F2 - Acquisition Parameters
Date_            20200421
Time               12.13 h
INSTRUM             spect
PROBHD    Z104275_0338 (
PULPROG              zg30
TD                  38044
SOLVENT              C6D6
NS                     64
DS                      0
SWH              6009.615 Hz
FIDRES           0.315930 Hz
AQ              3.1652608 sec
RG                 204.67
DW                 83.200 usec
DE                   6.50 usec
TE                  298.0 K
D1             1.00000000 sec
TD0                     1
SFO1          300.1318533 MHz
NUC1                   1H
P0                   4.67 usec
P1                  14.00 usec
PLW1           8.26509953 W

F2 - Processing parameters
SI                  32768
SF            300.1300000 MHz
WDW                    EM
SSB      0
LB       0 Hz
GB       0
PC                   1.00
```

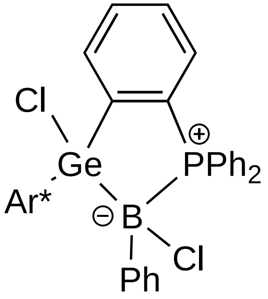

* n-hexane

# toluene

Figure S17. ^{1}H NMR (C_6D_6) of compound **6**.

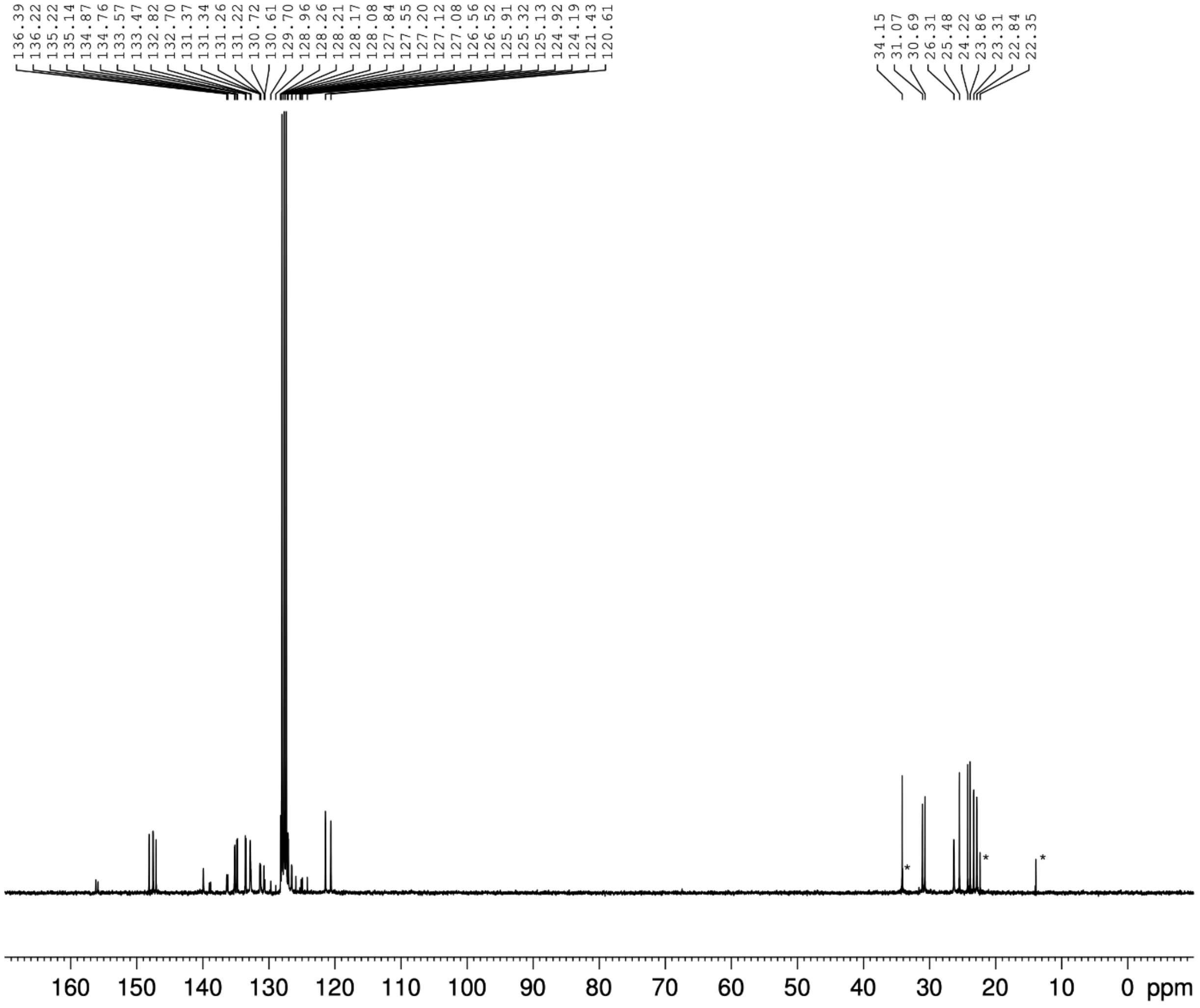


```
Current Data Parameters
NAME        DR342-300-NM
EXPNO                 13
PROCNO                 1

F2 - Acquisition Parameters
Date_           20200422
Time                1.23 h
INSTRUM            spect
PROBHD   Z104275_0338 (
PULPROG           udeft
TD                13040
SOLVENT            C6D6
NS                 4000
DS                    4
SWH           18115.941 Hz
FIDRES         2.778519 Hz
AQ            0.3599040 sec
RG               204.67
DW               27.600 usec
DE                 6.50 usec
TE                298.0 K
D1           4.00000000 sec
D12          0.00002000 sec
D20         20.00000000 sec
TD0                   1
SFO1         75.4752953 MHz
NUC1                13C
P1                10.00 usec
P13             2000.00 usec
P26              500.00 usec
PLW1        33.55099869 W
SPNAM[5]    Crp60comp.4
SPOAL5            0.500
SPOFFS5  0 Hz
SPW5         5.12620020 W
SPNAM[8] Crp60,0.5,20.1
SPOAL8            0.500
SPOFFS8  0 Hz
SPW8         5.12620020 W
SFO2       300.1312005 MHz
NUC2                 1H
CPDPRG[2        waltz16
PCPD2             90.00 usec
PLW2         8.26509953 W
PLW12        0.20000000 W

F2 - Processing parameters
SI                32768
SF           75.4677485 MHz
WDW                  EM
SSB      0
LB                 1.00 Hz
GB       0
PC                 1.40
```

* n-hexane

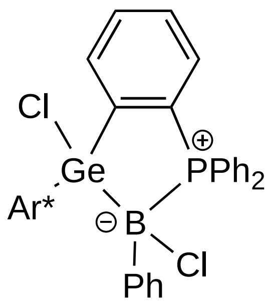

Figure S18. ^{13}C NMR (C_6D_6) of compound **6**.

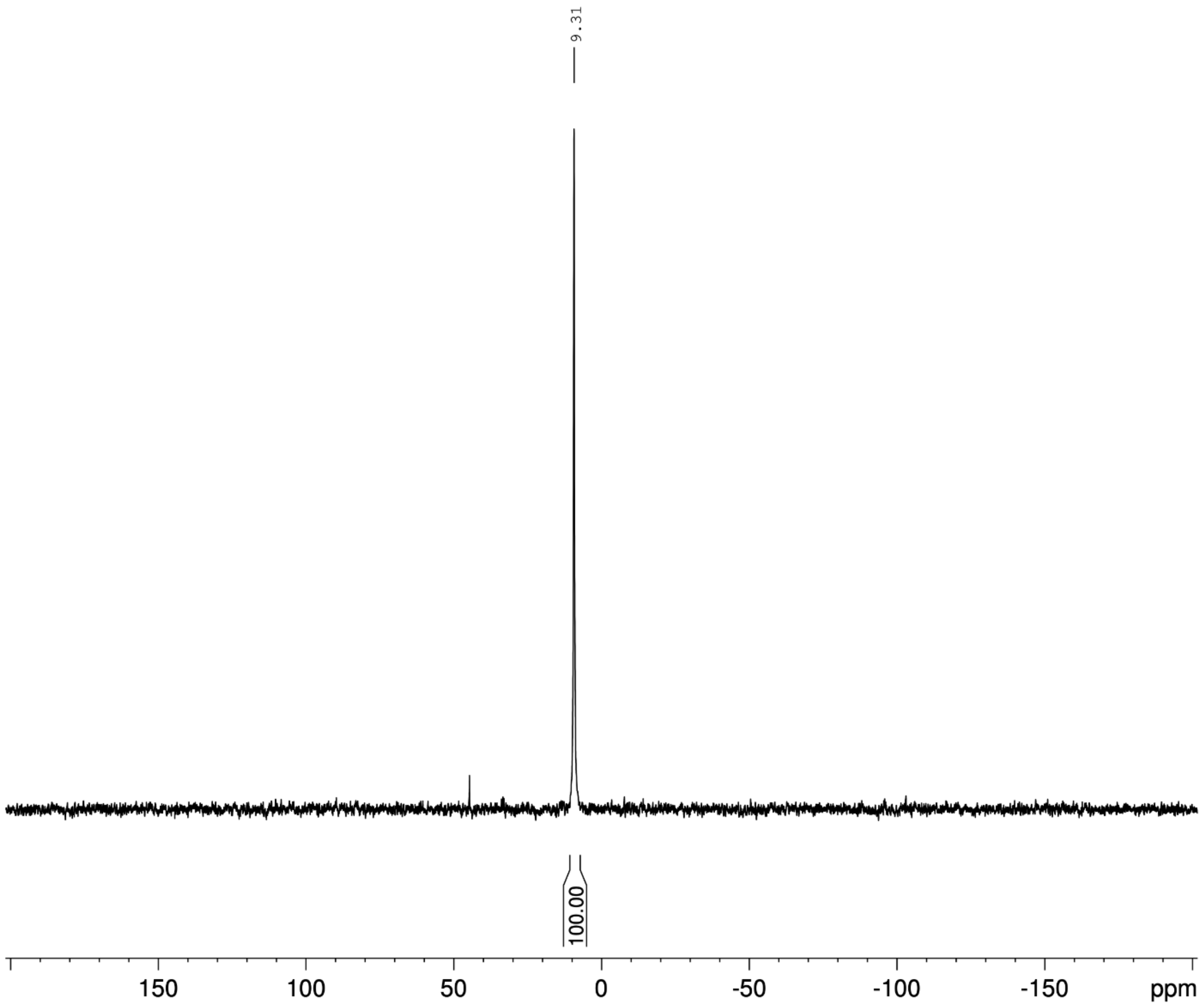


```
Current Data Parameters
NAME        DR342-300-NM
EXPNO                 11
PROCNO                 1

F2 - Acquisition Parameters
Date_           20200421
Time               12.22 h
INSTRUM            spect
PROBHD   Z104275_0338 (
PULPROG          zgig30
TD                78426
SOLVENT            C6D6
NS                  512
DS                    0
SWH           49019.609 Hz
FIDRES         1.250086 Hz
AQ            0.7999452 sec
RG               204.67
DW               10.200 usec
DE                 6.50 usec
TE                298.0 K
D1           0.10000000 sec
D11          0.03000000 sec
TD0                   1
SFO1        121.4948510 MHz
NUC1                31P
P0                 4.00 usec
P1                12.00 usec
PLW1        11.36400032 W
SFO2        300.1314106 MHz
NUC2                 1H
CPDPRG[2        waltz16
PCPD2             90.00 usec
PLW2         8.26509953 W
PLW12        0.20000000 W

F2 - Processing parameters
SI                65536
SF          121.4948510 MHz
WDW                  EM
SSB      0
LB                10.00 Hz
GB       0
PC                 1.40
```

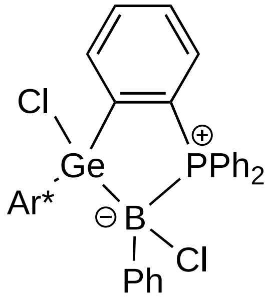

Figure S19. ^{31}P NMR (C_6D_6) of compound **6**.

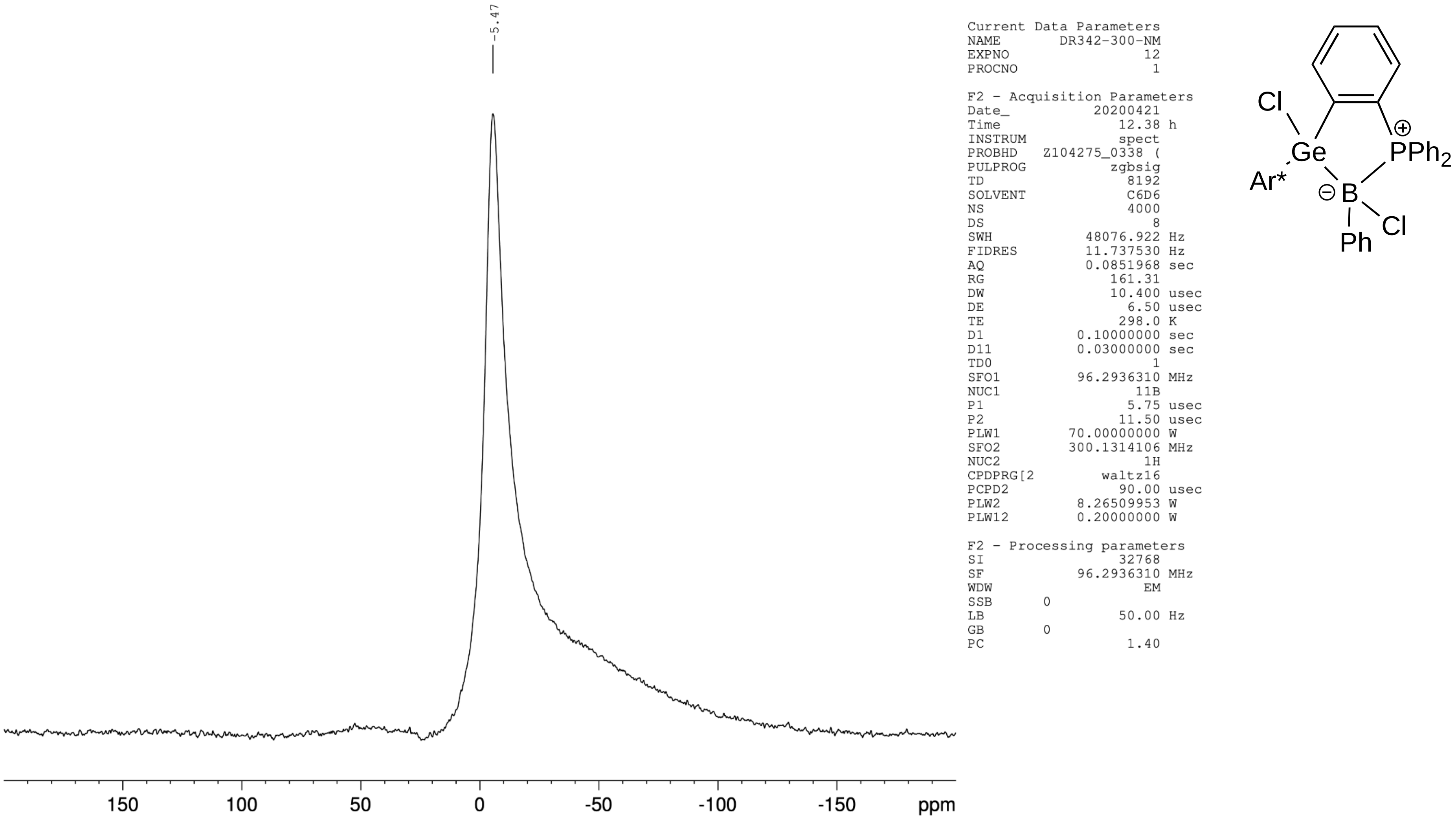

Figure S20. ^{11}B NMR (C_6D_6) of compound **6**.

NMR spectra of compound **7**

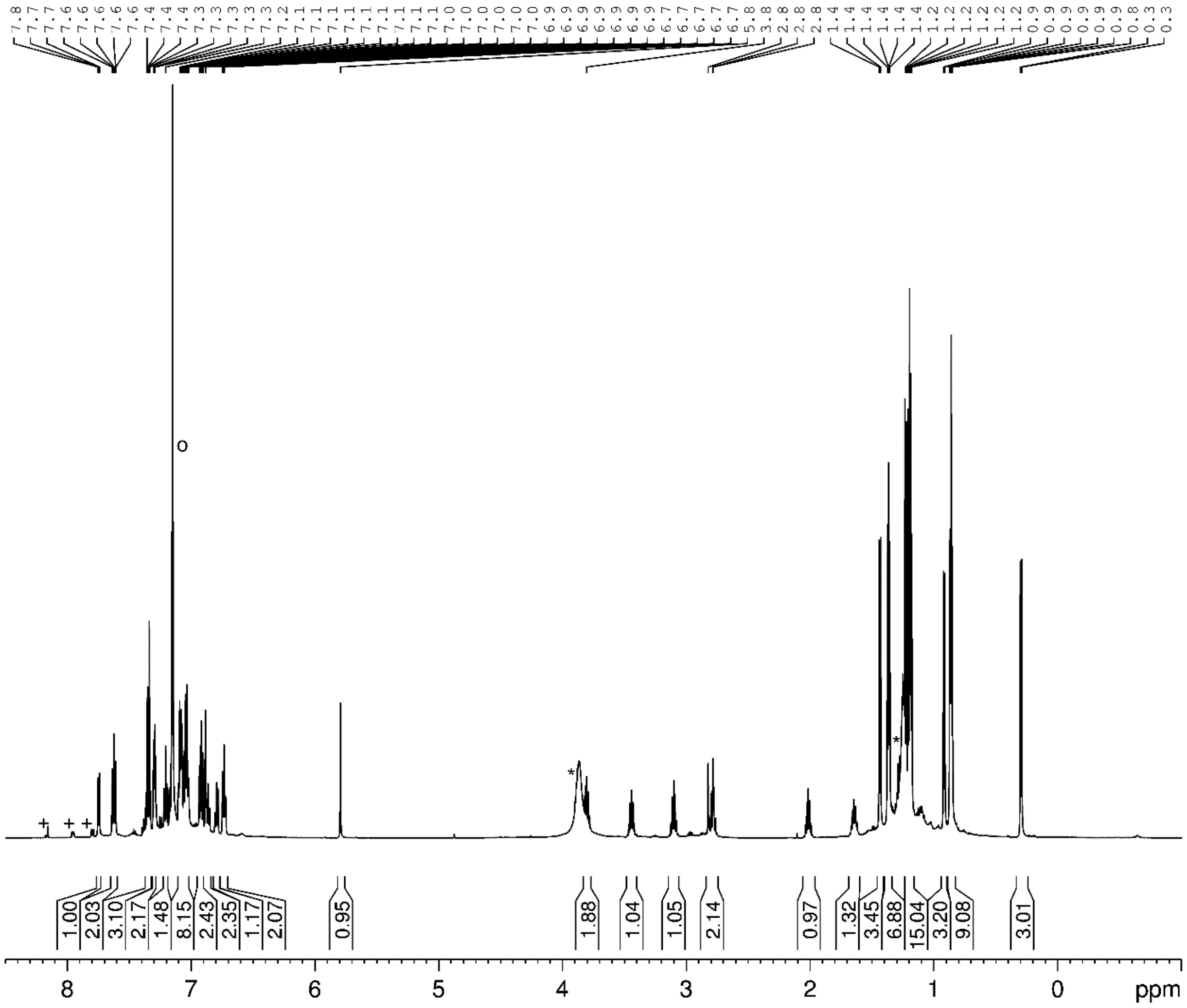


```
Current Data Parameters
NAME      48-07052020-DR
EXPNO                 10
PROCNO                 1

F2 - Acquisition Parameters
Date_           20200507
Time               15.36 h
INSTRUM            spect
PROBHD   Z126545_0027 (
PULPROG             zg30
TD                 65536
SOLVENT             C6D6
NS                    32
DS                     2
SWH            12019.230 Hz
FIDRES          0.366798 Hz
AQ             2.7262976 sec
RG                 22.25
DW                41.600 usec
DE                 10.00 usec
TE                 298.0 K
D1            1.00000000 sec
TD0                    1
SFO1         600.1337058 MHz
NUC1                  1H
P1                 12.00 usec
PLW1         23.41200066 W

F2 - Processing parameters
SI                 65536
SF           600.1300000 MHz
WDW                   EM
SSB      0
LB                  0.30 Hz
GB       0
PC                  1.00
```

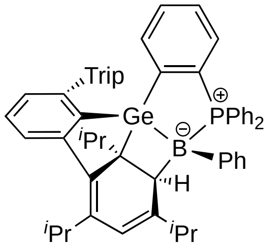

o C6D6
+ anthracene
* THF

Figure S21. ^{1}H NMR (C_6D_6) of compound **7**.

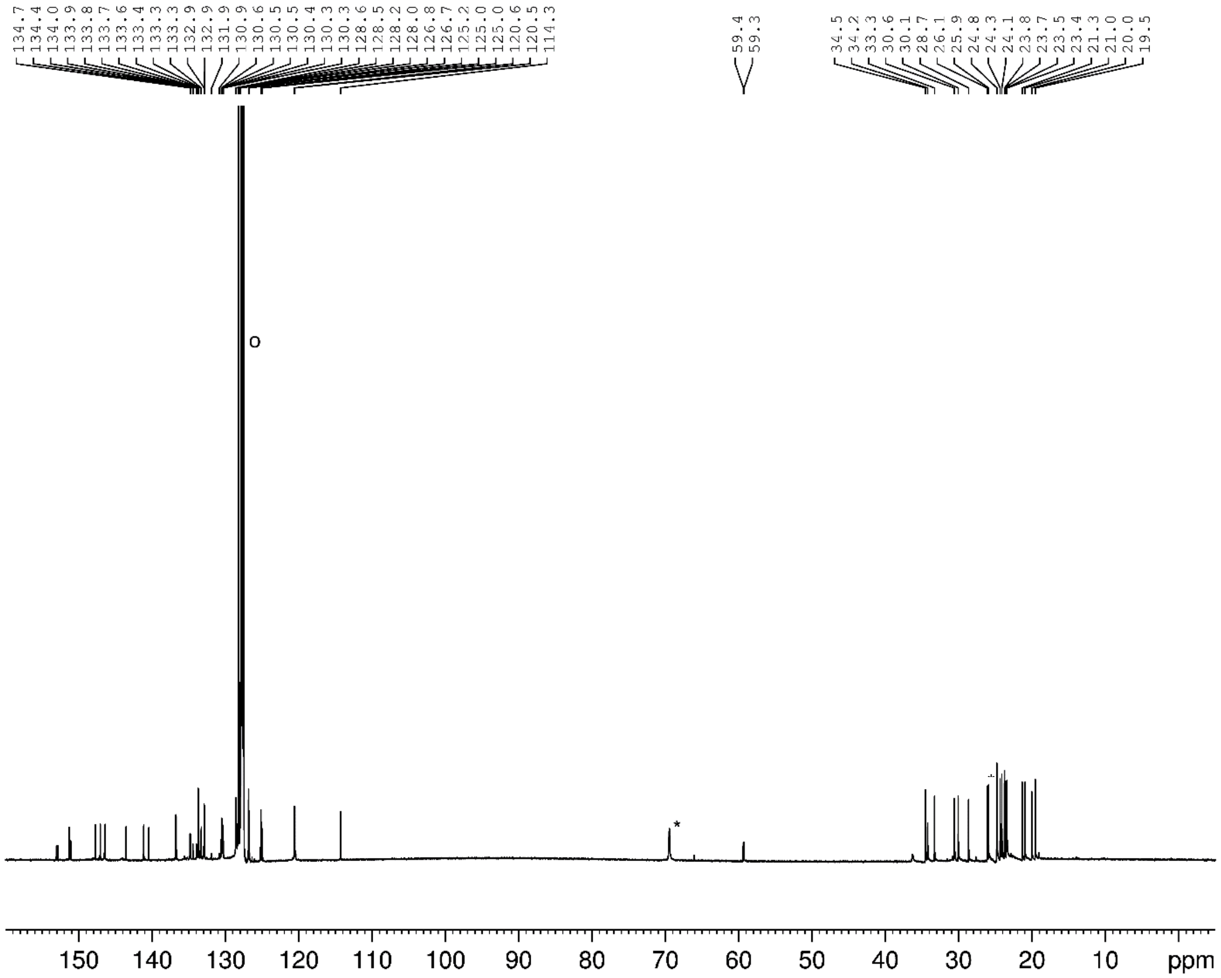


```
Current Data Parameters
NAME       48-07052020-DR
EXPNO                  11
PROCNO                  1

F2 - Acquisition Parameters
Date_            20200507
Time                17.01 h
INSTRUM             spect
PROBHD   Z126545_0027 (
PULPROG             udeft
TD                  26082
SOLVENT              C6D6
NS                   1024
DS                      8
SWH            36231.883 Hz
FIDRES          2.778306 Hz
AQ             0.3599316 sec
RG                  189.6
DW                 13.800 usec
DE                  18.00 usec
TE                  298.0 K
D1            4.00000000 sec
D12           0.00002000 sec
D20          20.00000000 sec
TD0                     1
SFO1         150.9178988 MHz
NUC1                  13C
P1                  10.00 usec
P13               2000.00 usec
P26                500.00 usec
PLW1         57.02700043 W
SPNAM[5]     Crp60comp.4
SPOAL5              0.500
SPOFFS5  0 Hz
SPW5          8.71310043 W
SPNAM[8] Crp60,0.5,20.1
SPOAL8              0.500
SPOFFS8  0 Hz
SPW8          8.71310043 W
SFO2         600.1324005 MHz
NUC2                   1H
CPDPRG[2         waltz16
PCPD2               70.00 usec
PLW2         23.41200066 W
PLW12         0.68803000 W

F2 - Processing parameters
SI                 131072
SF           150.9028085 MHz
WDW                    EM
SSB      0
LB                   2.00 Hz
GB       0
PC                   1.40
```

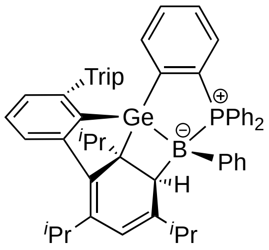

o C6D6
* THF

Figure S22. ^{13}C NMR (C_6D_6) of compound **7**.

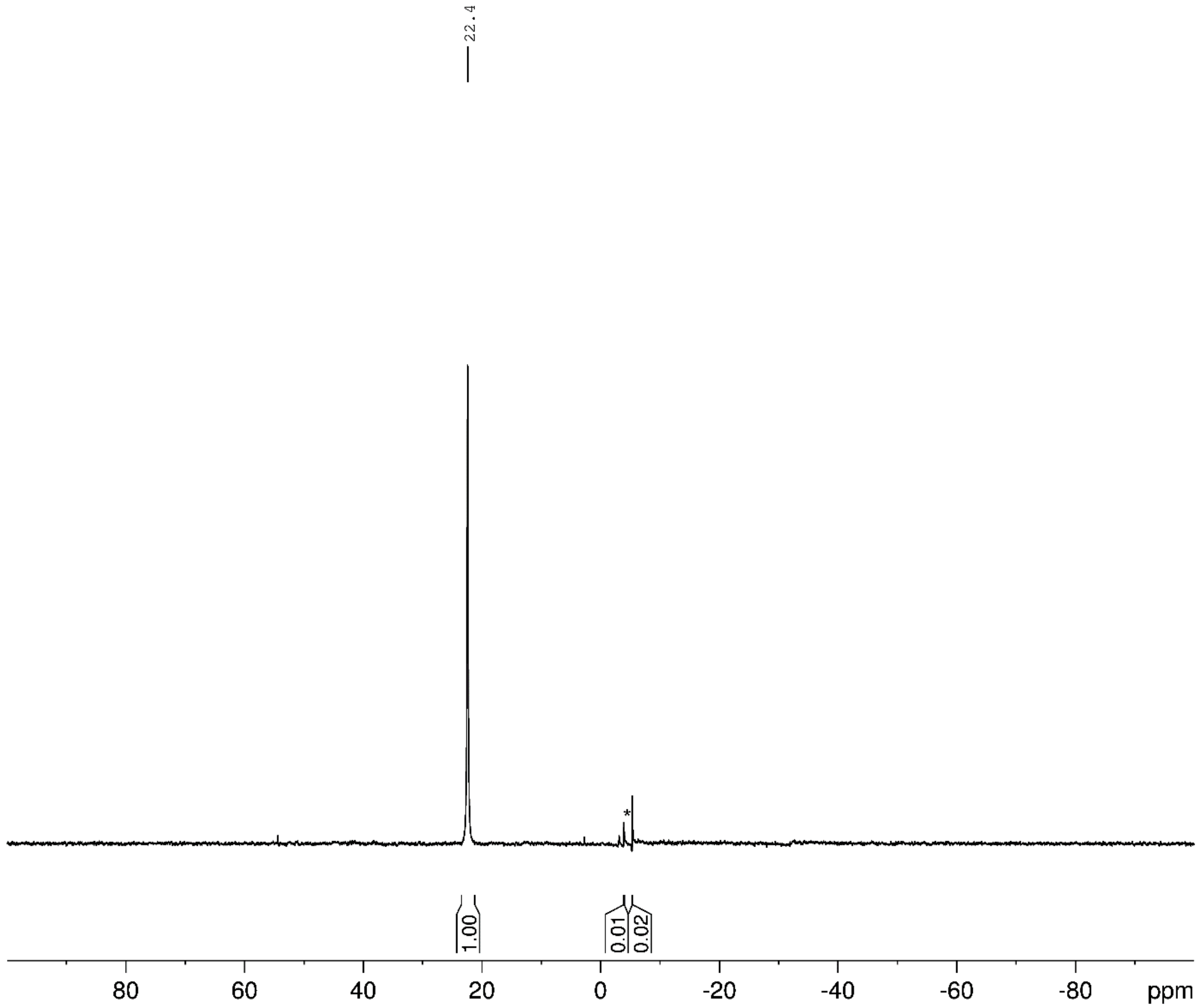


```
Current Data Parameters
NAME      48-07052020-DR
EXPNO                 13
PROCNO                 1

F2 - Acquisition Parameters
Date_           20200507
Time               17.38 h
INSTRUM            spect
PROBHD   Z126545_0027 (
PULPROG          zgpg30
TD                 65536
SOLVENT             C6D6
NS                   128
DS                     4
SWH           96153.844 Hz
FIDRES         2.934382 Hz
AQ            0.3407872 sec
RG                 189.6
DW                 5.200 usec
DE                 18.00 usec
TE                 298.0 K
D1            2.00000000 sec
D11           0.03000000 sec
TD0                    1
SFO1        242.9249301 MHz
NUC1                 31P
P1                 12.00 usec
PLW1         52.43000031 W
SFO2        600.1324005 MHz
NUC2                  1H
CPDPRG[2         waltz16
PCPD2              70.00 usec
PLW2         23.41200066 W
PLW12         0.68803000 W
PLW13         0.34606999 W

F2 - Processing parameters
SI                 32768
SF           242.9370770 MHz
WDW                   EM
SSB      0
LB                 10.00 Hz
GB       0
PC                  1.40
```

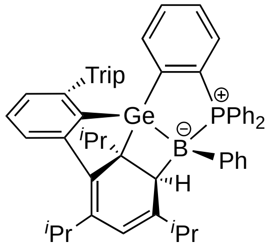

* impurities from reduction

Figure S23. ^{31}P NMR (C_6D_6) of compound **7**.

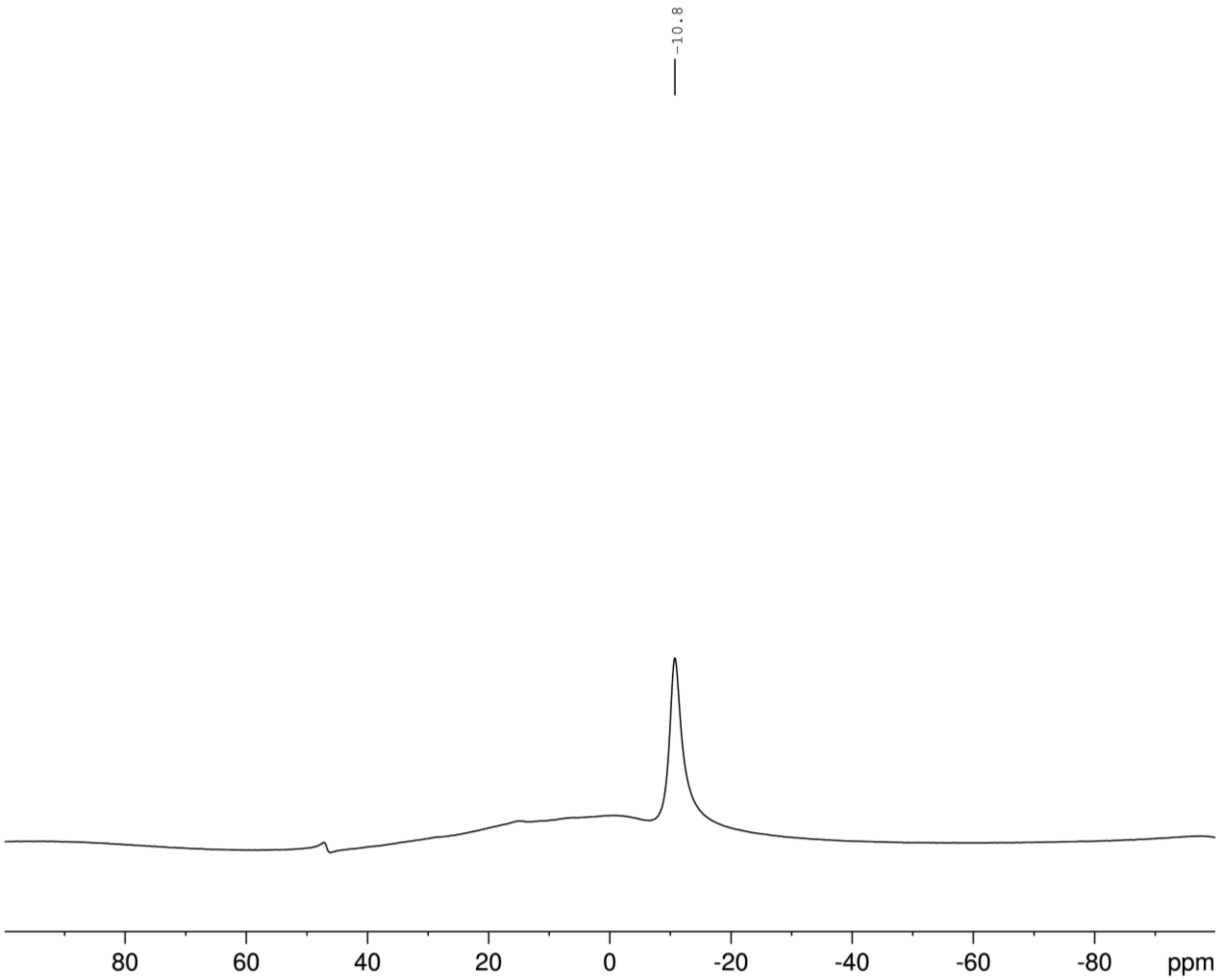


```
Current Data Parameters
NAME     48-07052020-DR
EXPNO                14
PROCNO                1

F2 - Acquisition Parameters
Date_          20200507
Time              18.10 h
INSTRUM           spect
PROBHD   Z126545_0027 (
PULPROG          zgbsig
TD                16384
SOLVENT            C6D6
NS                 5120
DS                    4
SWH           38461.539 Hz
FIDRES         4.695012 Hz
AQ            0.2129920 sec
RG                189.6
DW               13.000 usec
DE                18.00 usec
TE                298.0 K
D1           0.10000000 sec
D11          0.03000000 sec
TD0                   1
SFO1        192.5455530 MHz
NUC1                11B
P1                19.80 usec
P2                39.60 usec
PLW1        50.00000000 W
SFO2        600.1328206 MHz
NUC2                 1H
CPDPRG[2        waltz16
PCPD2             70.00 usec
PLW2        23.41200066 W
PLW12        0.68803000 W

F2 - Processing parameters
SI                16384
SF          192.5455530 MHz
WDW                  EM
SSB      0
LB                50.00 Hz
GB       0
PC                 1.40
```

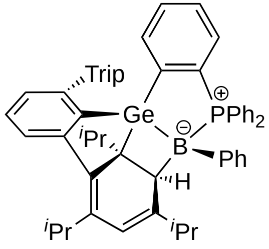

Figure S24. ^{11}B NMR (C_6D_6) of compound **7**.

NMR spectra of compound **8**

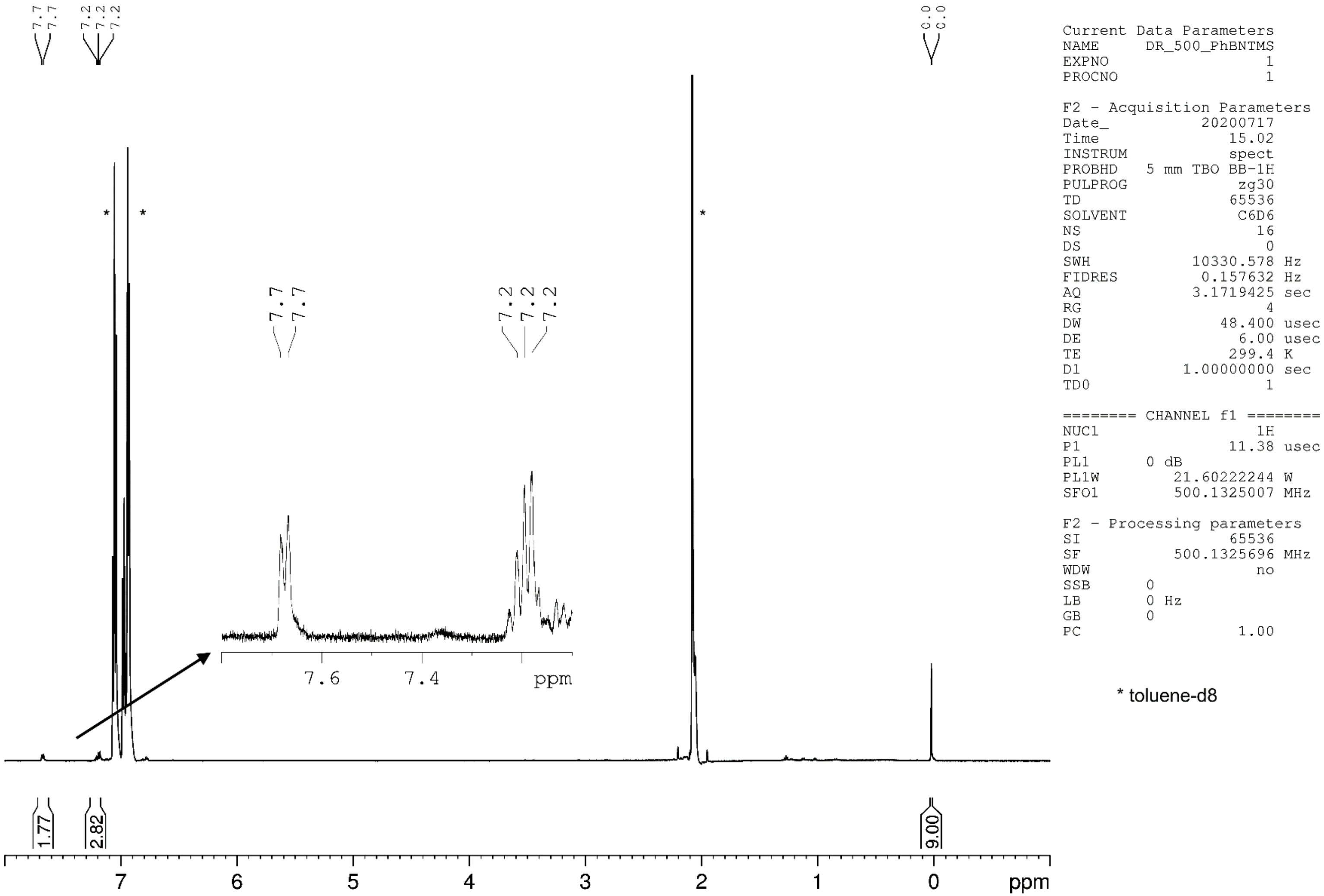

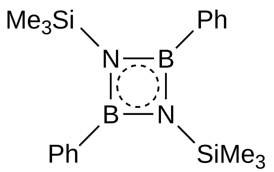

Figure S25. ^{1}H NMR (Tol-d_8) of compound **8** (dimer).

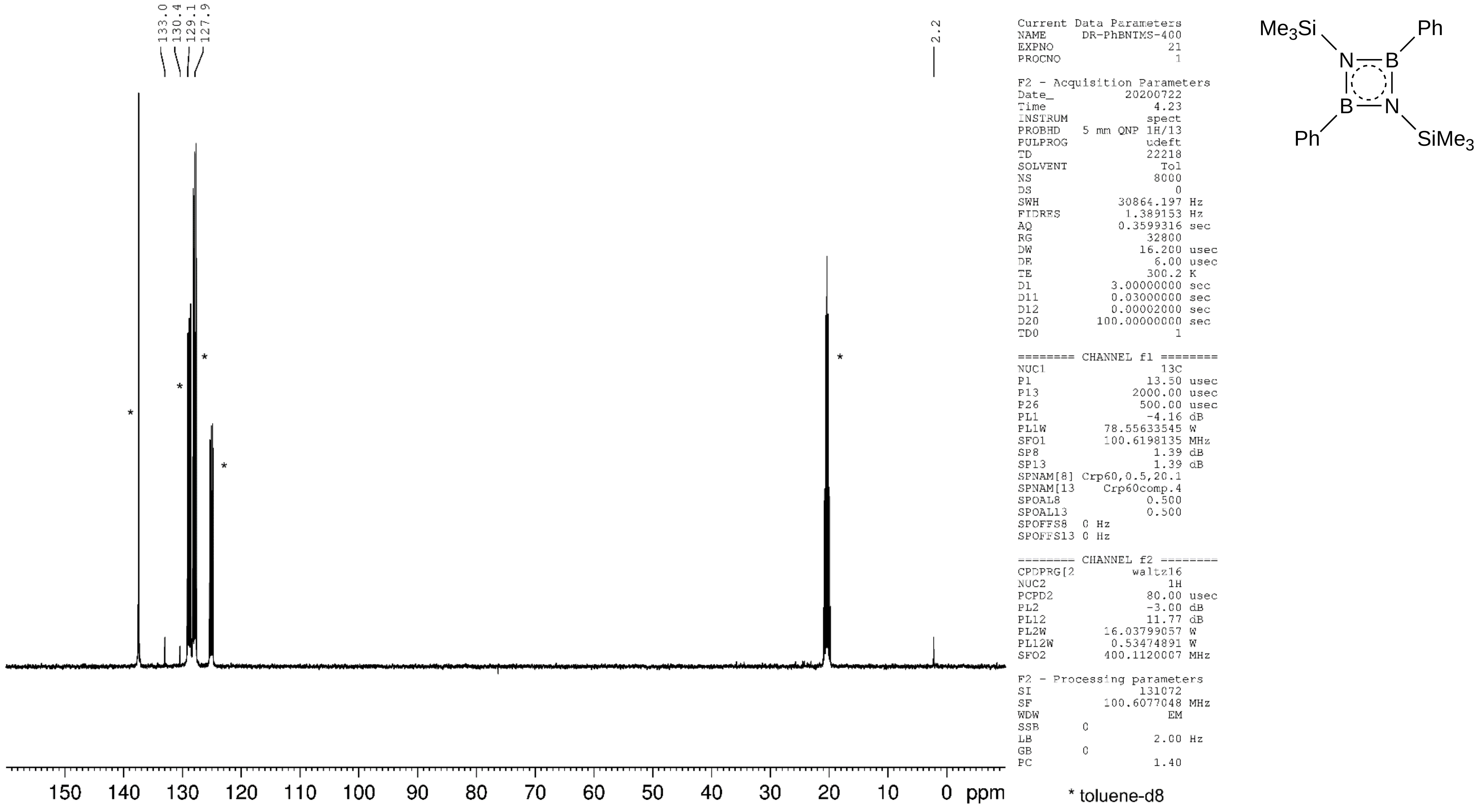

Figure S26. ^{13}C NMR (Tol-d_8) of compound **8** (dimer).

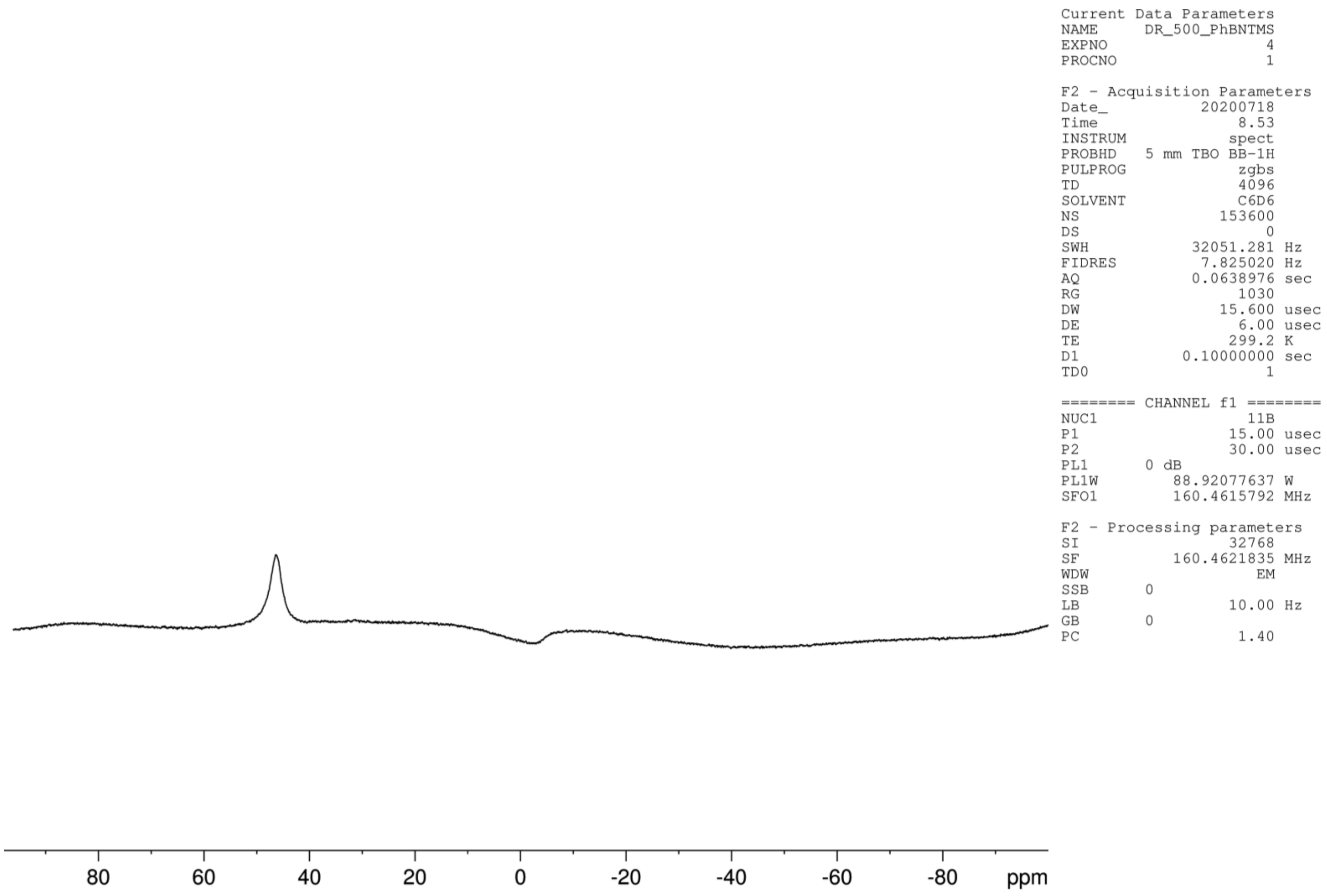

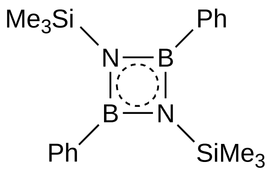

Figure S27. ^{11}B NMR (C_6D_6) of compound **8** (dimer).

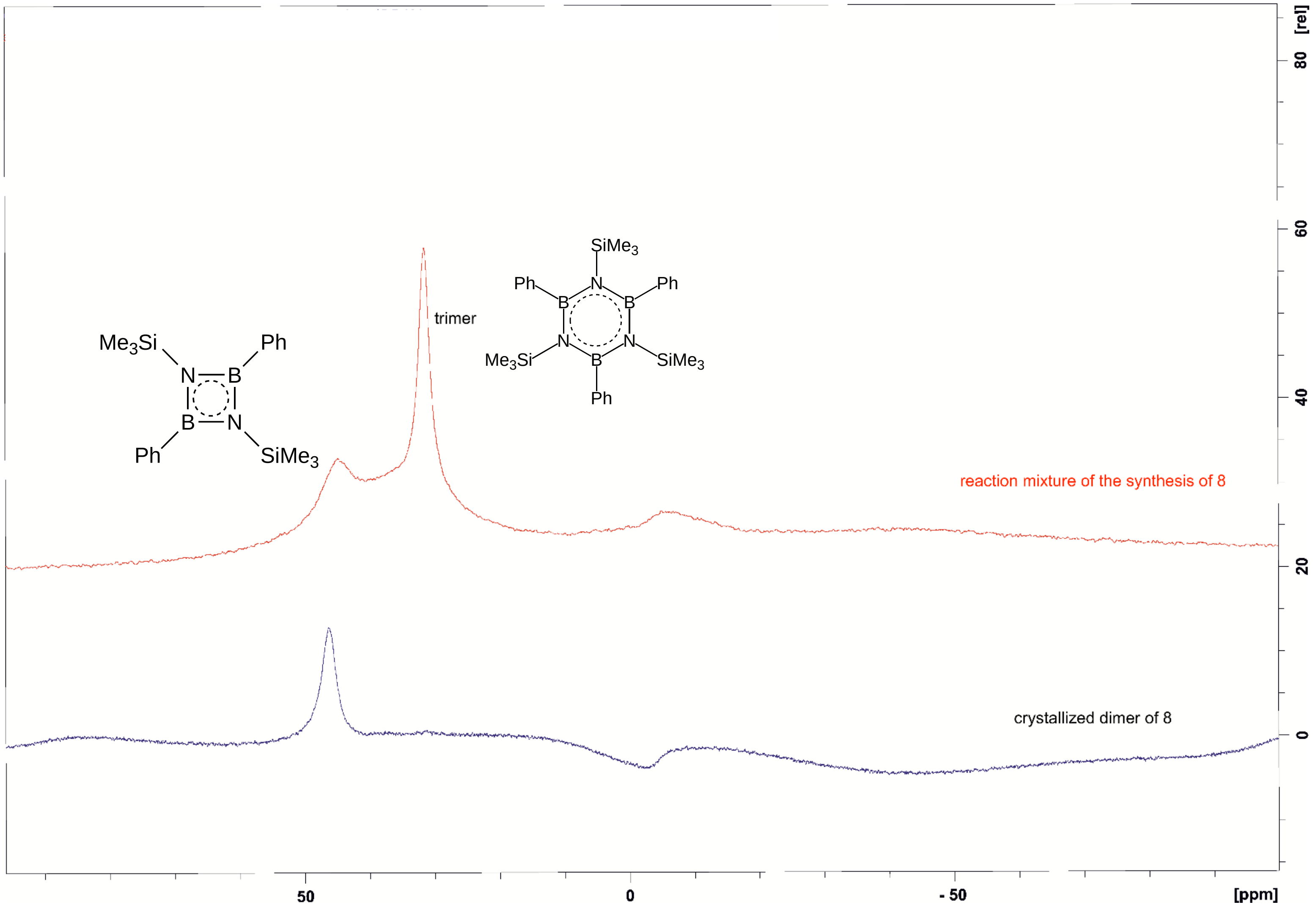

Figure S28. ^{11}B NMR (C_6D_6) of compound **8** (dimer, black) and the reaction mixture (dimer and trimer, red).

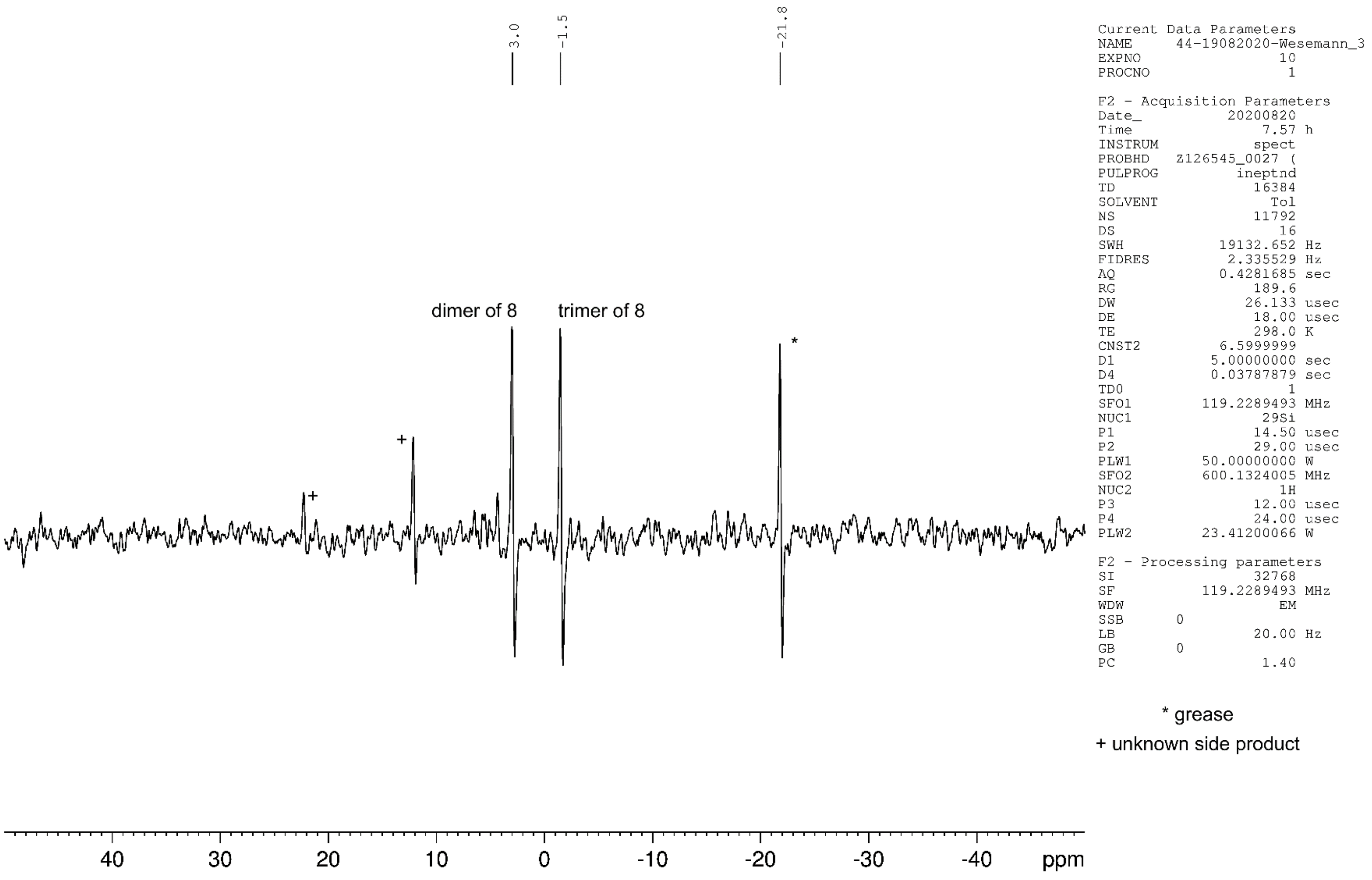

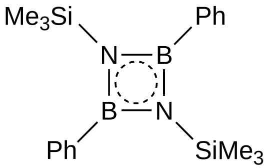

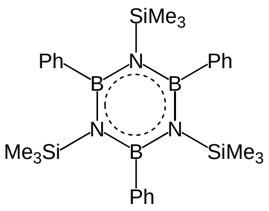

* grease

+ unknown side product

Figure S29. ^{29}Si INEPT NMR (Tol-d_8) of compound **8**.

NMR spectra of the reaction mixture of **8’**

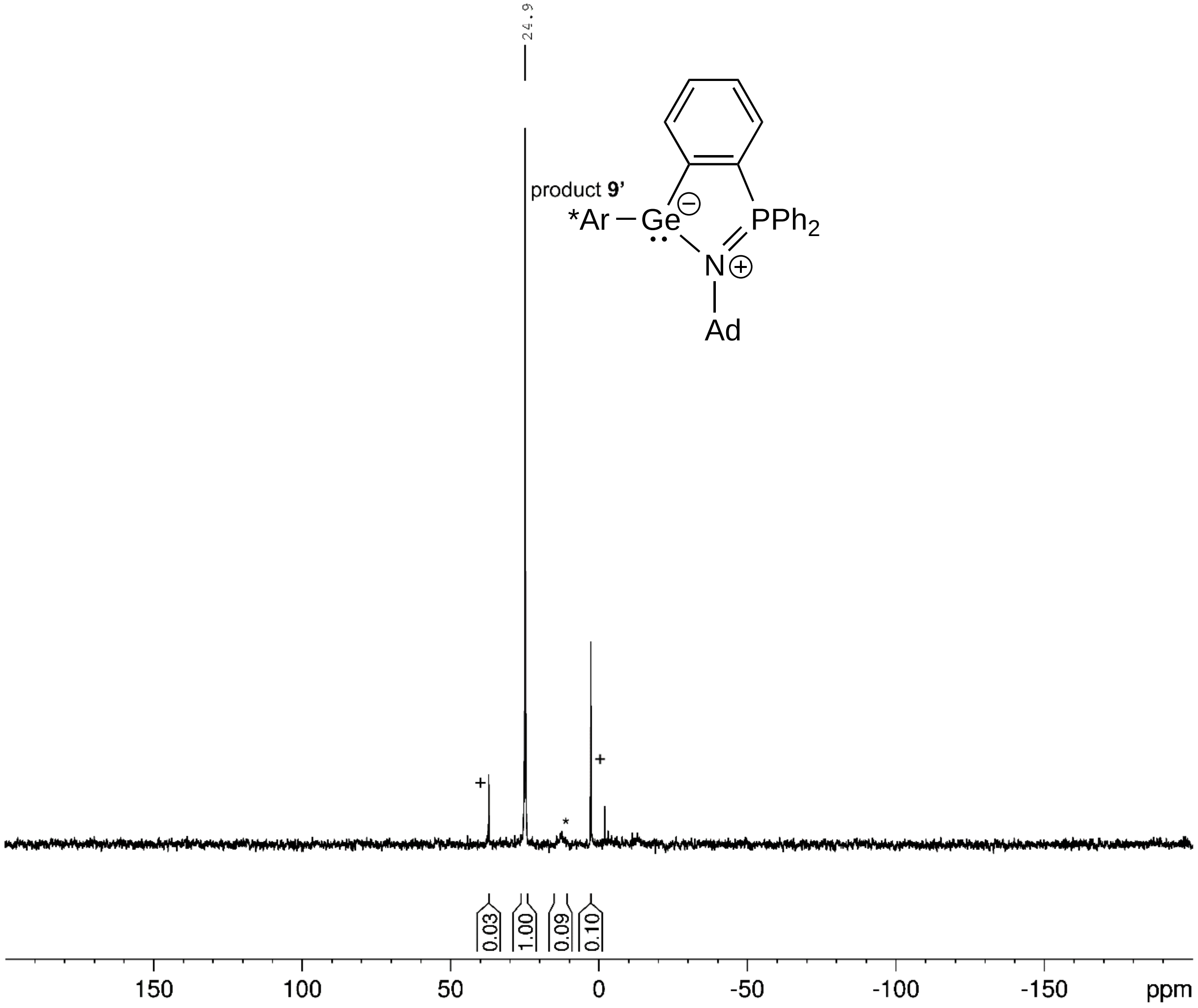


```
Current Data Parameters
NAME          DR421-300
EXPNO                31
PROCNO                1

F2 - Acquisition Parameters
Date_          20200819
Time              14.55 h
INSTRUM           spect
PROBHD   Z104275_0338 (
PULPROG          zgig30
TD                78426
SOLVENT            C6D6
NS                  256
DS                    0
SWH           73529.414 Hz
FIDRES         1.875128 Hz
AQ            0.5332968 sec
RG               204.67
DW                6.800 usec
DE                 6.50 usec
TE                298.0 K
D1           0.10000000 sec
D11          0.03000000 sec
TD0                   1
SFO1        121.4948510 MHz
NUC1                31P
P0                 4.00 usec
P1                12.00 usec
PLW1        11.36400032 W
SFO2        300.1314106 MHz
NUC2                 1H
CPDPRG[2        waltz16
PCPD2             90.00 usec
PLW2         8.26509953 W
PLW12        0.20000000 W

F2 - Processing parameters
SI                65536
SF          121.4948510 MHz
WDW                  EM
SSB      0
LB                10.00 Hz
GB       0
PC                 1.40
```

* educt 3

+ unknown side products in the reaction mixture

Figure S30. ^{31}P NMR (C_6D_6) of the reaction mixture of **8’**.

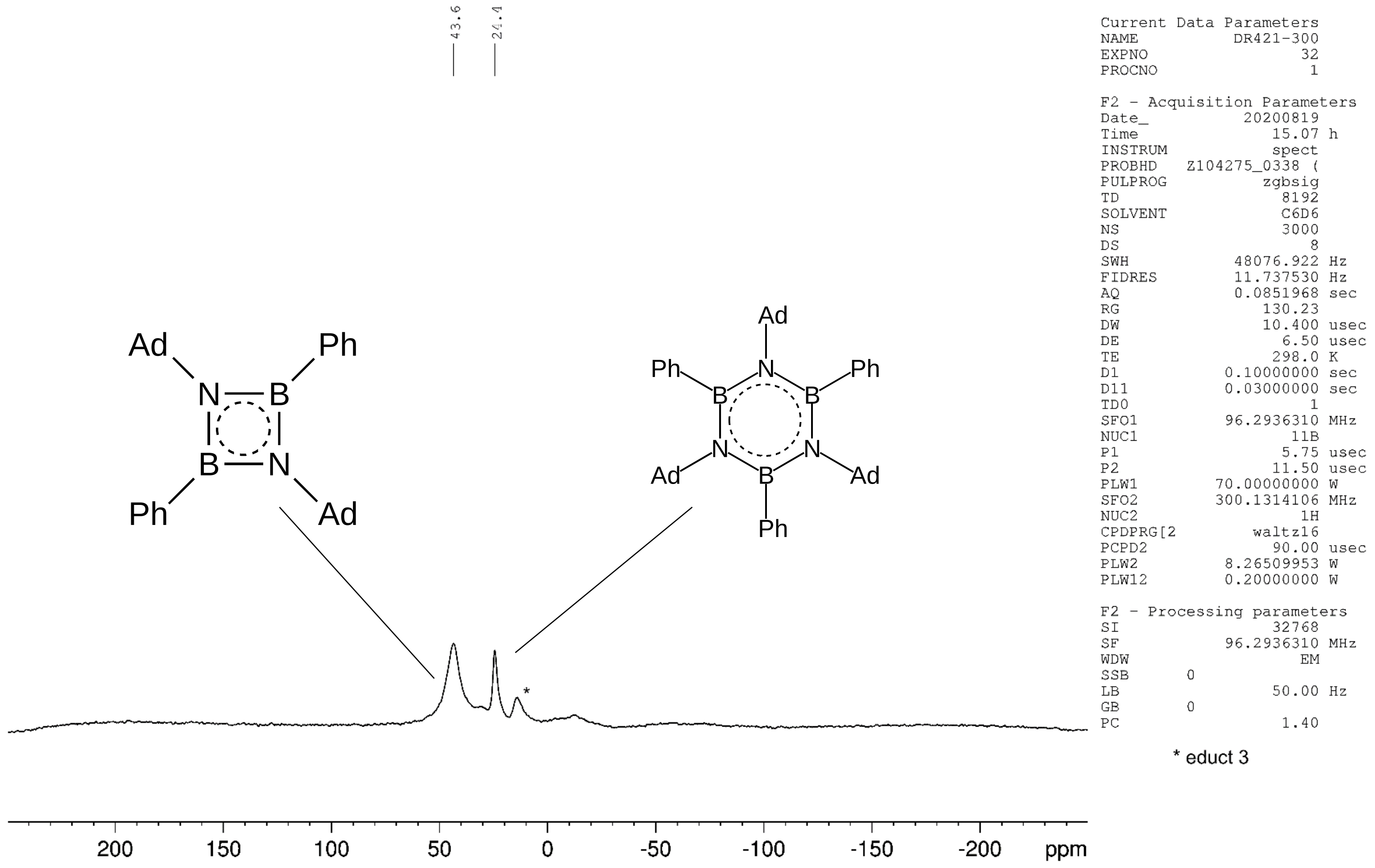

Figure S31. ^{11}B NMR (C_6D_6) of the reaction mixture of **8’**.

NMR spectra of compound **9**

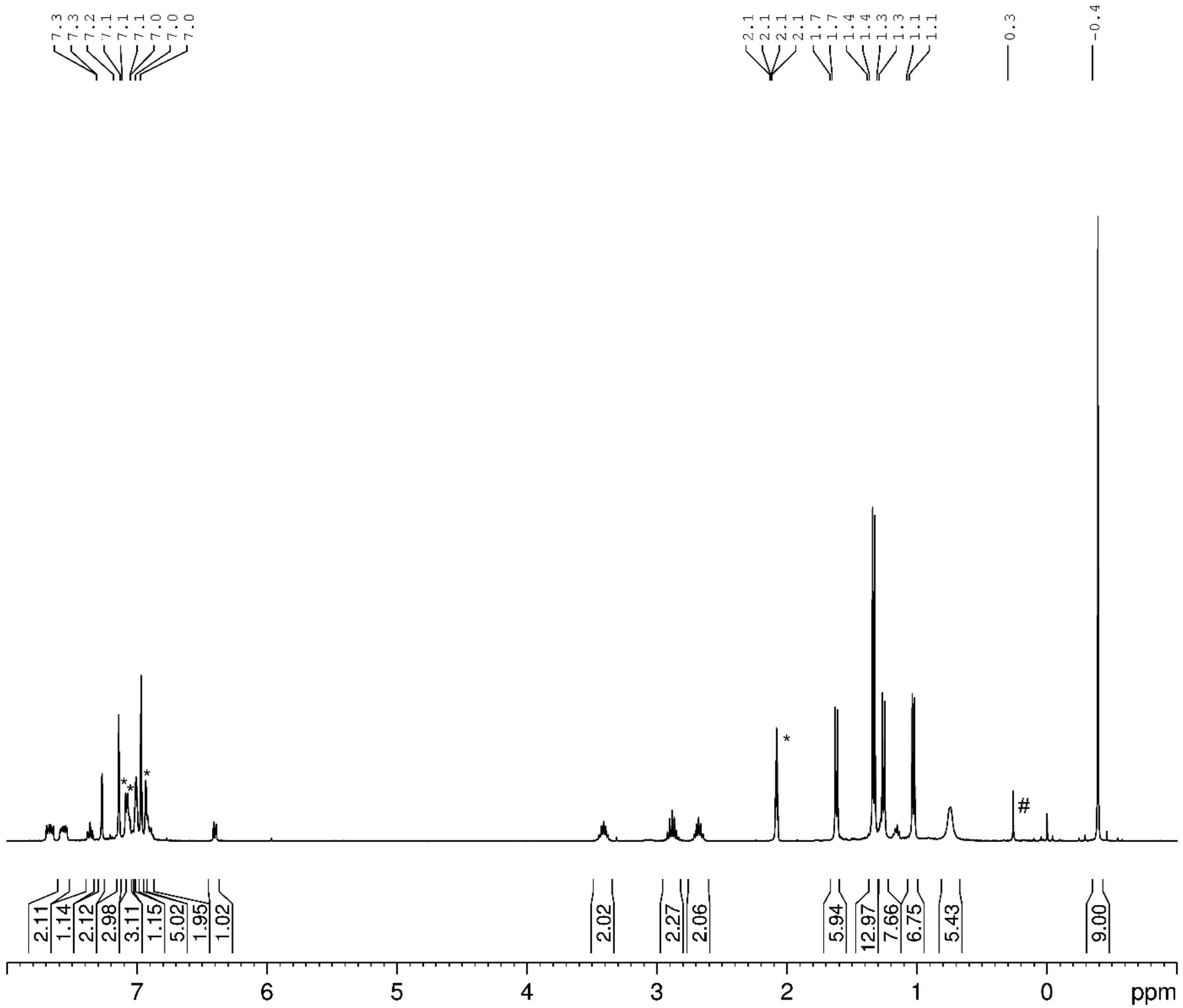

Current Data Parameters
NAME DR-GeNTMS-400NM
EXPNO 10
PROCNO 1

F2 - Acquisition Parameters
Date_ 20200810
Time 17.38
INSTRUM spect
PROBHD 5 mm QNP 1H/13
PULPROG zg30
TD 52656
SOLVENT Tol
NS 16
DS 0
SWH 8305.647 Hz
FIDRES 0.157734 Hz
AQ 3.1698911 sec
RG 256
DW 60.200 usec
DE 6.00 usec
TE 300.2 K
D1 1.00000000 sec
TD0 1

======== CHANNEL f1 ========
NUC1 1H
P1 14.60 usec
PL1 -3.00 dB
PL1W 16.03799057 W
SFO1 400.1120007 MHz

F2 - Processing parameters
SI 65536
SF 400.1100169 MHz
WDW EM
SSB 0
LB 0 Hz
GB 0
PC 1.00

# grease
* toluene-d8

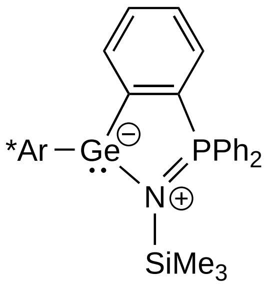

Figure S32. ^{1}H NMR (Tol-d_8) of compound **9**.

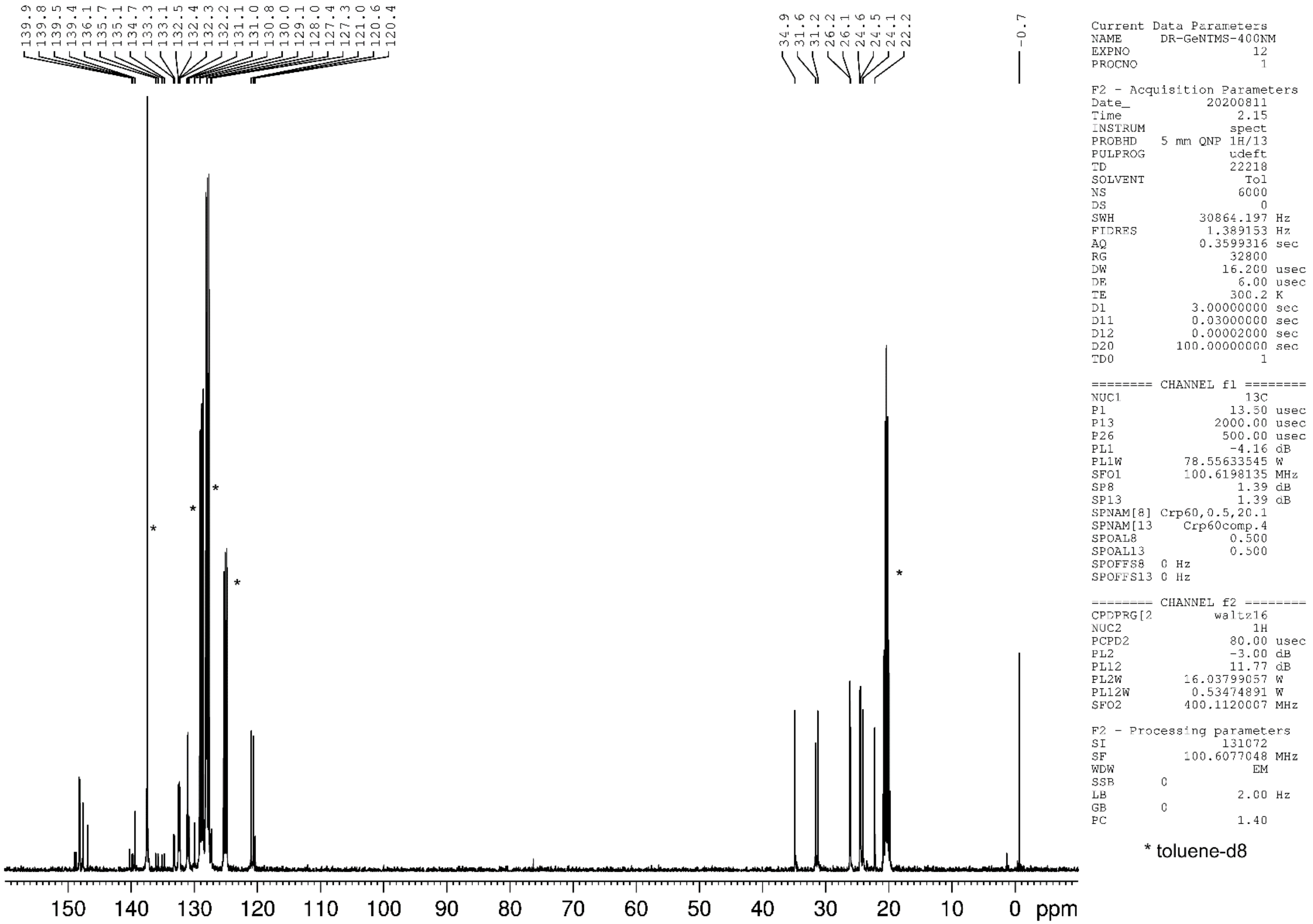

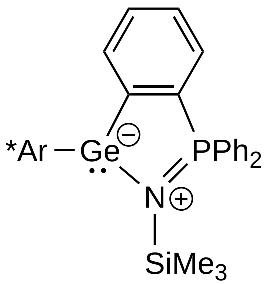

Figure S33. ^{13}C NMR (Tol-d_8) of compound **9**.

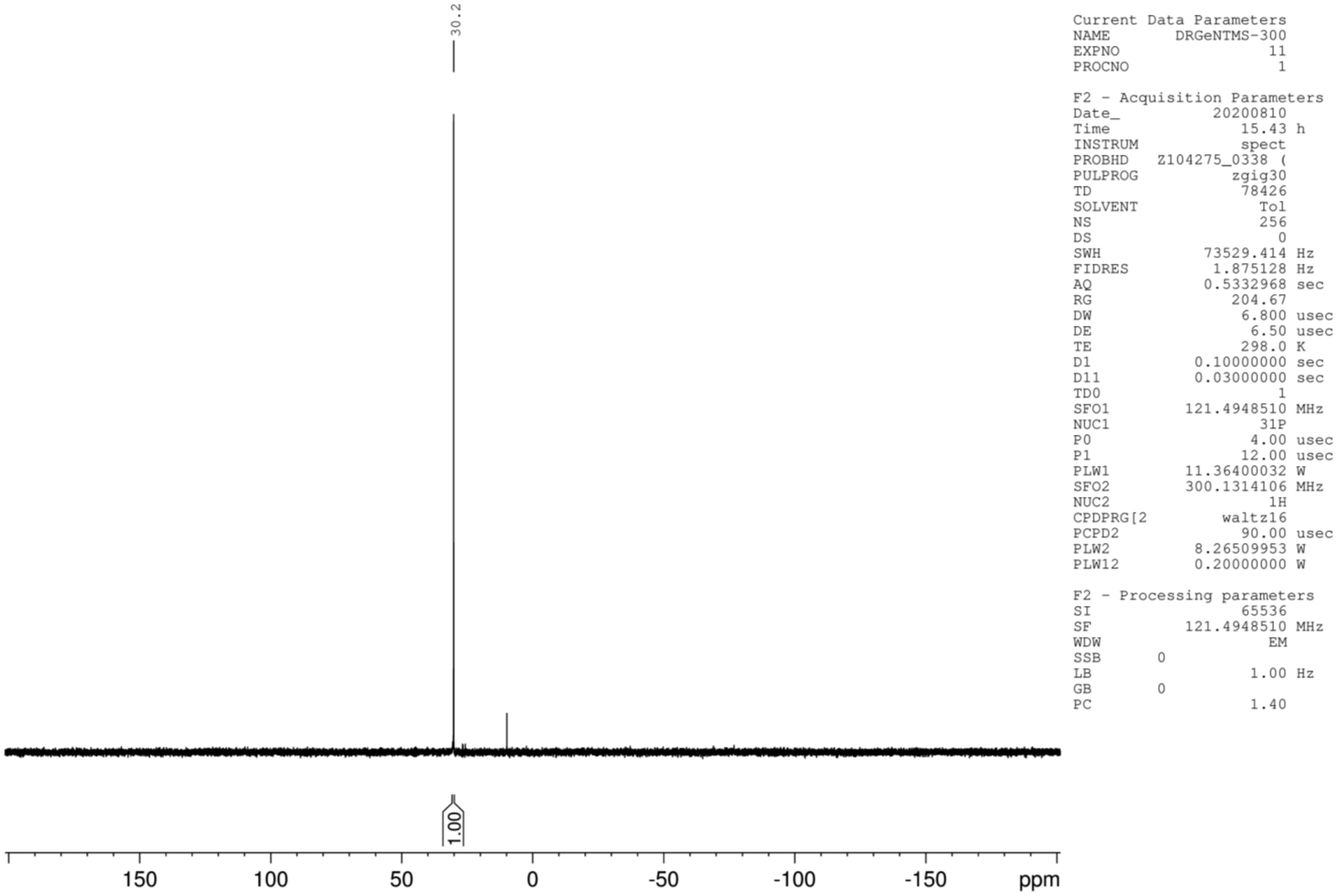

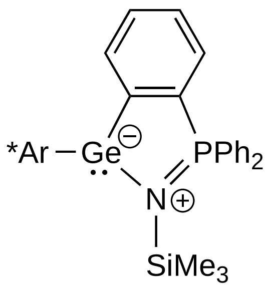

Figure S34. ^{31}P NMR (Tol-d_8) of compound **9**.

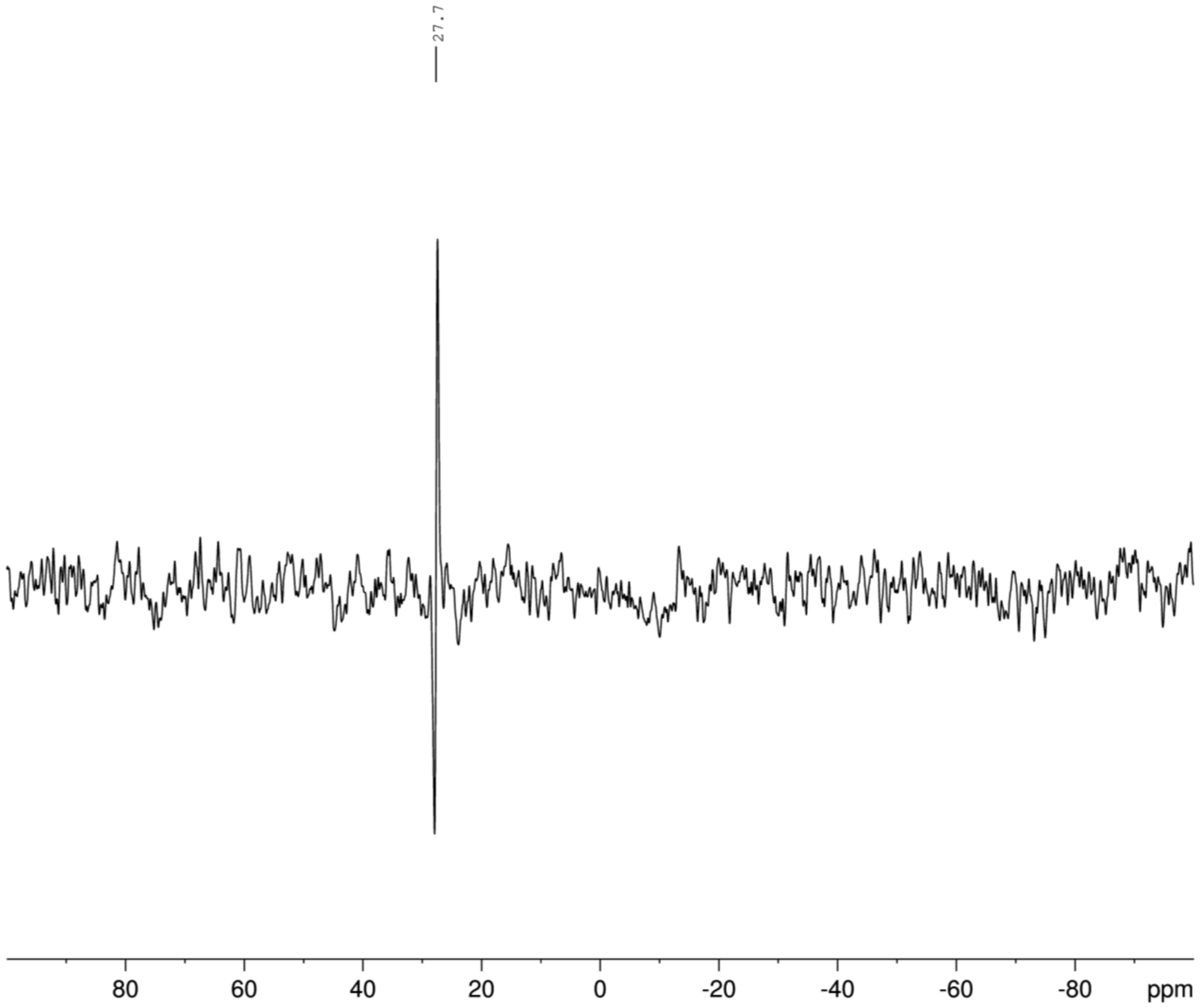


```
Current Data Parameters
NAME        DRGeNTMS-300
EXPNO                 14
PROCNO                 1

F2 - Acquisition Parameters
Date_           20200811
Time                8.46 h
INSTRUM            spect
PROBHD   Z104275_0338 (
PULPROG          ineptnd
TD                 39048
SOLVENT             C6D6
NS                   512
DS                     0
SWH            26315.789 Hz
FIDRES          1.347869 Hz
AQ             0.7419120 sec
RG                204.67
DW                19.000 usec
DE                  6.50 usec
TE                 298.0 K
CNST2          6.5999999
D1            2.00000000 sec
D4            0.03787879 sec
TD0                    1
SFO1          59.6229159 MHz
NUC1                29Si
P1                 10.30 usec
P2                 20.60 usec
PLW1         50.00000000 W
SFO2         300.1312005 MHz
NUC2                  1H
P3                 14.00 usec
P4                 28.00 usec
PLW2          8.26509953 W

F2 - Processing parameters
SI                 32768
SF            59.6273718 MHz
WDW                   EM
SSB      0
LB                 20.00 Hz
GB       0
PC                  1.00
```

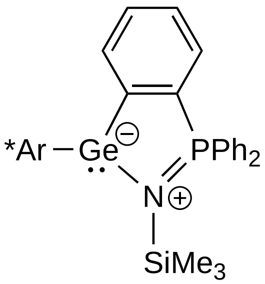

Figure S35. ^{29}Si INEPT NMR (Tol-d_8) of compound **9**.

NMR spectra of the reaction mixture of **9’**

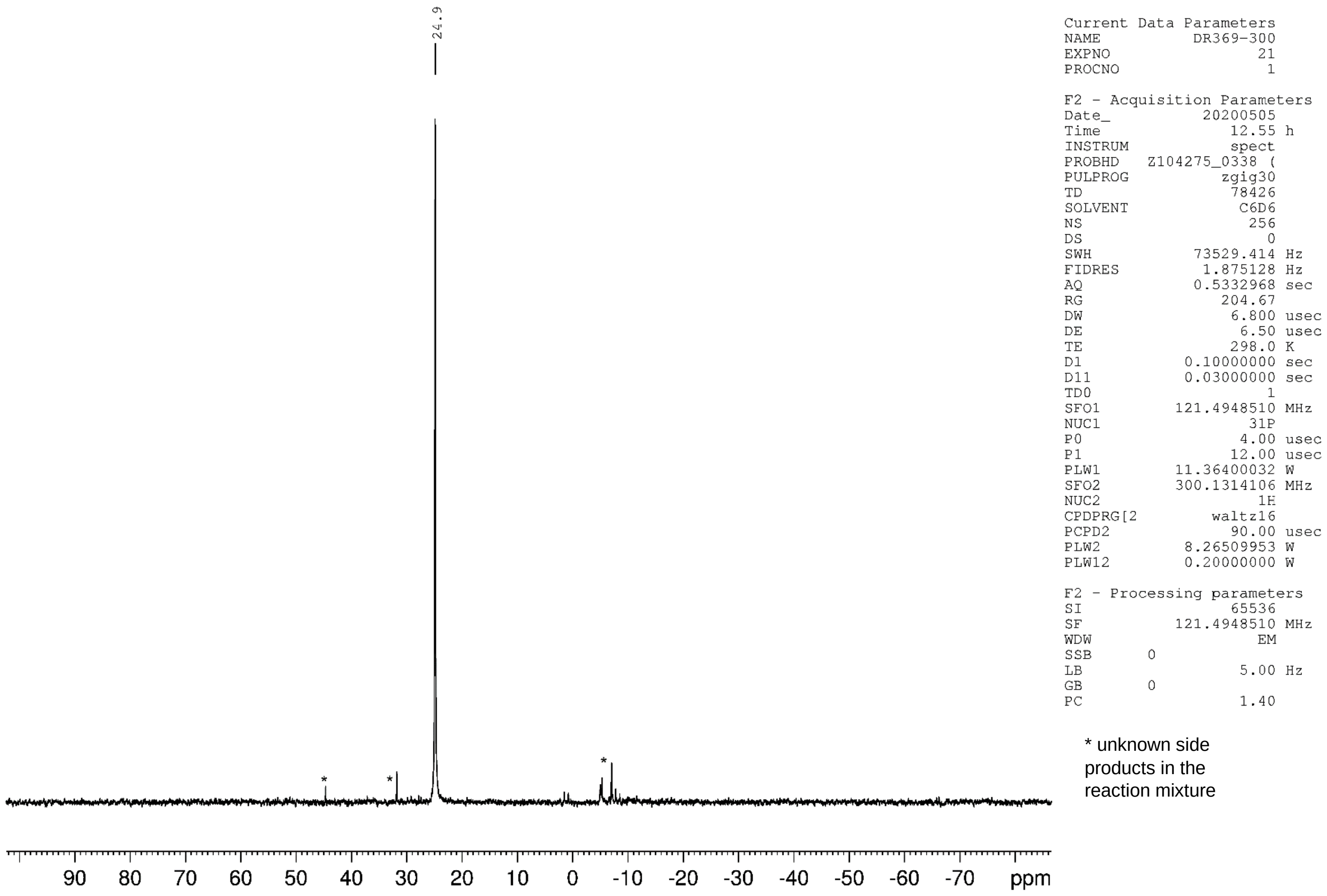

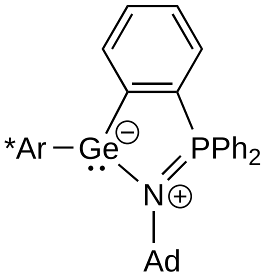

* unknown side products in the reaction mixture

Figure S36. ^{31}P NMR (C_6D_6) of the reaction mixture of **9’**.

^{1}H NMR spectra and UV-VIS spectra of reversible [2+2] cycloaddition

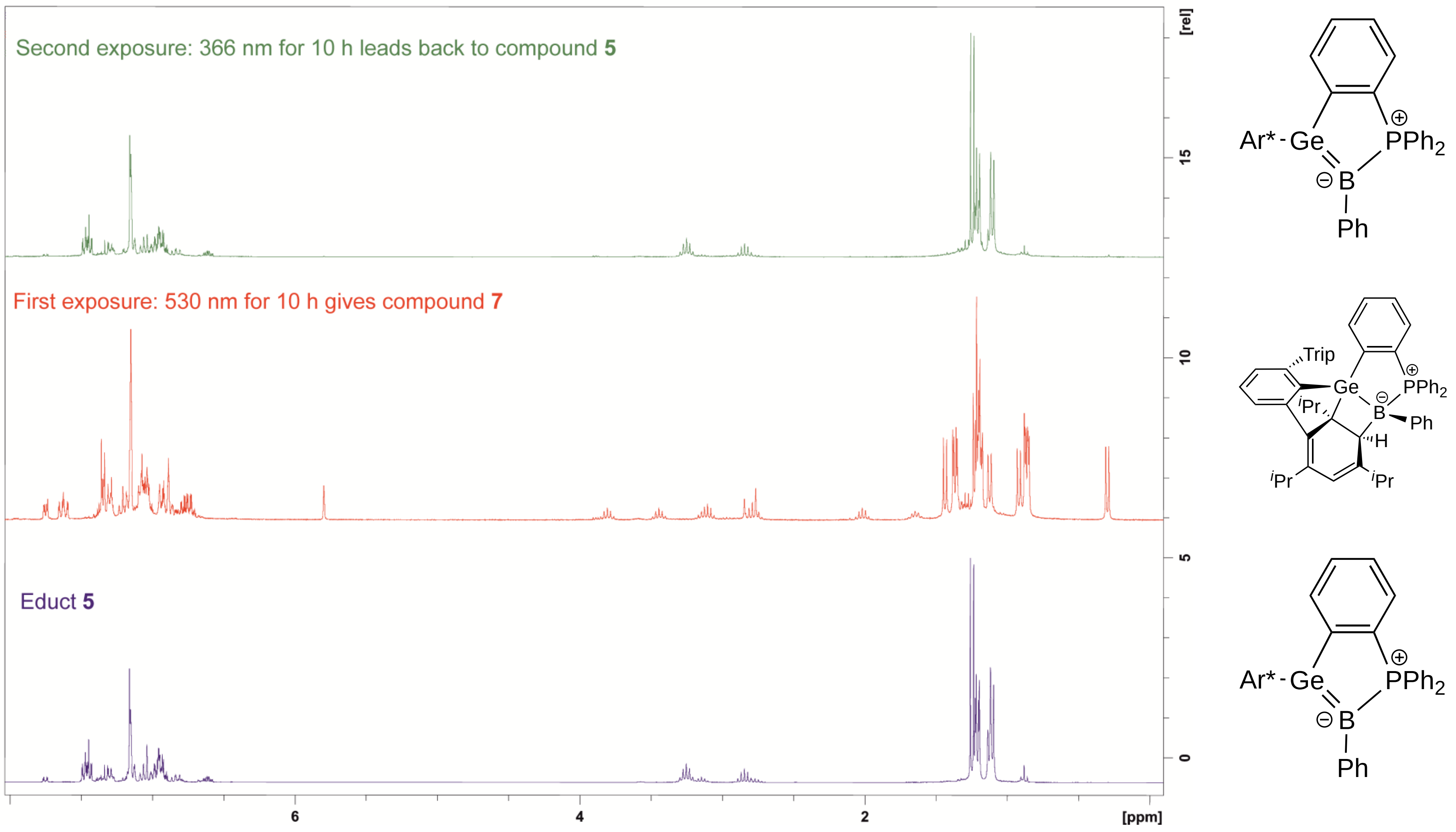

Figure S37. ^{1}H NMR spectra of reversible cycloaddition from **5** to **7** and reconversion to **5**.

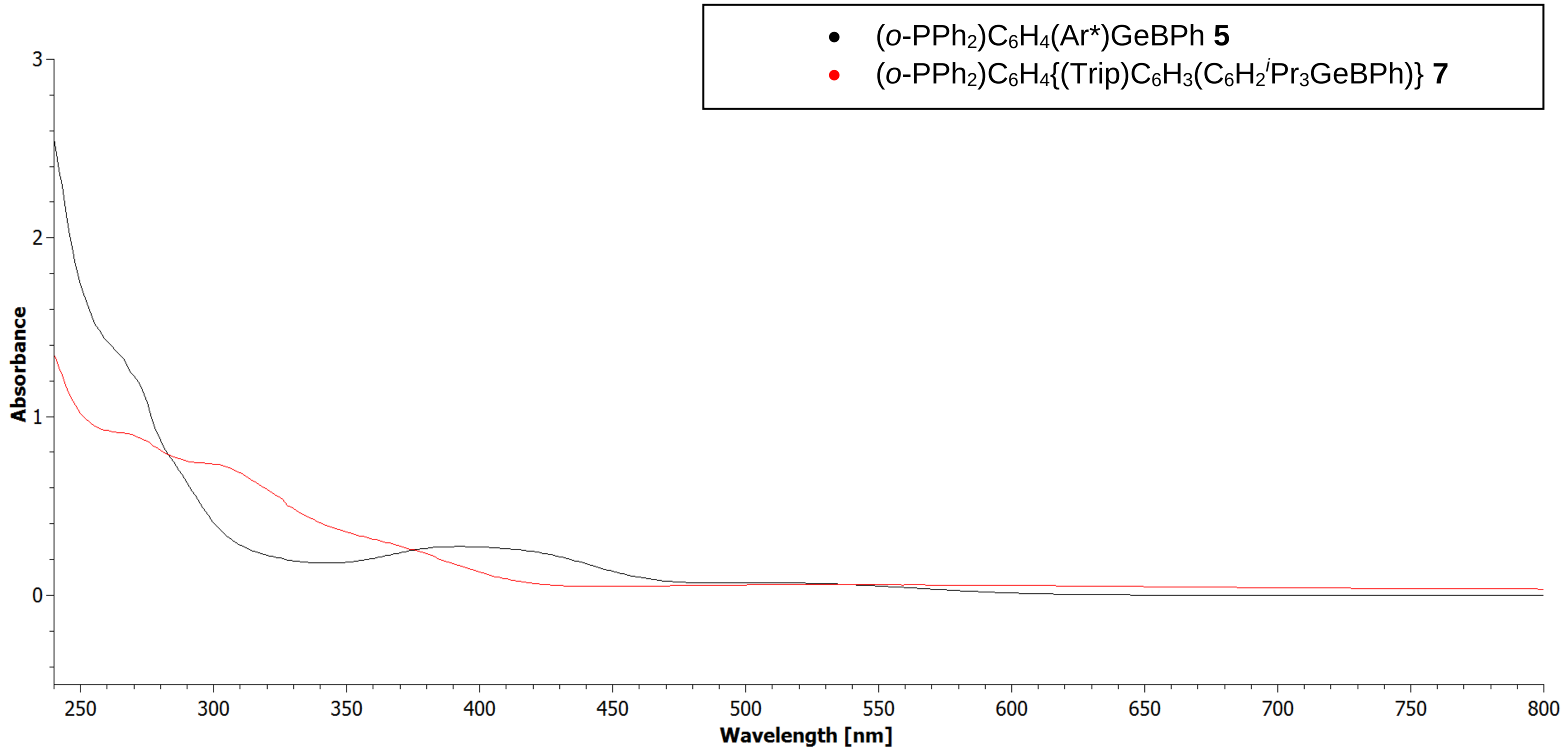

Figure S37. UV-Vis spectra of **5** (black, 1.33 x 10^{-4} mol/L) and **7** (red, 5.54 x 10^{-5} mol/L).

Table S1. Results of UV-Vis measurements.

Compound	Concentration [mol·L^{-1}]	Absorption max. [nm]	Absorbance	Mol. Absorptivity [L·mol^{-1}·cm^{-1}]
**5**	$1.33 \cdot 10^{-4}$	510	0.0685	$5.15 \cdot 10^{2}$
	$1.33 \cdot 10^{-4}$	390	0.2722	$2.05 \cdot 10^{3}$
**7**	$5.54 \cdot 10^{-5}$	375	0.2537	$4.58 \cdot 10^{3}$
	$5.54 \cdot 10^{-5}$	298	0.7375	$1.33 \cdot 10^{4}$
	$5.54 \cdot 10^{-5}$	266	0.9097	$1.64 \cdot 10^{4}$

Crystallography

X-ray data were collected with a Bruker Smart APEX II diffractometer with graphite-monochromated Mo Kα radiation or a Bruker APEX II Duo diffractometer with a Mo IµS microfocus tube and TRIUMPH monochromator. The programs used were Bruker's APEX2 v2011.8-0, including SADABS for absorption correction, SAINT for data reduction and SHELXS for structure solution, as well as the WinGX suite of programs version 1.70.01 or the GUI ShelXle, including SHELXL for structure refinement.[3]

Table S2. Selected crystallographic data for compound **3**, **4** and **8**.

	**3**	**4**	**8**
Empirical formula	$C_{57}H_{72}BClGeNPSi \cdot 2\ C_7H_8$	$C_{64}H_{78}BBrGeNP \cdot 3\ C_4H_8O$	$C_9H_{14}BNSi$
M [g/mol]	1133.33	1271.88	175.11
T [K]	100(2)	100(2)	100(2)
λ [Å]	0.71073	0.71073	0.71073
Crystal system	Triclinic	Triclinic	Monoclinic
Space group	P-1	P-1	$P2_1/n$
Z	4	2	4
a [Å]	11.0282(3)	11.1735(3)	11.1368(2)
b [Å]	19.8165(6)	15.3255(3)	5.99770(10)
c [Å]	31.6666(9)	21.2175(4)	15.2596(3)
α [°]	103.474(2)	107.7790(10)	90.0
β [°]	90.667(2)	95.4490(10)	99.2150(10)
γ [°]	106.154(2)	98.4500(10)	90.0
V [Å^3]	6442.5(3)	3384.27(13)	1006.11(3)
D_c [g/cm^3]	1.168	1.248	1.156
μ [mm^{-1}]	0.601	1.114	0.178
F(000)	2416	1352	376
Crystal size [mm]	0.21 x 0.19 x 0.17	0.22 x 0.21 x 0.17	0.24 x 0.22 x 0.19
θ range [°]	1.503 - 27.937	2.072 - 27.196	3.656 - 26.374
Limiting indices	-14<=h<=14<br>-26<=k<=25<br>-41<=l<=41	-14<=h<=14<br>-18<=k<=19<br>-27<=l<=27	-13<=h<=13<br>-7<=k<=7<br>-19<=l<=19
Reflections coll.	180418	54230	12326
Independent refl.	30582	14907	2044
R_{int}	0.0449	0.0587	0.0196
completeness	99.8	99.5	99.6
Absorption correction	multi-scan	multi-scan	multi-scan
Min. Max. transmis.	0.7003, 0.7456	0.6586, 0.7455	0.7214, 0.7454
Parameters/restraints	1442/0	769/0	112/0
R_1, wR_2 [I>2σI]	0.0523, 0.1184	0.0515, 0.1216	0.0274, 0.0801
R_1, wR_2 (all data)	0.0763, 0.1289	0.0801, 0.1331	0.0289, 0.0815
GooF	1.065	1.012	0.961
peak / hole [eÅ^{-3}]	1.042/-0.678	1.667/-0.675	0.362/-0.170
Flack			
CCDC	2033550	2033551	2033548

Table S3. Selected crystallographic data for compound **5**-**7** and **9**.

	**5**	**6**	**7**	**9**
Empirical formula	$C_{60}H_{68}BGeP$	$C_{60}H_{68}BCl_2GeP$	$C_{60}H_{68}BGeP \cdot 2\ C_7H_8$	$C_{57}H_{72}GeNPSi$
M [g/mol]	903.53	974.4	995.64	902.80
T [K]	100(2)	100(2)	100(2)	100(2)
λ [Å]	0.71073	0.71073	0.71073	0.71073
Crystal system	Monoclinic	Triclinic	Triclinic	Triclinic
Space group	$P2_1/n$	P-1	P-1	P-1
Z	4	4	4	4
a [Å]	14.3692(5)	13.2152(3)	12.1325(2)	11.9396(3)
b [Å]	23.1639(9)	16.3380(3)	13.9641(3)	13.0624(3)
c [Å]	15.5230(6)	17.0565(4)	17.6921(4)	17.6197(4)
α [°]	90.0	62.3770(10)	87.5990(10)	78.7240(10)
β [°]	100.6760(10)	72.3310(10)	75.2580(10)	72.440(2)
γ [°]	90.0	75.4220(10)	73.6390(10)	75.327(2)
V [$Å^3$]	5077.3(3)	3082.01(12)	2779.90(10)	2513.25(11)
D_c [g/cm^3]	1.182	1.050	1.189	1.193
μ [mm^{-1}]	0.672	0.641	0.620	0.702
F(000)	1920	1028	1060	964
Crystal size [mm]	0.22 x 0.17 x 0.16	0.22 x 0.21 x 0.18	0.21 x 0.16 x 0.13	0.22 x 0.21 x 0.17
θ range [°]	3.198 - 28.189	1.419 - 27.630	3.123 - 27.943	2.756 - 27.219
Limiting indices	-18<=h<=19	-17<=h<=17	-15<=h<=15	-15<=h<=15
	-30<=k<=30	-21<=k<=21	-18<=k<=18	-16<=k<=16
	-20<=l<=18	-22<=l<=22	-23<=l<=23	-22<=l<=22
Reflections coll.	87253	83530	47090	35236
Independent refl.	12414	14172	13125	11084
R_{int}	0.0505	0.0449	0.0459	0.0703
completeness	99.6	99.6	99.3	99.8
Absorption correction	multi-scan	multi-scan	multi-scan	multi-scan
Min. Max. transmis.	0.6697, 0.7457	0.6820, 0.7456	0.7149, 0.7456	0.6621, 0.7455
Parameters/restraints	591/0	598/0	709/46	565/40
R_1, wR_2 [I>2$σI$]	0.0496, 0.1153	0.0436, 0.1212	0.0425, 0.1000	0.0579, 0.1215
R_1, wR_2 (all data)	0.0722, 0.1264	0.0633, 0.1313	0.0585, 0.1075	0.1007, 0.1393
GooF	1.033	1.043	1.023	1.020
peak / hole [eÅ-^{3}]	0.713/-1.051	0.933/-0.306	1.949/-0.352	1.222/-1.463
Flack				
CCDC	2033553	2033549	2033552	2033554

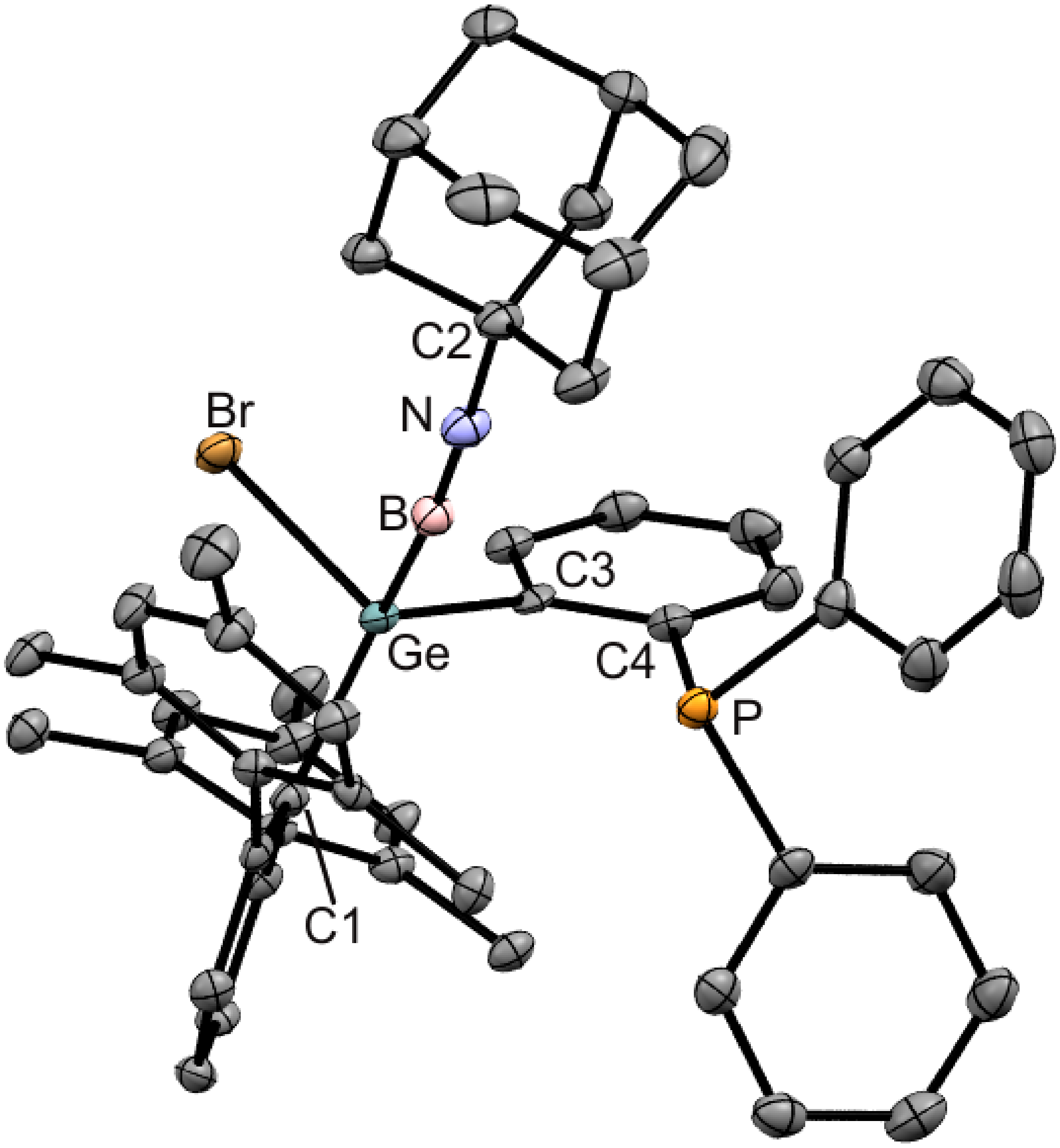

Figure S38. ORTEP of the molecular structure of **4**. Hydrogen atoms and CH_3-groups are omitted for clarity. Selected interatomic distances [Å] and angles [°] of **4**: B-Ge 2.006(4), B-N 1.231(5), Br-Ge 2.3584(4), Ge-C1 1.963(3), Ge-C3 1.969(3), C2-N 1.428(4), P-C4 1.839(3), C3-C4 1.401(4), N-B-Ge 165.1(3), B-N-C2 177.0(3), C1-Ge-C3 117.69(13), C1-Ge-B 118.50(13), C3-Ge-B 107.98(14), C1-Ge-Br 109.82(9), C3-Ge-Br 102.23(8), B-Ge-Br 97.49(10), C4-C3-Ge 120.1(2), C3-C4-P 118.0(2).

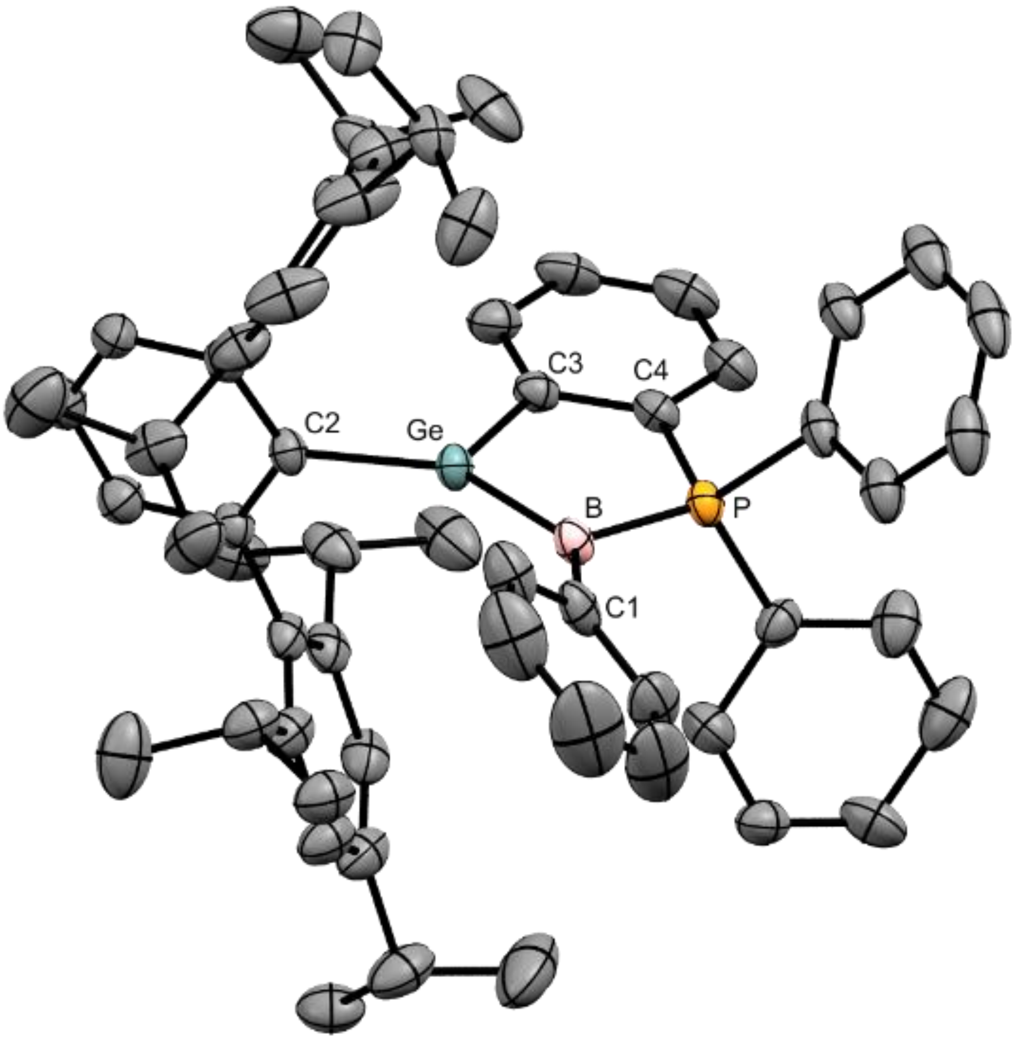

Figure S39. ORTEP of the molecular structure of **5**. Ellipsoids set at 50% probability. Hydrogen atoms omitted for clarity. Interatomic distances [Å] and angles [°] of **5**: Ge-B 1.899(3), Ge-C2 1.949(2), Ge-C3 1.954(2), B-P 1.898(3), B-C1 1.555(3), C3-C4 1.404(3), C4-P 1.814(2); C2-Ge-C3 109.69(10), C2-Ge-B 146.00(11), C3-Ge-B 103.62(11), Ge-B-P 101.05(12), B-P-C4 107.53(11), P-C4-C3 115.00(17), C4-C3-Ge 112.75(16), C1-B-Ge 134.60(19), C1-B-P 124.16(18).

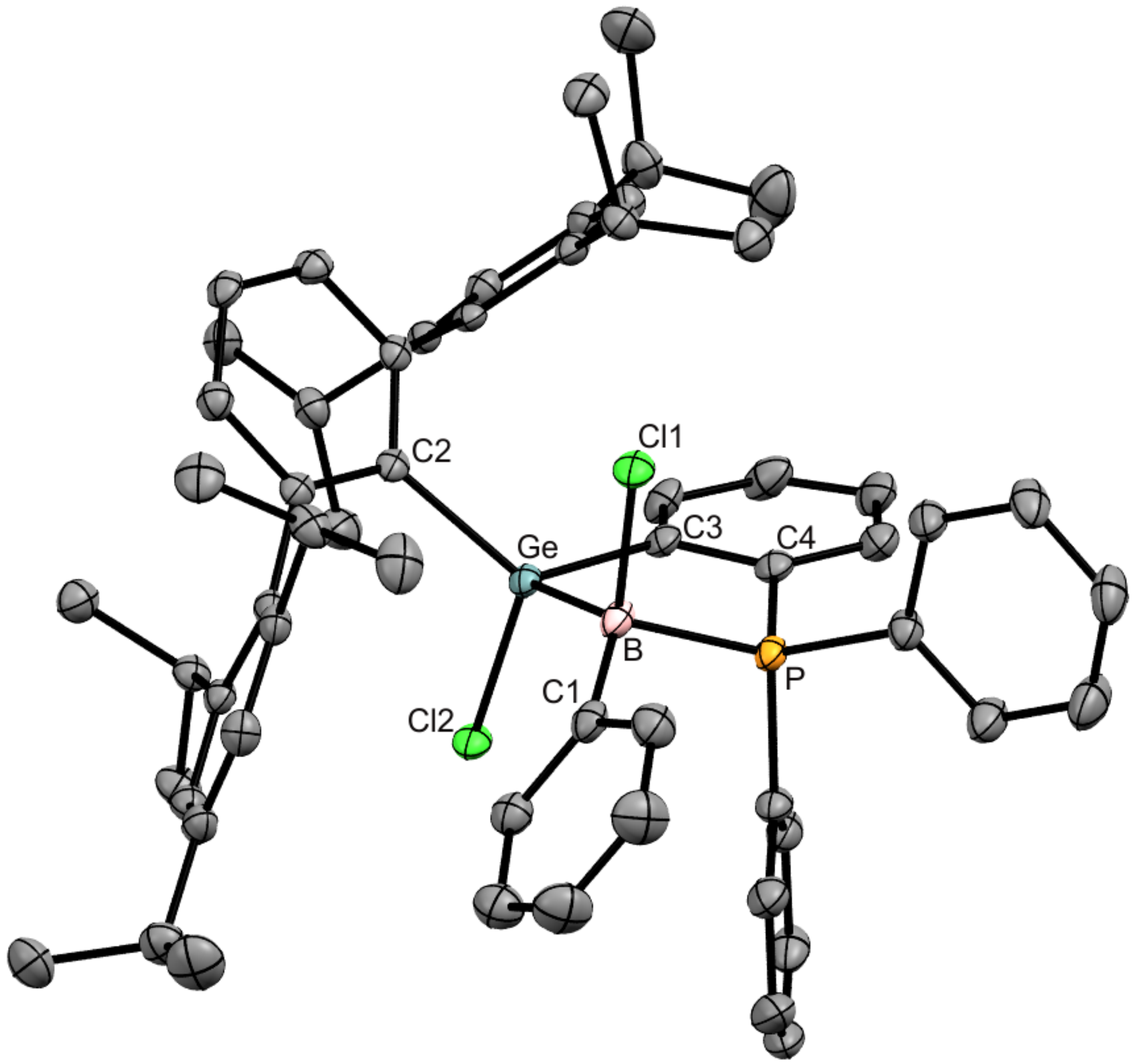

Figure S40. ORTEP of the molecular structure of **6**. Ellipsoids set at 50% probability. Hydrogen atoms are omitted for clarity. Selected interatomic distances [Å] and angles [°] of **6**: Ge-B 2.103(3), Ge-Cl2 2.2101(6), P-B 1.978(3), Cl1-B 1.890(3), Ge-C3 1.997(2), Ge-C2 2.000(2), C1-B 1.586(4), P-C4 1.796(3), P-B-Ge 96.63(12), C3-Ge-C2 118.51(9), C3-Ge-B 98.47(11), C2-Ge-B 117.31(9), C3-Ge-Cl2 96.68(7), C2-Ge1-Cl2 111.51(6), B-Ge-Cl2 112.07(7), C1-B-Cl1 113.93(17), C1-B-P 108.25(16), Cl1-B-P 105.75(13), C1-B-Ge 127.08(18), Cl1-B-Ge 102.38(12).

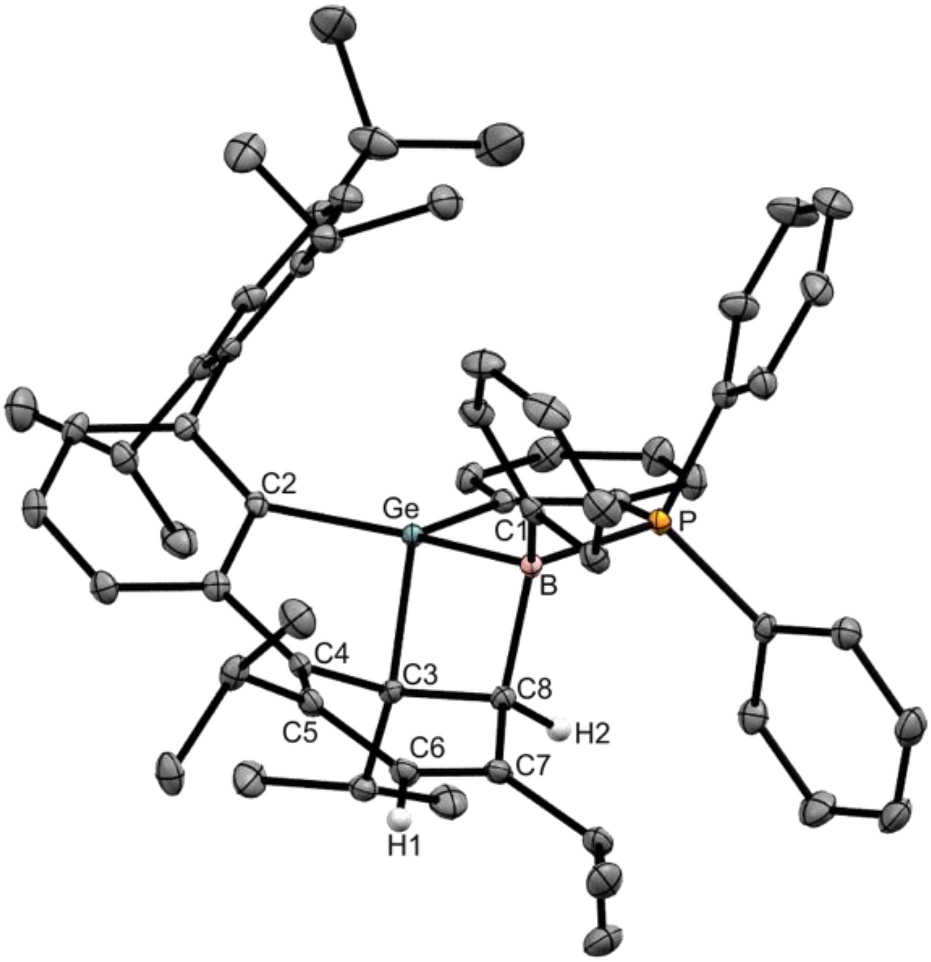

Figure S41. ORTEP of the molecular structure of **7**. Ellipsoids set at 50% probability. Hydrogen atoms except for H1 and H2 are omitted for clarity. Interatomic distances [Å] and angles [°] of **7**:Ge-B 2.124(2), B-P 1.968(2), Ge-C2 1.9466(19), Ge-C3 1.9939(17), B-C1 1.589(3), B-C8 1.682(3), C3-C4 1.546(3), C4-C5 1.360(3), C5-C6 1.474(3), C6-C7 1.342(3), C7-C8 1.503(2), C3-C8 1.548(3); C2-Ge-B 129.21(8), C2-Ge-C3 91.86(8), C3-Ge-B 74.62(8), C1-B-P 112.11(13), C1-B-Ge 125.79(14), Ge-B-P 96.32(9), C8-B-Ge 85.53(11), C8-B-P 110.07(13), C1-B-C8 122.26(15), C3-C8-B 101.23(14), C7-C8-B 117.92(15), C3-C8-C7 112.34(15), C8-C7-C6 117.69(17), C7-C6-C5 121.92(17), C6-C5-C4 119.25(18), C5-C4-C3 119.33(17), C4-C3-C8 109.08(15).

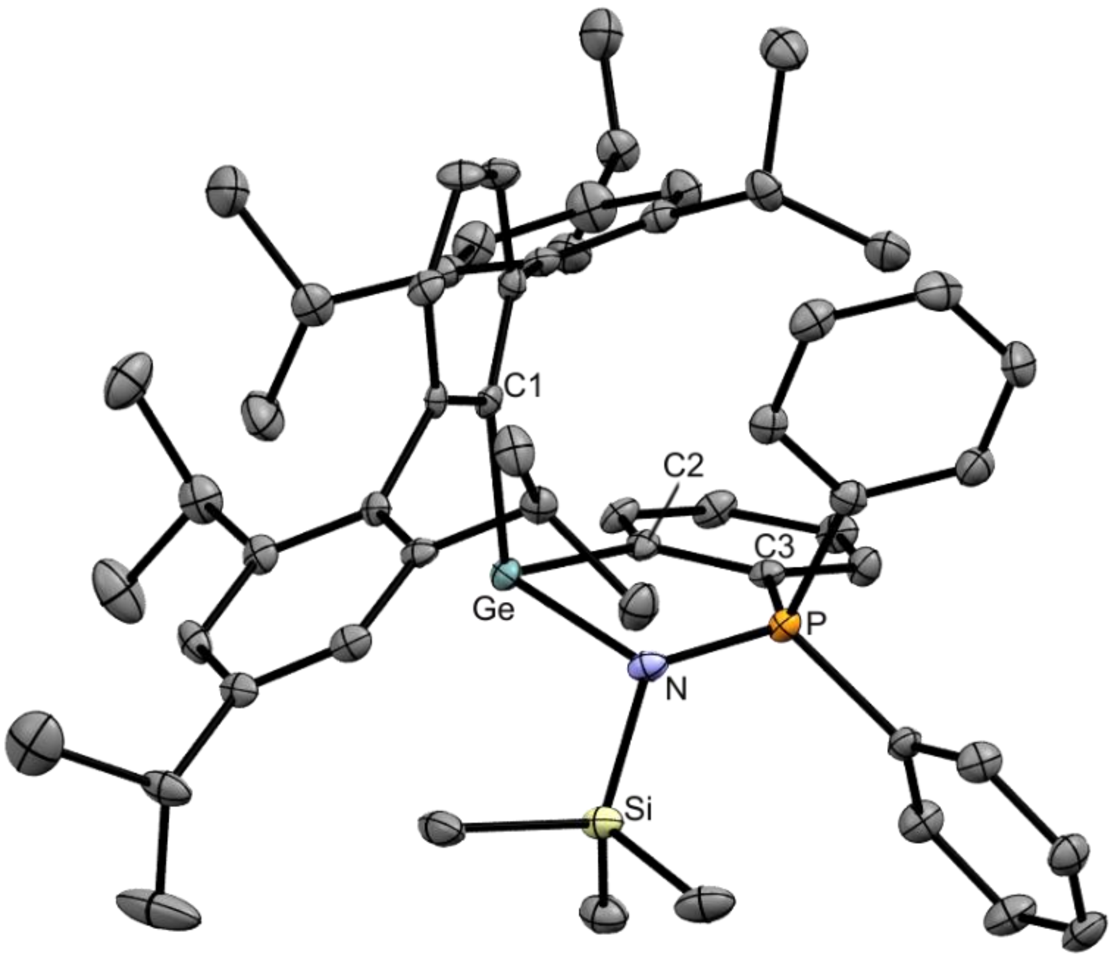

Figure S42. ORTEP of the molecular structure of **9**. Ellipsoids set at 50% probability. Hydrogen atoms are omitted for clarity. Selected interatomic distances [Å] and angles [°] of **9**: Ge-N 2.153(3), N-P 1.611(3), N-Si 1.768(3), Ge-C1 2.093(3), Ge-C2 2.029(3), P-C3 1.786(3), P-N-Ge 110.60(14), Si-N-Ge 108.40(14), C1-Ge-N 102.96(11), C2-Ge-N 85.27(11), C2-Ge-C1 103.35(12), C3-C2-Ge 119.8(2), P-N-Si 123.93(16), N-P-C3 106.54(15).

Computational Methods

The structures of all stationary points were optimized at the B3LYP-D3BJ/6-31G* level of theory. The nature of the stationary points as minima or transition states was confirmed by analytic computations of second derivatives, and the transition states were found to have only one imaginary vibrational frequency as required. Intrinsic reaction coordinate (IRC)[4] computations were performed to confirm the connectivity of transition states and minima, and to confirm concertedness of reaction steps. The energies were refined using the B3LYP-D3BJ/6-31G* geometries by performing DLPNO-CCSD(T)/cc-pVTZ single point computations using default PNO settings. All geometry optimizations were performed with Gaussian 16,[5] while the DLPNO-CCSD(T) runs were done with Orca.[6]

Computation of the IRC confirms that the ring opening of the triazole intermediate through **TS2** results in fragmentation to a van der Waals complex of the three product molecules without any further intermediate. The electronic energy of this complex with respect to separated reactants (ΔE_{el} = -65.0 kcal mol^{-1}, DLPNO-CCSD(T)/cc-pVTZ), is lower than that of the separated products (ΔE_{el} = -54.8 kcal mol^{-1}), but its fragmentation to three product molecules is favored entropically ($\Delta G°$ = -52.6 kcal mol^{-1} for the complex, but $\Delta G°$ = -71.5 kcal mol^{-1} for separated products). For this reason, this complex is not displayed in Fig. 3, but its Cartesian coordinates (“van der Waals complex of reaction products”) are given below.

Likewise, **TS1** connects the triazole to a van der Waals complex of the reactants without additional intermediates according to IRC computations. This complex is bound on the electronic energy scale (ΔE_{el} = -11.2 kcal mol^{-1}, DLPNO-CCSD(T)/cc-pVTZ) with respect to separated reactants, but not on the Gibbs free energy scale ($\Delta G°$ = 4.0 kcal mol^{-1}). For this reason, this complex is not displayed in Fig. 3, but its Cartesian coordinates (“van der Waals complex of reactants”) are given below.

Cartesian Coordinates

All Cartesian coordinates are given in Å, electronic energies in Hartrees, and were obtained at the B3LYP-D3BJ/6-31G* level of theory.

```
131
educt, -4769.00715109
Ge      -0.171954000     -0.467731000      0.092170000
P       -0.321785000      2.423675000      0.300790000
C        1.140169000     -4.476027000      0.470322000
H        2.070101000     -4.984329000      0.709647000
C        1.140982000     -3.076735000      0.366789000
C       -1.272494000      0.198081000      1.542917000
C       -0.021150000     -5.206950000      0.243954000
H       -0.003427000     -6.291446000      0.307687000
C        1.692817000      1.445133000     -1.666376000
C       -2.077331000      2.296797000      2.522445000
H       -2.134510000      3.381967000      2.497085000
C       -0.066625000     -2.408911000      0.092168000
C       -1.336357000      1.606656000      1.568777000
C        2.446058000     -2.352823000      0.500773000
C        3.420463000     -2.491616000     -0.515511000
C       -1.921162000     -0.494491000      2.563786000
H       -1.864257000     -1.577695000      2.594641000
C       -1.249545000     -3.152919000     -0.145367000
C       -1.486290000      3.421569000     -0.695349000
C       -3.579819000     -2.306403000      0.424881000
C       -1.205580000     -4.548284000     -0.085210000
H       -2.096774000     -5.124920000     -0.303339000
B        0.606089000      1.079894000     -0.624811000
C       -1.060246000     -0.608296000     -3.553426000
H       -1.879559000     -0.116798000     -4.091533000
H       -0.308750000     -0.895342000     -4.296791000
H       -0.599785000      0.108432000     -2.869983000
C       -2.487545000     -2.376332000     -0.472612000
C       -3.787083000     -3.356356000      1.510337000
H       -2.803093000     -3.720259000      1.826040000
C        2.735194000     -1.584313000      1.648694000
```

C	3.079783000	-3.202462000	-1.822664000
H	2.444448000	-4.059664000	-1.582303000
C	0.792403000	3.609107000	1.115538000
C	-4.568807000	-4.547563000	0.915870000
H	-5.579144000	-4.227014000	0.635827000
H	-4.658950000	-5.358735000	1.648229000
H	-4.094702000	-4.946057000	0.015386000
C	-2.629051000	0.190870000	3.558082000
H	-3.106344000	-0.367066000	4.359208000
C	-1.530896000	-1.847365000	-2.785015000
H	-0.642758000	-2.329931000	-2.374681000
C	-1.741392000	4.778830000	-0.474217000
H	-1.196645000	5.316302000	0.295702000
C	2.740577000	5.268059000	2.250135000
H	3.496773000	5.911479000	2.690829000
C	-2.487023000	-1.558946000	-1.630694000
C	4.292369000	-3.743660000	-2.588209000
H	4.919158000	-2.936551000	-2.984288000
H	3.954723000	-4.338510000	-3.444190000
H	4.919469000	-4.381610000	-1.955355000
C	-2.689773000	5.440741000	-1.256170000
H	-2.885589000	6.496003000	-1.087888000
C	1.561908000	4.456808000	0.301197000
H	1.418147000	4.454629000	-0.774584000
C	-2.731004000	1.580523000	3.531965000
H	-3.297449000	2.106166000	4.294739000
C	3.990083000	-0.984261000	1.760840000
H	4.216134000	-0.399430000	2.648438000
C	2.528639000	5.282451000	0.869759000
H	3.121834000	5.932311000	0.232976000
C	2.973149000	0.873717000	-1.554521000
H	3.158418000	0.181562000	-0.744309000
C	1.742275000	-1.435149000	2.793668000
H	0.758264000	-1.745613000	2.434446000
C	-4.532592000	-1.301466000	0.249283000
H	-5.335048000	-1.206940000	0.972621000
C	-4.459621000	-0.386262000	-0.802117000
C	-3.471328000	-0.576622000	-1.765880000
H	-3.467145000	0.062478000	-2.643532000
C	4.663470000	-1.870959000	-0.354646000
H	5.400857000	-1.971572000	-1.140844000
C	2.248568000	-2.257394000	-2.711684000
H	1.453031000	-1.796604000	-2.124898000
H	1.800170000	-2.798706000	-3.553203000
H	2.867627000	-1.446253000	-3.106119000
C	1.983626000	4.421628000	3.061042000
H	2.148112000	4.403516000	4.134680000
C	1.013663000	3.591943000	2.499324000
H	0.434539000	2.929608000	3.132312000
C	1.483754000	2.350663000	-2.725691000
H	0.502874000	2.802861000	-2.854677000
C	-4.535351000	-2.862388000	2.757588000
H	-4.110511000	-1.939860000	3.154561000
H	-4.496779000	-3.627969000	3.540462000
H	-5.593661000	-2.675970000	2.542446000
C	2.104229000	-2.378079000	3.953174000
H	2.116757000	-3.421462000	3.619112000
H	1.375533000	-2.286757000	4.767693000
H	3.096145000	-2.141161000	4.355766000
C	-6.886294000	0.349562000	-0.927782000
H	-7.173587000	-0.167531000	-0.004996000
H	-7.070738000	-0.332821000	-1.764600000
H	-7.545925000	1.218033000	-1.039374000
C	4.969194000	-1.108816000	0.774159000
C	-2.195743000	-2.866892000	-3.729226000
H	-2.464674000	-3.782525000	-3.191340000
H	-1.515710000	-3.134530000	-4.546732000
H	-3.111230000	-2.452489000	-4.167975000
C	3.993095000	1.183489000	-2.451475000
H	4.967364000	0.713384000	-2.340327000
C	1.623542000	0.023048000	3.261804000
H	2.509245000	0.346679000	3.820162000
H	0.752945000	0.140705000	3.915929000
H	1.502255000	0.681246000	2.398191000
C	-5.414695000	0.789570000	-0.884303000
H	-5.197592000	1.322595000	-1.819201000
C	3.762919000	2.086299000	-3.492631000
H	4.556982000	2.331483000	-4.193171000

C	2.502159000	2.672144000	-3.623914000
H	2.309165000	3.372528000	-4.433131000
C	-5.156922000	1.766647000	0.276621000
H	-5.800185000	2.650487000	0.190042000
H	-4.115268000	2.097777000	0.286317000
H	-5.359609000	1.288823000	1.241946000
C	-3.382590000	4.751629000	-2.253119000
H	-4.116154000	5.272206000	-2.862193000
C	-3.129564000	3.394693000	-2.471002000
H	-3.665566000	2.856121000	-3.247371000
C	-2.184392000	2.730627000	-1.694557000
H	-1.975570000	1.675430000	-1.834939000
C	6.288799000	-0.369823000	0.949294000
H	6.635736000	-0.574027000	1.973026000
C	6.068094000	1.151899000	0.835553000
C	7.393424000	-0.824220000	-0.010209000
H	7.556173000	-1.906215000	0.041890000
H	7.151064000	-0.568437000	-1.048734000
H	8.336674000	-0.325092000	0.236616000
H	5.707752000	1.417932000	-0.163600000
H	7.003519000	1.693490000	1.020885000
H	5.320718000	1.502027000	1.554457000

16
trimethylsilyl azide, -573.520457572

N	-3.036000000	0.000543000	0.025935000
N	-1.967677000	-0.002793000	-0.380295000
N	-0.861166000	-0.007146000	-0.906131000
Si	0.662562000	0.000053000	0.020502000
C	0.710817000	-1.532916000	1.108944000
H	1.636500000	-1.567127000	1.696295000
H	-0.128797000	-1.546431000	1.813839000
H	0.659129000	-2.447116000	0.507595000
C	0.719644000	1.559486000	1.070231000
H	0.671189000	2.458570000	0.446251000
H	-0.118786000	1.594501000	1.775786000
H	1.646437000	1.604068000	1.655142000
C	1.998917000	-0.019833000	-1.290084000
H	2.995421000	-0.015913000	-0.833171000
H	1.918537000	-0.913404000	-1.918453000
H	1.922144000	0.857460000	-1.941426000

147
van der Waals complex of reactants, -5342.55051753

Ge	0.125335000	-0.637870000	-0.229255000
P	0.799976000	2.006766000	-1.202186000
C	-0.925505000	-4.720826000	0.166700000
H	-1.821677000	-5.330466000	0.110545000
C	-1.040981000	-3.329806000	0.058845000
C	0.119779000	-0.433158000	-2.158974000
C	0.315703000	-5.322187000	0.367978000
H	0.392051000	-6.403829000	0.437066000
C	1.172314000	1.769684000	1.706505000
C	0.464906000	1.246840000	-3.912187000
H	0.698454000	2.269597000	-4.196700000
C	0.117387000	-2.531840000	0.166185000
C	0.367438000	0.892230000	-2.570736000
C	-2.402440000	-2.715200000	-0.038531000
C	-2.939514000	-2.084558000	1.107551000
C	-0.012202000	-1.399569000	-3.155242000
H	-0.160216000	-2.437468000	-2.879280000
C	1.355438000	-3.133016000	0.451300000
C	2.485736000	2.555117000	-1.655016000
C	3.551639000	-2.160654000	-0.288954000
C	1.448421000	-4.529435000	0.527510000
H	2.413031000	-4.983308000	0.735734000
B	0.767357000	1.014585000	0.403796000
C	1.846000000	-0.740314000	4.179734000
H	2.694001000	-0.992155000	4.828638000
H	0.949053000	-0.734477000	4.808457000
H	1.992355000	0.268098000	3.793697000
C	2.576313000	-2.307789000	0.715201000
C	3.329074000	-2.726848000	-1.686730000
H	2.290816000	-3.066193000	-1.742792000
C	-3.187945000	-2.825868000	-1.206037000
C	-2.204627000	-2.086572000	2.443161000

H	-1.220552000	-2.530370000	2.289531000
C	-0.213958000	3.514472000	-1.267565000
C	4.232776000	-3.945088000	-1.936600000
H	5.291930000	-3.665558000	-1.885682000
H	4.043934000	-4.372181000	-2.928693000
H	4.058112000	-4.726336000	-1.189190000
C	0.067099000	-1.054341000	-4.509032000
H	-0.043340000	-1.827584000	-5.264737000
C	1.674760000	-1.773777000	3.059475000
H	0.731973000	-1.543240000	2.552481000
C	2.763454000	3.786492000	-2.256375000
H	1.974019000	4.518704000	-2.389760000
C	-1.739849000	5.858068000	-1.264199000
H	-2.328263000	6.771195000	-1.268582000
C	2.762579000	-1.714580000	1.987247000
C	-2.935440000	-2.974309000	3.463619000
H	-3.937016000	-2.587661000	3.685280000
H	-2.373725000	-3.015872000	4.404611000
H	-3.046390000	-3.996626000	3.085665000
C	4.061097000	4.071349000	-2.686351000
H	4.277777000	5.031759000	-3.145759000
C	0.079615000	4.546117000	-0.360258000
H	0.897765000	4.430121000	0.344485000
C	0.293262000	0.265010000	-4.895211000
H	0.360292000	0.528587000	-5.946468000
C	-4.477664000	-2.293479000	-1.202898000
H	-5.090838000	-2.389896000	-2.095040000
C	-0.679653000	5.713977000	-0.365005000
H	-0.445028000	6.511609000	0.334043000
C	0.235396000	2.161086000	2.679809000
H	-0.807162000	1.917913000	2.519486000
C	-2.715580000	-3.575618000	-2.442903000
H	-1.637362000	-3.730690000	-2.348206000
C	4.747728000	-1.499886000	0.017289000
H	5.514716000	-1.420238000	-0.749050000
C	4.986010000	-0.969667000	1.286372000
C	3.970086000	-1.064843000	2.240997000
H	4.135215000	-0.612283000	3.212633000
C	-4.229721000	-1.547227000	1.052158000
H	-4.631991000	-1.074729000	1.942499000
C	-1.983364000	-0.668814000	2.977593000
H	-1.488641000	-0.065914000	2.215660000
H	-1.359029000	-0.684129000	3.877933000
H	-2.930438000	-0.179653000	3.229161000
C	-2.044755000	4.825439000	-2.152531000
H	-2.872426000	4.931392000	-2.848156000
C	-1.287277000	3.653574000	-2.155846000
H	-1.529422000	2.848460000	-2.840750000
C	2.517501000	2.126999000	1.935145000
H	3.272039000	1.833260000	1.213195000
C	3.505725000	-1.667921000	-2.785037000
H	2.797677000	-0.849030000	-2.646632000
H	3.314659000	-2.109371000	-3.769733000
H	4.519253000	-1.251216000	-2.796226000
C	-3.373275000	-4.965315000	-2.526663000
H	-3.152764000	-5.571049000	-1.642565000
H	-3.016837000	-5.508859000	-3.409985000
H	-4.462921000	-4.872472000	-2.602935000
C	7.382775000	-1.471375000	1.836164000
H	7.556670000	-1.979367000	0.880053000
H	7.055967000	-2.225773000	2.559452000
H	8.338164000	-1.055076000	2.177730000
C	-5.020187000	-1.647340000	-0.092929000
C	1.547086000	-3.172684000	3.691321000
H	1.320135000	-3.945634000	2.955914000
H	0.746640000	-3.173224000	4.441070000
H	2.481898000	-3.444946000	4.196310000
C	0.617285000	2.857496000	3.826400000
H	-0.131796000	3.137348000	4.563609000
C	-2.977274000	-2.789370000	-3.738184000
H	-4.046599000	-2.757876000	-3.975794000
H	-2.471029000	-3.270740000	-4.583362000
H	-2.616115000	-1.761928000	-3.658512000
C	6.324500000	-0.364641000	1.677421000
H	6.186845000	0.106425000	2.659845000
C	1.955407000	3.197437000	4.030538000
H	2.255153000	3.739235000	4.923745000
C	2.905276000	2.832300000	3.074135000

H	3.952453000	3.086214000	3.220603000
C	6.815388000	0.721932000	0.712515000
H	7.741838000	1.174824000	1.084517000
H	6.070211000	1.513492000	0.588958000
H	7.031995000	0.304405000	-0.277856000
C	5.072114000	3.122051000	-2.536490000
H	6.078563000	3.340652000	-2.882295000
C	4.790908000	1.890141000	-1.939859000
H	5.571750000	1.147356000	-1.824903000
C	3.506289000	1.609917000	-1.484997000
H	3.275416000	0.667403000	-0.996758000
C	-6.425994000	-1.076406000	-0.182444000
H	-6.956021000	-1.662645000	-0.945672000
C	-6.387341000	0.381121000	-0.674415000
C	-7.223376000	-1.195770000	1.123003000
H	-7.239710000	-2.229112000	1.485255000
H	-6.798914000	-0.570702000	1.916791000
H	-8.257718000	-0.867952000	0.969598000
H	-5.888731000	1.016830000	0.063001000
H	-7.400369000	0.772676000	-0.827002000
H	-5.832206000	0.465076000	-1.613843000
N	-2.668606000	1.801554000	0.749373000
N	-2.830918000	1.243698000	-0.323790000
N	-2.868144000	0.681175000	-1.317530000
Si	-3.772857000	2.978492000	1.490316000
C	-2.697803000	4.196874000	2.422533000
H	-1.908328000	4.595303000	1.780118000
H	-3.307542000	5.035041000	2.782073000
H	-2.220397000	3.732668000	3.290428000
C	-4.767830000	3.841251000	0.146620000
H	-4.114446000	4.470444000	-0.464433000
H	-5.272285000	3.131497000	-0.517037000
H	-5.537168000	4.481699000	0.594977000
C	-4.900324000	2.083280000	2.703260000
H	-5.510053000	1.316588000	2.216907000
H	-4.313333000	1.597284000	3.489670000
H	-5.579797000	2.797192000	3.185622000

147
TS1, -5342.54465327

Ge	-0.053117000	-0.561291000	-0.265987000
P	0.850292000	2.103444000	-1.072205000
C	-1.100047000	-4.675931000	-0.160042000
H	-1.996761000	-5.280110000	-0.260766000
C	-1.226467000	-3.281657000	-0.128900000
C	0.210645000	-0.296819000	-2.170441000
C	0.147179000	-5.284828000	-0.045205000
H	0.232967000	-6.367433000	-0.078670000
C	0.962467000	1.700656000	1.877563000
C	0.731534000	1.437773000	-3.822027000
H	0.997997000	2.467189000	-4.046887000
C	-0.072668000	-2.482037000	0.016956000
C	0.514922000	1.035863000	-2.506293000
C	-2.604424000	-2.700101000	-0.138161000
C	-3.151552000	-2.236981000	1.075910000
C	0.152087000	-1.224797000	-3.210369000
H	-0.049940000	-2.267596000	-2.989854000
C	1.176450000	-3.100514000	0.211503000
C	2.610909000	2.526301000	-1.353576000
C	3.446411000	-2.204952000	-0.411862000
C	1.275430000	-4.498941000	0.163559000
H	2.244954000	-4.961736000	0.320146000
B	0.572774000	1.072330000	0.502287000
C	1.719955000	-0.980714000	4.109443000
H	2.520274000	-1.362648000	4.755446000
H	0.808517000	-0.938668000	4.714823000
H	1.970831000	0.036793000	3.811049000
C	2.417201000	-2.328419000	0.542865000
C	3.244073000	-2.666653000	-1.850520000
H	2.182179000	-2.895725000	-1.974363000
C	-3.378958000	-2.661047000	-1.313993000
C	-2.378381000	-2.304323000	2.384321000
H	-1.408213000	-2.761369000	2.184774000
C	-0.043231000	3.675217000	-1.257766000
C	4.039775000	-3.950960000	-2.137757000
H	5.116571000	-3.778210000	-2.023178000
H	3.861133000	-4.295324000	-3.163301000

H	3.757134000	-4.757293000	-1.453787000
C	0.355394000	-0.833435000	-4.537133000
H	0.300212000	-1.576343000	-5.328538000
C	1.491748000	-1.901852000	2.904971000
H	0.577611000	-1.563805000	2.407263000
C	3.046356000	3.746505000	-1.880237000
H	2.341091000	4.552664000	-2.048506000
C	-1.423935000	6.099623000	-1.450977000
H	-1.955320000	7.043516000	-1.531620000
C	2.604459000	-1.837000000	1.858946000
C	-3.084269000	-3.188180000	3.422816000
H	-4.068457000	-2.787166000	3.691335000
H	-2.486524000	-3.250789000	4.340153000
H	-3.229290000	-4.204051000	3.039323000
C	4.394921000	3.925685000	-2.196142000
H	4.731223000	4.878386000	-2.595667000
C	0.299259000	4.749488000	-0.423050000
H	1.095034000	4.635814000	0.307448000
C	0.634784000	0.495733000	-4.851283000
H	0.797650000	0.796438000	-5.881897000
C	-4.675981000	-2.150567000	-1.251587000
H	-5.276731000	-2.118535000	-2.157373000
C	-0.383980000	5.959184000	-0.528710000
H	-0.108819000	6.790485000	0.114066000
C	0.047791000	1.889230000	2.931268000
H	-0.982417000	1.597138000	2.778201000
C	-2.841250000	-3.148592000	-2.649306000
H	-1.772138000	-3.339517000	-2.523698000
C	4.685129000	-1.684175000	-0.015873000
H	5.491205000	-1.640171000	-0.743930000
C	4.917117000	-1.261770000	1.293645000
C	3.851913000	-1.311855000	2.196738000
H	4.017640000	-0.947438000	3.204544000
C	-4.445959000	-1.709920000	1.084882000
H	-4.853630000	-1.348306000	2.023983000
C	-2.119650000	-0.890899000	2.915963000
H	-1.679299000	-0.286105000	2.120950000
H	-1.433487000	-0.901987000	3.769638000
H	-3.048744000	-0.404787000	3.230869000
C	-1.782962000	5.022790000	-2.263824000
H	-2.596191000	5.125330000	-2.976736000
C	-1.096231000	3.812780000	-2.169901000
H	-1.377786000	2.974859000	-2.798895000
C	2.291215000	2.110819000	2.120214000
H	3.042310000	1.961960000	1.352353000
C	3.587745000	-1.576627000	-2.876873000
H	2.975082000	-0.686119000	-2.726865000
H	3.396730000	-1.943465000	-3.891507000
H	4.641863000	-1.281771000	-2.822059000
C	-3.498361000	-4.475058000	-3.065256000
H	-3.331020000	-5.254409000	-2.314190000
H	-3.090635000	-4.827784000	-4.020301000
H	-4.581520000	-4.354264000	-3.184435000
C	7.208221000	-2.083667000	1.893378000
H	7.374898000	-2.527660000	0.904688000
H	6.757434000	-2.851008000	2.531308000
H	8.184906000	-1.814151000	2.312984000
C	-5.226303000	-1.654923000	-0.069873000
C	1.251152000	-3.334162000	3.419272000
H	0.975156000	-4.026871000	2.623437000
H	0.444450000	-3.332785000	4.162156000
H	2.156321000	-3.717045000	3.906071000
C	0.427091000	2.452360000	4.148706000
H	-0.311741000	2.575572000	4.937138000
C	-3.000843000	-2.083690000	-3.746310000
H	-4.055758000	-1.920753000	-3.994455000
H	-2.490524000	-2.399118000	-4.663896000
H	-2.573024000	-1.131942000	-3.423517000
C	6.294924000	-0.848500000	1.787119000
H	6.162722000	-0.446454000	2.800372000
C	1.746107000	2.860229000	4.356326000
H	2.044136000	3.302409000	5.303166000
C	2.676992000	2.686523000	3.331445000
H	3.711489000	2.988430000	3.476843000
C	6.961195000	0.246657000	0.944758000
H	7.919534000	0.539334000	1.389323000
H	6.328596000	1.136338000	0.874140000
H	7.168972000	-0.104378000	-0.073205000

```
C        5.301666000      2.882310000     -2.013282000
H        6.347993000      3.018317000     -2.272052000
C        4.862054000      1.659295000     -1.500334000
H        5.555890000      0.839000000     -1.367570000
C        3.526154000      1.483341000     -1.156727000
H        3.177858000      0.541881000     -0.742011000
C       -6.607375000     -1.022020000     -0.095617000
H       -7.155277000     -1.481427000     -0.929924000
C       -6.488938000      0.483917000     -0.396365000
C       -7.422294000     -1.258793000      1.182382000
H       -7.482858000     -2.324969000      1.425172000
H       -6.980639000     -0.742153000      2.042392000
H       -8.441453000     -0.874619000      1.061612000
H       -5.971036000      0.995467000      0.421889000
H       -7.478195000      0.943480000     -0.510909000
H       -5.914458000      0.656728000     -1.312402000
N       -2.213162000      2.101088000      0.566478000
N       -2.599087000      1.212748000     -0.207323000
N       -2.456065000      0.270087000     -0.866420000
Si      -3.366133000      3.181809000      1.385504000
C       -2.346775000      4.533125000      2.185520000
H       -1.941950000      5.213943000      1.432665000
H       -2.969620000      5.115507000      2.875604000
H       -1.506265000      4.117893000      2.749663000
C       -4.562546000      3.889731000      0.115596000
H       -4.016664000      4.478876000     -0.628413000
H       -5.107106000      3.098201000     -0.410041000
H       -5.301329000      4.544334000      0.594016000
C       -4.291680000      2.196290000      2.699842000
H       -4.724722000      1.281363000      2.281727000
H       -3.614673000      1.901991000      3.509191000
H       -5.104967000      2.787430000      3.138492000


147
triazole, -5342.61690552
Ge      -0.425776000     -0.441317000     -0.317424000
P        1.010130000      2.249413000     -0.795337000
C       -1.850066000     -4.381571000     -0.890952000
H       -2.813327000     -4.857620000     -1.050212000
C       -1.818827000     -3.020417000     -0.561115000
C        0.199833000     -0.043063000     -2.113869000
C       -0.672443000     -5.114245000     -1.011072000
H       -0.706840000     -6.166943000     -1.277835000
C        1.045717000      1.190028000      2.123647000
C        1.163229000      1.680178000     -3.550711000
H        1.607053000      2.665124000     -3.664693000
C       -0.575967000     -2.384918000     -0.353177000
C        0.740626000      1.236274000     -2.288717000
C       -3.116029000     -2.298771000     -0.380731000
C       -3.557779000     -1.983294000      0.921320000
C        0.090026000     -0.873484000     -3.236057000
H       -0.320559000     -1.871198000     -3.120390000
C        0.607502000     -3.147779000     -0.378056000
C        2.820778000      2.514423000     -0.947496000
C        3.008394000     -2.429175000     -0.796859000
C        0.545675000     -4.504624000     -0.725928000
H        1.459426000     -5.089686000     -0.725852000
B        0.142692000      1.227474000      0.817407000
C        1.309594000     -2.088880000      3.898744000
H        2.067688000     -2.699042000      4.405368000
H        0.404397000     -2.125277000      4.514016000
H        1.655508000     -1.057974000      3.872075000
C        1.924606000     -2.605063000      0.093134000
C        2.871921000     -2.693357000     -2.292208000
H        1.806255000     -2.768958000     -2.519707000
C       -3.878103000     -1.903083000     -1.494931000
C       -2.759272000     -2.351024000      2.162599000
H       -1.859850000     -2.880897000      1.845666000
C        0.229674000      3.858893000     -1.130807000
C        3.533737000     -4.031994000     -2.668506000
H        4.611663000     -4.001155000     -2.470402000
H        3.392296000     -4.244957000     -3.734784000
H        3.116191000     -4.865856000     -2.097410000
C        0.477890000     -0.430298000     -4.496795000
H        0.371411000     -1.086151000     -5.356557000
C        0.995748000     -2.636385000      2.502803000
H        0.130962000     -2.083268000      2.127309000
```

C	3.449969000	3.759208000	-1.036698000
H	2.874488000	4.674019000	-0.969956000
C	-0.933391000	6.355368000	-1.650527000
H	-1.380371000	7.323544000	-1.855980000
C	2.122169000	-2.427239000	1.488895000
C	-3.537820000	-3.304748000	3.081288000
H	-4.459160000	-2.841062000	3.452203000
H	-2.929569000	-3.584100000	3.950272000
H	-3.815097000	-4.221019000	2.548803000
C	4.828927000	3.832176000	-1.250797000
H	5.307674000	4.804795000	-1.320098000
C	0.528536000	4.965126000	-0.321166000
H	1.203099000	4.857313000	0.520745000
C	1.016033000	0.851057000	-4.657910000
H	1.333182000	1.193128000	-5.638725000
C	-5.072314000	-1.210458000	-1.287055000
H	-5.652220000	-0.883483000	-2.146835000
C	-0.042828000	6.207766000	-0.585797000
H	0.201615000	7.056759000	0.045654000
C	0.455318000	1.316288000	3.395297000
H	-0.622974000	1.364438000	3.471500000
C	-3.386184000	-2.106097000	-2.919193000
H	-2.399811000	-2.575971000	-2.867848000
C	4.270290000	-2.102841000	-0.282576000
H	5.105537000	-2.003639000	-0.970669000
C	4.491214000	-1.953889000	1.086126000
C	3.401757000	-2.107479000	1.943478000
H	3.567308000	-1.976702000	3.005859000
C	-4.748203000	-1.274492000	1.078959000
H	-5.072483000	-1.011754000	2.082313000
C	-2.317938000	-1.084899000	2.913443000
H	-1.802020000	-0.399171000	2.237981000
H	-1.645354000	-1.328815000	3.742654000
H	-3.178103000	-0.545460000	3.323406000
C	-1.266055000	5.250121000	-2.434067000
H	-1.979563000	5.352007000	-3.246281000
C	-0.692768000	4.005294000	-2.175234000
H	-0.962635000	3.149772000	-2.783172000
C	2.444553000	1.053941000	2.105489000
H	2.956374000	0.887595000	1.168762000
C	3.449416000	-1.568863000	-3.164411000
H	2.973178000	-0.609377000	-2.960006000
H	3.288190000	-1.797438000	-4.223548000
H	4.529319000	-1.454663000	-3.015084000
C	-4.300449000	-3.050588000	-3.713583000
H	-4.368821000	-4.032334000	-3.231421000
H	-3.920098000	-3.194946000	-4.732200000
H	-5.316521000	-2.646130000	-3.789787000
C	6.732262000	-2.990649000	1.544892000
H	6.898659000	-3.243239000	0.490850000
H	6.238218000	-3.845420000	2.018412000
H	7.711583000	-2.851138000	2.018018000
C	-5.514975000	-0.865965000	-0.012660000
C	0.598502000	-4.118494000	2.648688000
H	0.226975000	-4.555112000	1.722312000
H	-0.187036000	-4.217059000	3.407058000
H	1.462254000	-4.707063000	2.981176000
C	1.210667000	1.394494000	4.564235000
H	0.708633000	1.506086000	5.521976000
C	-3.213812000	-0.750305000	-3.626912000
H	-4.181271000	-0.261387000	-3.787232000
H	-2.735089000	-0.880834000	-4.604811000
H	-2.597957000	-0.089147000	-3.013668000
C	5.874532000	-1.718704000	1.670289000
H	5.736231000	-1.515472000	2.740505000
C	2.603046000	1.322020000	4.505237000
H	3.198908000	1.390375000	5.411342000
C	3.214332000	1.132412000	3.265573000
H	4.295166000	1.034975000	3.196987000
C	6.601024000	-0.510873000	1.064544000
H	7.549836000	-0.335448000	1.584713000
H	5.995778000	0.398162000	1.133902000
H	6.835533000	-0.678680000	0.006943000
C	5.581871000	2.668656000	-1.394436000
H	6.651399000	2.728698000	-1.573898000
C	4.953556000	1.423192000	-1.313896000
H	5.523878000	0.510035000	-1.433870000
C	3.586340000	1.345081000	-1.081580000

H	3.107510000	0.374486000	-1.008831000
C	-6.750981000	-0.006006000	0.175975000
H	-7.191326000	0.154482000	-0.817477000
C	-6.362505000	1.371808000	0.742806000
C	-7.811573000	-0.689448000	1.053082000
H	-8.106493000	-1.657418000	0.633630000
H	-7.431703000	-0.866458000	2.066039000
H	-8.707744000	-0.063477000	1.138932000
H	-5.967773000	1.275771000	1.761052000
H	-7.231758000	2.039561000	0.781704000
H	-5.582742000	1.838339000	0.132374000
N	-1.217977000	1.902015000	0.930963000
N	-2.294281000	1.427766000	0.141187000
N	-2.173768000	0.381187000	-0.509929000
Si	-1.898716000	3.240100000	1.905030000
C	-0.524803000	4.251583000	2.731735000
H	-0.593093000	4.172404000	3.821930000
H	0.477259000	3.916354000	2.456885000
H	-0.623973000	5.309248000	2.462666000
C	-2.957824000	4.353477000	0.819253000
H	-2.414861000	4.731164000	-0.048307000
H	-3.831854000	3.804406000	0.457685000
H	-3.308970000	5.215351000	1.401752000
C	-3.072608000	2.535275000	3.208667000
H	-3.807760000	1.888970000	2.716916000
H	-2.574733000	1.947124000	3.985997000
H	-3.617971000	3.349237000	3.703539000

147
TS2, -5342.61409310

Ge	-0.423183000	-0.429224000	-0.325803000
P	1.311367000	2.316129000	-0.647571000
C	-2.113680000	-4.186481000	-1.276998000
H	-3.101451000	-4.582269000	-1.494879000
C	-2.000256000	-2.863673000	-0.830874000
C	0.299445000	0.113263000	-2.047985000
C	-0.983999000	-4.985136000	-1.435577000
H	-1.082237000	-6.005919000	-1.794403000
C	1.042522000	0.765829000	2.378181000
C	1.398628000	1.799775000	-3.418615000
H	1.931451000	2.743188000	-3.492099000
C	-0.721897000	-2.338163000	-0.546426000
C	0.970248000	1.339566000	-2.163136000
C	-3.243737000	-2.067138000	-0.595250000
C	-3.680944000	-1.849580000	0.729556000
C	0.066233000	-0.629613000	-3.214127000
H	-0.450148000	-1.580781000	-3.141378000
C	0.408977000	-3.175366000	-0.607624000
C	3.085605000	2.717428000	-0.946837000
C	2.829394000	-2.471148000	-0.924711000
C	0.265400000	-4.489647000	-1.072042000
H	1.137327000	-5.134789000	-1.105138000
B	0.008979000	0.865223000	1.211183000
C	1.103621000	-2.629838000	3.764270000
H	1.841833000	-3.301837000	4.218823000
H	0.189995000	-2.704639000	4.363787000
H	1.472825000	-1.608437000	3.840340000
C	1.742590000	-2.748446000	-0.066631000
C	2.708922000	-2.568662000	-2.439382000
H	1.646789000	-2.547901000	-2.690719000
C	-3.958882000	-1.508512000	-1.671164000
C	-2.961140000	-2.440657000	1.933204000
H	-2.088575000	-2.989207000	1.574363000
C	0.403106000	3.876575000	-0.939188000
C	3.289894000	-3.904345000	-2.939504000
H	4.358757000	-3.972688000	-2.705023000
H	3.175474000	-3.989880000	-4.026704000
H	2.792940000	-4.761919000	-2.477403000
C	0.468684000	-0.162868000	-4.461244000
H	0.266055000	-0.753498000	-5.350462000
C	0.796462000	-3.036171000	2.318105000
H	-0.054346000	-2.433988000	1.986707000
C	3.601979000	3.999375000	-1.167771000
H	2.942806000	4.859744000	-1.172888000
C	-1.024588000	6.267341000	-1.306500000
H	-1.572665000	7.193318000	-1.454020000
C	1.937582000	-2.749842000	1.340986000

C	-3.847061000	-3.450215000	2.679400000
H	-4.749546000	-2.971857000	3.077034000
H	-3.301197000	-3.892427000	3.521697000
H	-4.163206000	-4.260188000	2.013145000
C	4.965884000	4.179032000	-1.410029000
H	5.350975000	5.180441000	-1.582292000
C	0.678301000	5.003455000	-0.146802000
H	1.444474000	4.952892000	0.620612000
C	1.137897000	1.059657000	-4.566370000
H	1.462010000	1.426538000	-5.536178000
C	-5.090767000	-0.736145000	-1.402848000
H	-5.631796000	-0.283384000	-2.230112000
C	-0.020482000	6.193643000	-0.339281000
H	0.213445000	7.059765000	0.273174000
C	0.622332000	0.870621000	3.716442000
H	-0.435083000	0.960940000	3.933628000
C	-3.488712000	-1.649383000	-3.110213000
H	-2.540059000	-2.193542000	-3.099767000
C	4.087387000	-2.204765000	-0.369940000
H	4.920899000	-2.008706000	-1.037287000
C	4.306389000	-2.232410000	1.005472000
C	3.219357000	-2.507084000	1.835170000
H	3.383257000	-2.509609000	2.905910000
C	-4.806313000	-1.055671000	0.948201000
H	-5.125759000	-0.869677000	1.969955000
C	-2.460583000	-1.330352000	2.869495000
H	-1.879308000	-0.597857000	2.306841000
H	-1.833326000	-1.738708000	3.669124000
H	-3.292721000	-0.791910000	3.335935000
C	-1.339023000	5.137779000	-2.062800000
H	-2.139101000	5.178031000	-2.796309000
C	-0.635603000	3.947549000	-1.878374000
H	-0.893135000	3.072025000	-2.463256000
C	2.416646000	0.578432000	2.157582000
H	2.777152000	0.443655000	1.145588000
C	3.381697000	-1.401386000	-3.175135000
H	3.001955000	-0.437446000	-2.834001000
H	3.187479000	-1.474982000	-4.249996000
H	4.469180000	-1.408294000	-3.038761000
C	-4.476574000	-2.473710000	-3.950107000
H	-4.614842000	-3.475413000	-3.527775000
H	-4.114708000	-2.582733000	-4.979646000
H	-5.459916000	-1.990767000	-3.989339000
C	6.530879000	-3.308553000	1.450291000
H	6.695095000	-3.532502000	0.389479000
H	6.026952000	-4.170892000	1.899127000
H	7.510735000	-3.192824000	1.928708000
C	-5.516154000	-0.474036000	-0.103070000
C	0.366969000	-4.516128000	2.314450000
H	0.008173000	-4.850981000	1.341532000
H	-0.438320000	-4.670254000	3.042479000
H	1.210733000	-5.153202000	2.606073000
C	1.528178000	0.853123000	4.776449000
H	1.166612000	0.939485000	5.797771000
C	-3.222576000	-0.270680000	-3.738499000
H	-4.151981000	0.297009000	-3.859432000
H	-2.763171000	-0.379083000	-4.728110000
H	-2.552764000	0.306957000	-3.098287000
C	5.685819000	-2.031629000	1.608677000
H	5.543196000	-1.857881000	2.683576000
C	2.893927000	0.722396000	4.523194000
H	3.607123000	0.720835000	5.343055000
C	3.333329000	0.577119000	3.205833000
H	4.391352000	0.450331000	2.995551000
C	6.424453000	-0.815103000	1.034888000
H	7.370449000	-0.654789000	1.564498000
H	5.824846000	0.096738000	1.117184000
H	6.665239000	-0.961631000	-0.024567000
C	5.825681000	3.082723000	-1.448193000
H	6.884630000	3.224392000	-1.644369000
C	5.315841000	1.798932000	-1.239659000
H	5.973093000	0.936681000	-1.280175000
C	3.960611000	1.617469000	-0.980915000
H	3.574080000	0.612842000	-0.834985000
C	-6.671446000	0.473245000	0.162047000
H	-7.086626000	0.762303000	-0.812876000
C	-6.162495000	1.752833000	0.850897000
C	-7.797363000	-0.186390000	0.973335000

```
H       -8.174061000     -1.082977000      0.469314000
H       -7.445746000     -0.485066000      1.967726000
H       -8.634196000      0.508253000      1.112052000
H       -5.781885000      1.528295000      1.854458000
H       -6.967711000      2.490209000      0.953972000
H       -5.341146000      2.199545000      0.282036000
N       -1.170255000      1.675377000      1.285682000
N       -2.240254000      1.473307000      0.249272000
N       -2.098767000      0.550439000     -0.539167000
Si      -1.686192000      3.028716000      2.336356000
C       -0.169033000      3.897760000      3.057057000
H        0.735268000      3.659829000      2.490698000
H       -0.316218000      4.983680000      3.022444000
H        0.019162000      3.609909000      4.095206000
C       -2.713051000      4.236901000      1.330071000
H       -2.209919000      4.533738000      0.408955000
H       -3.675038000      3.793708000      1.058900000
H       -2.903517000      5.141743000      1.921669000
C       -2.823428000      2.362993000      3.690933000
H       -3.657573000      1.823882000      3.226917000
H       -2.333985000      1.679661000      4.391824000
H       -3.245278000      3.192683000      4.272429000


147
van der Waals complex of reaction products, -5342.64158412
Ge      -0.066280000     -0.653770000      0.160726000
P        1.280287000      2.141941000     -0.980732000
C       -2.180109000     -4.458675000     -0.308499000
H       -3.186921000     -4.857111000     -0.395790000
C       -2.006864000     -3.069762000     -0.214833000
C        0.127597000     -0.275016000     -1.819989000
C       -1.090258000     -5.325621000     -0.268683000
H       -1.243933000     -6.398661000     -0.340741000
C        1.597519000      1.989058000      2.399660000
C        0.877672000      1.204110000     -3.618679000
H        1.371999000      2.120832000     -3.926890000
C       -0.709745000     -2.542462000     -0.076799000
C        0.698940000      0.940675000     -2.251604000
C       -3.224536000     -2.202608000     -0.165495000
C       -3.599675000     -1.607782000      1.064073000
C       -0.315996000     -1.163731000     -2.813040000
H       -0.775221000     -2.099526000     -2.516888000
C        0.381002000     -3.427954000      0.008644000
C        3.022047000      2.319394000     -1.541698000
C        2.723797000     -2.677468000     -0.662451000
C        0.192388000     -4.809958000     -0.094738000
H        1.047778000     -5.474297000     -0.007810000
B        0.174434000      2.099050000      1.846325000
C        0.854794000     -1.633783000      3.643363000
H        1.780532000     -1.277551000      4.107393000
H        0.123590000     -1.812278000      4.439438000
H        0.475721000     -0.842418000      2.992664000
C        1.745197000     -2.886537000      0.338735000
C        2.432392000     -2.995142000     -2.121981000
H        1.347860000     -2.994671000     -2.246633000
C       -4.030960000     -2.012298000     -1.306932000
C       -2.797837000     -1.815494000      2.343413000
H       -1.840642000     -2.264776000      2.076021000
C        0.519231000      3.749730000     -1.424418000
C        2.942188000     -4.408155000     -2.461682000
H        4.029924000     -4.468663000     -2.337256000
H        2.704788000     -4.661271000     -3.501674000
H        2.487112000     -5.164142000     -1.815010000
C       -0.170887000     -0.889730000     -4.170422000
H       -0.526446000     -1.604478000     -4.907708000
C        1.077788000     -2.915047000      2.825823000
H        0.120531000     -3.187921000      2.377050000
C        3.600060000      3.487067000     -2.050296000
H        3.007405000      4.392107000     -2.136408000
C       -0.601554000      6.292634000     -1.858875000
H       -1.035202000      7.274000000     -2.028266000
C        2.081232000     -2.688929000      1.701463000
C       -3.516672000     -2.801227000      3.277758000
H       -4.494206000     -2.411258000      3.585221000
H       -2.922187000     -2.977441000      4.182286000
H       -3.679209000     -3.764049000      2.780852000
C        4.935829000      3.488249000     -2.459465000
```

H	5.375609000	4.400266000	-2.854599000
C	0.836185000	4.829813000	-0.580615000
H	1.511492000	4.673729000	0.257211000
C	0.441645000	0.296321000	-4.578822000
H	0.581542000	0.509306000	-5.635184000
C	-5.180461000	-1.223549000	-1.200880000
H	-5.798559000	-1.066745000	-2.081106000
C	0.291475000	6.092155000	-0.803160000
H	0.553952000	6.916680000	-0.146081000
C	1.967598000	3.033612000	3.270887000
H	1.238074000	3.798163000	3.522861000
C	-3.694603000	-2.631982000	-2.656777000
H	-2.695632000	-3.070837000	-2.586574000
C	4.022920000	-2.325236000	-0.279370000
H	4.779482000	-2.209324000	-1.048645000
C	4.383470000	-2.172584000	1.061606000
C	3.391348000	-2.337783000	2.029152000
H	3.656003000	-2.205577000	3.074847000
C	-4.762307000	-0.836763000	1.119958000
H	-5.046487000	-0.387363000	2.066674000
C	-2.482818000	-0.486822000	3.039711000
H	-1.973429000	0.194232000	2.353485000
H	-1.828242000	-0.654130000	3.900209000
H	-3.384802000	0.012255000	3.405409000
C	-0.950001000	5.220174000	-2.679105000
H	-1.660736000	5.362716000	-3.488537000
C	-0.396659000	3.956201000	-2.463074000
H	-0.685360000	3.130330000	-3.103351000
C	2.558988000	1.015075000	2.087920000
H	2.296465000	0.187361000	1.438981000
C	3.009652000	-1.968359000	-3.104541000
H	2.650914000	-0.960974000	-2.890330000
H	2.704648000	-2.221145000	-4.125318000
H	4.105366000	-1.952312000	-3.084555000
C	-4.670905000	-3.770092000	-3.001258000
H	-4.657530000	-4.551597000	-2.234589000
H	-4.407763000	-4.228934000	-3.961761000
H	-5.698176000	-3.394811000	-3.076088000
C	6.568742000	-3.303612000	1.509820000
H	6.617042000	-3.725556000	0.499021000
H	6.057238000	-4.028937000	2.151012000
H	7.594394000	-3.179263000	1.876580000
C	-5.565678000	-0.624466000	-0.002108000
C	1.507892000	-4.093075000	3.714793000
H	1.631966000	-5.006218000	3.122344000
H	0.751840000	-4.284455000	4.484973000
H	2.458008000	-3.888398000	4.221303000
C	3.255002000	3.108940000	3.801262000
H	3.520151000	3.926889000	4.465539000
C	-3.665971000	-1.582772000	-3.780620000
H	-4.663324000	-1.172663000	-3.974221000
H	-3.310424000	-2.034906000	-4.714053000
H	-3.000253000	-0.752583000	-3.530329000
C	5.824237000	-1.954858000	1.495450000
H	5.796653000	-1.585564000	2.529787000
C	4.197071000	2.134723000	3.473635000
H	5.203535000	2.188755000	3.879963000
C	3.841915000	1.087205000	2.620804000
H	4.566669000	0.323746000	2.366116000
C	6.584092000	-0.924954000	0.651046000
H	7.578439000	-0.746861000	1.075485000
H	6.054240000	0.030540000	0.601173000
H	6.729431000	-1.282537000	-0.374731000
C	5.697398000	2.322914000	-2.380612000
H	6.731627000	2.322154000	-2.713300000
C	5.123767000	1.154487000	-1.871300000
H	5.707228000	0.241975000	-1.813961000
C	3.802437000	1.156404000	-1.436503000
H	3.353968000	0.252775000	-1.033271000
C	-6.813657000	0.238267000	0.066398000
H	-7.302656000	0.177233000	-0.915180000
C	-6.460584000	1.713961000	0.319390000
C	-7.810425000	-0.278596000	1.116372000
H	-8.078207000	-1.323421000	0.926323000
H	-7.387915000	-0.219511000	2.125946000
H	-8.728293000	0.320508000	1.105172000
H	-5.987055000	1.842745000	1.298583000
H	-7.362146000	2.337596000	0.299220000

```
H       -5.764839000      2.088291000     -0.438122000
N       -1.008866000      2.520890000      1.661207000
N       -2.995541000      1.212706000     -0.909305000
N       -2.194764000      0.579243000     -0.488180000
Si      -2.262911000      3.667172000      1.918877000
C       -1.523525000      5.160739000      2.814199000
H       -1.080769000      4.867140000      3.773597000
H       -0.735208000      5.615416000      2.202715000
H       -2.278793000      5.930709000      3.014966000
C       -3.038591000      4.269415000      0.313017000
H       -2.279337000      4.610631000     -0.394748000
H       -3.634385000      3.493777000     -0.176284000
H       -3.706235000      5.114776000      0.524114000
C       -3.623579000      2.905828000      2.981899000
H       -4.065353000      2.044104000      2.471783000
H       -3.238702000      2.561134000      3.947933000
H       -4.423107000      3.632654000      3.171974000


2
N2 molecule, -109.524881221
N        0.000000000      0.000000000      0.552748000
N        0.000000000      0.000000000     -0.552748000


26
iminoborane, -720.637221285
C        1.797675000     -0.000535000     -0.000298000
B        0.277160000     -0.000685000     -0.000533000
C        2.517474000     -1.210161000     -0.000137000
H        1.980083000     -2.154141000     -0.000260000
C        2.516935000      1.209432000     -0.000161000
H        1.979112000      2.153170000     -0.000306000
C        3.910408000     -1.209426000      0.000176000
H        4.452434000     -2.150772000      0.000306000
C        4.607953000      0.000088000      0.000331000
H        5.694422000      0.000326000      0.000591000
C        3.909871000      1.209297000      0.000153000
H        4.451485000      2.150881000      0.000265000
N       -0.979847000     -0.000259000     -0.000734000
Si      -2.702158000      0.000008000      0.000017000
C       -3.321438000     -0.742280000      1.618083000
H       -2.967917000     -1.772480000      1.740236000
H       -2.967194000     -0.163167000      2.478456000
H       -4.417841000     -0.757303000      1.651228000
C       -3.317664000      1.773626000     -0.165739000
H       -2.962099000      2.393673000      0.665129000
H       -2.963770000      2.228215000     -1.098106000
H       -4.413937000      1.812269000     -0.168235000
C       -3.322163000     -1.029892000     -1.451675000
H       -2.968388000     -0.620760000     -2.404851000
H       -2.968775000     -2.064918000     -1.380394000
H       -4.418575000     -1.050766000     -1.480898000


119
compound 1, -4512.43888260
Ge       0.413176000     -0.288206000     -0.566877000
P       -2.304875000      1.182033000     -0.110391000
C        3.643204000     -3.032137000      0.045620000
H        4.729425000     -3.035311000      0.058795000
C        2.938844000     -1.826976000     -0.041026000
C        0.074002000      0.548632000      1.200444000
C        2.945483000     -4.243315000      0.109120000
H        3.498192000     -5.176659000      0.171419000
C       -1.374942000      2.036541000      2.478100000
H       -2.282386000      2.624476000      2.563757000
C        1.537317000     -1.854987000     -0.073652000
C       -1.109830000      1.314495000      1.311847000
C        3.486263000     -0.434427000     -0.047891000
C        3.114924000      0.417266000     -1.129222000
C        0.943959000      0.512531000      2.293132000
H        1.837997000     -0.095355000      2.233181000
C        0.841713000     -3.063818000     -0.009403000
C       -3.683270000      2.275856000      0.447494000
C       -1.444987000     -2.735789000      1.033793000
C        1.547492000     -4.266843000      0.087056000
H        1.014496000     -5.213389000      0.128610000
```

C	-0.754033000	-1.890981000	-3.686283000
H	-1.747952000	-2.036047000	-4.125179000
H	-0.027473000	-1.887961000	-4.507112000
H	-0.727904000	-0.915610000	-3.192202000
C	-0.648762000	-2.935673000	-0.121002000
C	-0.800659000	-2.700332000	2.412479000
H	0.204499000	-2.287978000	2.280036000
C	4.091558000	0.116999000	1.098622000
C	2.854565000	-0.166782000	-2.523780000
H	2.238950000	-1.064217000	-2.417711000
C	-1.460994000	2.250733000	-1.354334000
C	-0.657990000	-4.122545000	2.982948000
H	-1.643007000	-4.589883000	3.101588000
H	-0.172526000	-4.097182000	3.965889000
H	-0.058414000	-4.757635000	2.324336000
C	0.682424000	1.233296000	3.460745000
H	1.372553000	1.185740000	4.299426000
C	-0.403022000	-2.989615000	-2.670571000
H	0.640491000	-2.833695000	-2.383983000
C	-3.993199000	3.503511000	-0.152637000
H	-3.365577000	3.887656000	-0.949298000
C	-0.179474000	3.770404000	-3.334479000
H	0.317316000	4.359097000	-4.100568000
C	-1.242608000	-2.880524000	-1.404068000
C	4.197656000	-0.611903000	-3.132550000
H	4.877860000	0.241596000	-3.237037000
H	4.041255000	-1.048789000	-4.125905000
H	4.685995000	-1.361191000	-2.501268000
C	-5.102109000	4.239957000	0.268835000
H	-5.327833000	5.189821000	-0.208679000
C	-1.643584000	1.951630000	-2.710594000
H	-2.272262000	1.109962000	-2.988776000
C	-0.474343000	2.003686000	3.547751000
H	-0.688019000	2.572891000	4.448684000
C	4.202665000	1.507566000	1.195384000
H	4.649005000	1.942872000	2.084950000
C	-1.014403000	2.710509000	-3.696211000
H	-1.162848000	2.467503000	-4.744617000
C	4.623745000	-0.771437000	2.216193000
H	4.053184000	-1.705400000	2.195085000
C	-2.826150000	-2.610644000	0.884183000
H	-3.441870000	-2.483190000	1.768500000
C	-3.440189000	-2.636932000	-0.372669000
C	-2.630158000	-2.743371000	-1.500027000
H	-3.094643000	-2.720762000	-2.482230000
C	3.170331000	1.805005000	-0.942877000
H	2.824389000	2.451372000	-1.739536000
C	2.109668000	0.781648000	-3.467541000
H	1.205422000	1.175014000	-2.997706000
H	1.814106000	0.241923000	-4.373509000
H	2.734464000	1.627629000	-3.778303000
C	0.022098000	4.064175000	-1.984428000
H	0.667899000	4.888880000	-1.696190000
C	-0.609817000	3.303289000	-1.000680000
H	-0.454258000	3.526527000	0.049468000
C	-1.535758000	-1.790587000	3.405231000
H	-1.700575000	-0.795917000	2.984135000
H	-0.938538000	-1.676295000	4.316082000
H	-2.505059000	-2.208940000	3.701881000
C	6.100288000	-1.118655000	1.946053000
H	6.228826000	-1.582185000	0.962917000
H	6.484619000	-1.808805000	2.706428000
H	6.716519000	-0.211757000	1.966041000
C	-5.632112000	-3.787172000	0.094063000
H	-5.445383000	-3.845345000	1.172819000
H	-5.255058000	-4.709840000	-0.359974000
H	-6.717248000	-3.746038000	-0.057116000
C	3.689395000	2.368101000	0.225591000
C	-0.512302000	-4.394788000	-3.282195000
H	-0.188546000	-5.158776000	-2.567072000
H	0.113319000	-4.477791000	-4.178820000
H	-1.547031000	-4.617773000	-3.568199000
C	4.473811000	-0.176037000	3.624124000
H	5.119564000	0.696769000	3.772003000
H	4.760700000	-0.921148000	4.374275000
H	3.443282000	0.129787000	3.827233000
C	-4.949451000	-2.550160000	-0.514334000
H	-5.173031000	-2.541149000	-1.589557000

```
C       -5.512269000     -1.254969000      0.088292000
H       -6.590392000     -1.177558000     -0.094567000
H       -5.021415000     -0.376638000     -0.338498000
H       -5.358271000     -1.226700000      1.173136000
C       -5.912574000      3.767526000      1.300509000
H       -6.772741000      4.344289000      1.628737000
C       -5.613204000      2.545412000      1.907441000
H       -6.238925000      2.166829000      2.711177000
C       -4.516183000      1.803324000      1.476980000
H       -4.295118000      0.849461000      1.948029000
C        3.672623000      3.862487000      0.495257000
H        4.527776000      4.080591000      1.150132000
C        2.390776000      4.229449000      1.267096000
C        3.823250000      4.722385000     -0.765463000
H        4.720148000      4.448517000     -1.331660000
H        2.959836000      4.613844000     -1.430551000
H        3.898032000      5.781757000     -0.496150000
H        1.509833000      4.007840000      0.658476000
H        2.376894000      5.295758000      1.522866000
H        2.300865000      3.646517000      2.189029000
```

References

[1] a) R. S. Simons, L. Pu, M. M. Olmstead, P. P. Power, *Organometallics* **1997**, *16*, 1920-1925; b) S. Akiyama, K. Yamada, M. Yamashita, *Angew. Chem. Int. Ed. Engl.* **2019**, *58*, 11806-11810; c) J. Schneider, K. M. Krebs, S. Freitag, K. Eichele, H. Schubert, L. Wesemann, *Chemistry* **2016**, *22*, 9812-9826; d) D. Raiser, C. P. Sindlinger, H. Schubert, L. Wesemann, *Angew. Chem. Int. Ed. Engl.* **2020**, *59*, 3151-3155.

[2] R. K. Harris, E. D. Becker, S. M. Cabral de Menezes, R. Goodfellow, P. Granger, *Pure Appl. Chem.* **2001**, *73*, 1795-1818.

[3] a) L. J. Farrugia, *J. Appl. Crystallogr.* **1999**, *32*, 837-838; b) L. J. Farrugia, *J. Appl. Crystallogr.* **2012**, *45*, 849-854; c) C. B. Hubschle, G. M. Sheldrick, B. Dittrich, *J. Appl. Crystallogr.* **2011**, *44*, 1281-1284; d) G. M. Sheldrick, *Acta Crystallogr A* **2008**, *64*, 112-122; e) G. M. Sheldrick, University of Göttingen, Germany, **2008**; f) Bruker, AXS, Inc., Madison, Wisconsin, USA, **2012**.

[4] a) K. Fukui, *Acc. Chem. Res.* **1981**, *14*, 363-368; b) M. Page, J. W. McIver, *J. Chem. Phys.* **1988**, *88*, 922-935; c) M. Page, C. Doubleday, J. W. McIver, *J. Chem. Phys.* **1990**, *93*, 5634-5642.

[5] Gaussian 16 Rev. C.01, M. J. Frisch, G. W. Trucks, H. B. Schlegel, G. E. Scuseria, M. A. Robb, J. R. Cheeseman, G. Scalmani, V. Barone, G. A. Petersson, H. Nakatsuji, X. Li, M. Caricato, A. V. Marenich, J. Bloino, B. G. Janesko, R. Gomperts, B. Mennucci, H. P. Hratchian, J. V. Ortiz, A. F. Izmaylov, J. L. Sonnenberg, Williams, F. Ding, F. Lipparini, F. Egidi, J. Goings, B. Peng, A. Petrone, T. Henderson, D. Ranasinghe, V. G. Zakrzewski, J. Gao, N. Rega, G. Zheng, W. Liang, M. Hada, M. Ehara, K. Toyota, R. Fukuda, J. Hasegawa, M. Ishida, T. Nakajima, Y. Honda, O. Kitao, H. Nakai, T. Vreven, K. Throssell, J. A. Montgomery Jr., J. E. Peralta, F. Ogliaro, M. J. Bearpark, J. J. Heyd, E. N. Brothers, K. N. Kudin, V. N. Staroverov, T. A. Keith, R. Kobayashi, J. Normand, K. Raghavachari, A. P. Rendell, J. C. Burant, S. S. Iyengar, J. Tomasi, M. Cossi, J. M. Millam, M. Klene, C. Adamo, R. Cammi, J. W. Ochterski, R. L. Martin, K. Morokuma, O. Farkas, J. B. Foresman, D. J. Fox, Wallingford, CT, **2016**.

[6] a) F. Neese, *WIREs Comput. Mol. Sci.* **2012**, *2*, 73-78; b) F. Neese, *WIREs Computational Molecular Science* **2018**, *8*, e1327.
